# Using a Human Challenge Model of Infection to Measure Vaccine Efficacy: A Randomised, Controlled Trial Comparing the Typhoid Vaccines M01ZH09 with Placebo and Ty21a

**DOI:** 10.1371/journal.pntd.0004926

**Published:** 2016-08-17

**Authors:** Thomas C. Darton, Claire Jones, Christoph J. Blohmke, Claire S. Waddington, Liqing Zhou, Anna Peters, Kathryn Haworth, Rebecca Sie, Christopher A. Green, Catherine A. Jeppesen, Maria Moore, Ben A. V. Thompson, Tessa John, Robert A. Kingsley, Ly-Mee Yu, Merryn Voysey, Zoe Hindle, Stephen Lockhart, Marcelo B. Sztein, Gordon Dougan, Brian Angus, Myron M. Levine, Andrew J. Pollard

**Affiliations:** 1 Oxford Vaccine Group, Department of Paediatrics, and the NIHR Oxford Biomedical Research Centre, University of Oxford, Oxford, United Kingdom; 2 Nuffield Department of Primary Care Health Sciences, University of Oxford, Oxford, United Kingdom; 3 Microbial Pathogenesis Group, Wellcome Trust Sanger Institute, Hinxton, United Kingdom; 4 Emergent Product Development UK Ltd, Emergent BioSolutions, Wokingham, United Kingdom; 5 Center for Vaccine Development, University of Maryland School of Medicine, Baltimore, Maryland, United States of America; 6 Nuffield Department of Medicine, University of Oxford, Oxford, United Kingdom; Fondation Raoul Follereau, FRANCE

## Abstract

**Background:**

Typhoid persists as a major cause of global morbidity. While several licensed vaccines to prevent typhoid are available, they are of only moderate efficacy and unsuitable for use in children less than two years of age. Development of new efficacious vaccines is complicated by the human host-restriction of *Salmonella* enterica serovar Typhi (*S*. Typhi) and lack of clear correlates of protection. In this study, we aimed to evaluate the protective efficacy of a single dose of the oral vaccine candidate, M01ZH09, in susceptible volunteers by direct typhoid challenge.

**Methods and Findings:**

We performed a randomised, double-blind, placebo-controlled trial in healthy adult participants at a single centre in Oxford (UK). Participants were allocated to receive one dose of double-blinded M01ZH09 or placebo or 3-doses of open-label Ty21a. Twenty-eight days after vaccination, participants were challenged with 10^4^CFU *S*. Typhi Quailes strain. The efficacy of M01ZH09 compared with placebo (primary outcome) was assessed as the percentage of participants reaching pre-defined endpoints constituting typhoid diagnosis (fever and/or bacteraemia) during the 14 days after challenge. Ninety-nine participants were randomised to receive M01ZH09 (n = 33), placebo (n = 33) or 3-doses of Ty21a (n = 33). After challenge, typhoid was diagnosed in 18/31 (58.1% [95% CI 39.1 to 75.5]) M01ZH09, 20/30 (66.7% [47.2 to 87.2]) placebo, and 13/30 (43.3% [25.5 to 62.6]) Ty21a vaccine recipients. Vaccine efficacy (VE) for one dose of M01ZH09 was 13% [95% CI -29 to 41] and 35% [-5 to 60] for 3-doses of Ty21a. Retrospective multivariable analyses demonstrated that pre-existing anti-Vi antibody significantly reduced susceptibility to infection after challenge; a 1 log increase in anti-Vi IgG resulting in a 71% decrease in the hazard ratio of typhoid diagnosis ([95% CI 30 to 88%], p = 0.006) during the 14 day challenge period. Limitations to the study included the requirement to limit the challenge period prior to treatment to 2 weeks, the intensity of the study procedures and the high challenge dose used resulting in a stringent model.

**Conclusions:**

Despite successfully demonstrating the use of a human challenge study to directly evaluate vaccine efficacy, a single-dose M01ZH09 failed to demonstrate significant protection after challenge with virulent Salmonella Typhi in this model. Anti-Vi antibody detected prior to vaccination played a major role in outcome after challenge.

**Trial registration:**

ClinicalTrials.gov (NCT01405521) and EudraCT (number 2011-000381-35).

## Author Summary

Typhoid fever is a common cause of febrile illness in tropical countries. Although currently available typhoid vaccines are moderately effective, they are not suitable for use in young children. Development of new vaccines is complicated as *Salmonella* Typhi, the causative bacteria, only infect humans. In this study, we used a recently developed human typhoid challenge model to directly assess the efficacy of a new oral vaccine candidate, M01ZH09, compared to placebo. A parallel group of participants were given 3-doses of licensed oral Ty21a vaccine as a positive comparator. We found that a single dose of M01ZH09 was not effective in preventing typhoid infection in our model, although significant effects were seen in delaying onset of infection and reducing bacterial numbers. Ty21a also failed to significantly protect against infection suggesting our model was particularly stringent. We discovered that anti-Vi antibodies, present in some individuals prior to vaccination, contributed significantly to preventing infection in some individuals, and when this effect was taken into account, M01ZH09 halved the risk of developing typhoid after being challenged. These results demonstrate the utility of human challenge models in assessing the efficacy of new typhoid vaccine candidates, and suggest that further development of M01ZH09 dosing or delivery strategies may produce better results. These results also support further development of Vi-based vaccines as a potentially preventive intervention.

## Introduction

Typhoid fever, caused by *Salmonella enterica* serovar Typhi (*S*. Typhi), continues to be a major cause of global morbidity and poverty, particularly in areas without basic sanitation and limited access to clean water, and among non-immune travellers to those settings [1–3]. Despite causing an estimated 22 million new cases each year, vaccination to prevent typhoid infection has been little implemented [4, 5]. Licensed vaccines have demonstrated moderate efficacy in preventing infection in older children and adults, but are not suitable for use in young children and infants less than 2 years of age [6, 7]. Development of new efficacious vaccines is complicated by the human host-restriction of *S*. Typhi, the lack of clear correlates of protection, the scale required to run field trials of efficacy and uncertainty about estimation of vaccine impact due to suboptimal diagnostics. A human challenge model can be used to overcome some of these difficulties and can provide some direct estimation of efficacy in vaccine recipients who are deliberately exposed to the pathogen in a controlled setting [8].

M01ZH09 is a live attenuated oral vaccine constructed from the parent Ty2 strain by defined, independently attenuated deletion of the *ssaV* and *aroC* genes [9]. A single dose of M01ZH09 vaccine has proven to be well-tolerated and highly immunogenic in six previous phase I and IIa studies [10–13]. In particular, high levels of anti-lipopolysaccharide (LPS) antibodies were generated in response to vaccination in studies conducted in both low- and high-endemicity areas and in diverse age groups [10–13]. Evidence to support anti-LPS response as a useful protective parameter is limited, and mostly derived from observations made in endemic settings [14, 15]. Evaluation of typhoid vaccines in previous human challenge studies has been instrumental in their development, notably for Ty21a, which is also a live attenuated vaccine derived from Ty2 but does not constitutively express the Vi (Virulence) capsular polysaccharide and contains multiple additional genetic attenuations [16–18].

The aim of this study was to assess whether a single dose of oral M01ZH09 could protect healthy adult volunteers against developing typhoid infection in a challenge model, 28 days after vaccination. In our recently developed challenge model, ingestion of 10^4^ CFU virulent *S*. Typhi Quailes strain bacteria resulted in a 65% attack rate in unvaccinated adult volunteers [19]. Suitability of the model for vaccine efficacy (VE) evaluation was assessed in parallel by using the 3-dose schedule of oral Ty21a vaccine as an open-label comparator group.

## Methods

### Study design

A randomised, double-blind, placebo-controlled trial was performed at the Oxford Vaccine Group in the Centre for Clinical Vaccinology and Tropical Medicine (Churchill Hospital, Oxford, UK) to assess the protective efficacy of a single dose of M01ZH09 compared to placebo against *S*. Typhi challenge 28 days after vaccination.

This phase 2b trial was sponsored and monitored by the Oxford University Clinical Trials and Research Governance Department, approved by NRES South Central–Oxford A (11/SC/0302) and conducted in accordance with the principles of the International Conference of Harmonisation, Good Clinical Practice guidelines. After study initiation (November 2011), an independent Data and Safety Monitoring Committee reviewed clinical and laboratory data relating to patient safety (months 1, 5 and 8) and interim unblinded analyses of VE (months 5 and 8). No changes to the study protocol or participant eligibility were recommended.

### Participants

Potential participants from the community were approached using a variety of media including postal leaflets, e-mails, advertising posters and local newspaper and football programme advertising. Interested individuals were then invited to contact the study centre for further discussion and to receive written study information prior to invitation for eligibility assessment and enrolment. Eligible participants were healthy men and non-pregnant women, aged 18 to 60 years with no previous history of typhoid vaccination, infection or likely exposure to *S*. Typhi. All eligible volunteers were provided with detailed pre-study counselling and provided written informed consent. Following consent, participants were thoroughly evaluated for health problems by history, physical examination, blood screening and ultrasound examination of the gall bladder. A full description of the inclusion/exclusion criteria can be found in the study protocol (**S1 Protocol**).

### Randomisation and masking

We randomised participants to a double blind or open-label (Ty21a) arm in a 2:1 ratio, with further randomisation of the double blind arm to M01ZH09 or placebo (1:1 ratio). Randomisation lists were computer generated by permuted block randomisation with variable block sizes. Blinding was effected through identical packaging and allocation concealment by sequentially numbered sealed opaque envelopes. An independent study statistician (LMY) generated the randomisation sequence and provided sealed envelopes containing allocation codes. Participants and study staff remained unaware of group allocation until four weeks after the completion of the challenge phase, at which point specific, unblinded study team members revealed vaccine assignment to the participant only. These study team members took no part in performing the laboratory assays or data analysis.

### Procedures

A single dose of the study vaccine, M01ZH09 (Emergent BioSolutions, Wokingham, UK), containing 1x10^10^ CFU of live attenuated *S*. Typhi (Ty2 Δ*aroC* Δ*ssaV*) ZH09 strain was re-suspended in 20mL NaHCO_3_[aq] solution prior to ingestion. Placebo, containing excipients only (M9S basal medium plus 10%(w/w) sucrose), was re-suspended in an identical fashion. Participants were fasted for one hour before and after vaccine ingestion. Participants in the open-label arm ingested three capsules of enteric-coated Ty21a vaccine (Crucell UK Ltd, High Wycombe, UK), containing not less than 2x10^9^ CFU on alternate days, in accordance with manufacturer’s instructions [20].

### Assessment of immune responses to vaccination

Antibody secreting cell (ASC) responses to LPS, flagellin and Vi were measured at baseline and 7 days after vaccination by ELISpot assay. Briefly, peripheral blood mononuclear cells (PBMC) separated from venous blood, were dispensed in concentrations of 2.5 or 5.0x10^6^ cells/mL to nitrocellulose plates pre-coated with lipopolysaccharide (*S*. Typhosa LPS, L2387; Sigma-Aldrich, Dorset, UK), Vi (Sanofi Pasteur, Maidenhead, UK), flagellin (prepared by isolation from *S*. Typhi Quailes strain and purification at the University of Maryland School of Medicine) or buffer only (negative control). After overnight incubation and wash steps, alkaline-phosphatase goat anti-human IgG, IgM and IgA antibodies were added. After further incubation spots were developed with alkaline phosphatase substrates. Spots were manually counted by two independent observers and expressed as spots/1x10^6^ PBMC.

Antibody responses were measured 28 days after vaccination (immediately prior to *S*. Typhi challenge) and compared with those collected at baseline (pre-vaccination). Specific immunoglobulin G (IgG), IgA and IgM isotype responses to LPS and flagellin were measured in serum as previously described [19]. In addition, immunoglobulin G (IgG) responses to Vi were measured using a commercial ELISA kit (VaccZyme, The Binding Site Ltd, Birmingham, UK) according to the manufacturer’s instructions.

### *S*. Typhi challenge

Four weeks after vaccination participants were challenged with *S*. Typhi, Quailes strain, as described previously [19]; day 0 was defined as the day of challenge. A target challenge dose of 1-5x10^4^ CFU was used to achieve a 65% attack rate in participants allocated to placebo (**S1 Fig**). After fasting for 90 minutes participants ingested 1·2g/120mL NaHCO_3_ [aq] followed two minutes later by the challenge inoculum suspended in 0·53g/30mL NaHCO_3_ [aq]. Following challenge, participants were observed for a further 90 minutes prior to leaving the clinic.

### Outcomes

Participants were reviewed at least daily at the study site for 14 days, except for days 2 and 4 after challenge when they were telephoned twice instead, or if additional visits were required. Assessments performed included clinical evaluation and microbiological assay of blood and stool samples as described below and in the study protocol (**S1 Protocol**).

The primary study objective was to assess the efficacy of M01ZH09 or Ty21a vaccines compared with placebo in preventing infection during the 2-weeks after *S*. Typhi challenge. Typhoid diagnosis (TD) was defined as either, a) oral temperature ≥38°C sustained for ≥12 hours or more after day 5 of challenge, b) a blood culture positive for *S*. Typhi taken after day 7 of challenge, or c) a blood culture positive for *S*. Typhi collected after day 5 *plus* objective symptoms or signs (including fever) of typhoid infection.

Severe typhoid fever was defined as a case fulfilling the criteria for TD with the addition of one or more of the following features: oral temperature recorded ≥40°C, systolic blood pressure ≤85mmHg, significant lethargy or confusion, a GI bleeding event or suspected/confirmed perforation, or any Grade 4 (‘life threatening’) laboratory abnormality.

### Typhoid diagnosis procedures

Participants fulfilling the criteria for typhoid diagnosis were assessed by a physician and initiated on antibiotic treatment and other medication required for symptom control. Antibiotic treatment given either at TD or at day 14 (in those not developing features of infection) was ciprofloxacin 500mg twice daily for 14 days (first-line), or azithromycin 500mg once daily for 7 days (second-line). Following diagnosis, participants were reassessed at 6, 12, 24, 48, 72 and 96 hours, to ensure resolution of clinical symptoms and bacteriological cure. In the event that a first positive blood culture result was received beyond Day 14 after challenge (and thus after commencement of antibiotic treatment), a TD assessment was made and further visits were arranged as determined by the study investigator. Compliance with antibiotic treatment was determined by direct observation at each study visit and by daily telephone/text message reminders. Following completion of an antibiotic course, clearance of the challenge strain was confirmed by microbiological culture of at least two stool samples obtained at least one week apart, collected at least 3 weeks after completion of antibiotics.

### Haematological, biochemical and microbiological assays after challenge

Routine haematological and biochemical monitoring was performed using blood samples collected at challenge and at each visit thereafter. Blood (10mL) and stool samples were collected for bacterial culture at each visit, while quantitative blood culture was performed using 10mL of blood at the TD visit only. Cultures were performed by the local hospital accredited pathology laboratories according to national standard operating procedures [21–24], and as previously described [19].

### Participant clinical and safety data

In addition to regular clinical review, participants collected symptom data (solicited for headache, feeling generally unwell, loss of appetite, abdominal pain, nausea/vomiting, myalgia, arthralgia, cough, diarrhoea and constipation and any unsolicited symptoms) daily and twice-daily self-recorded oral temperature readings using supplied written diary cards for 7 days after vaccine and 28 days after challenge ingestion. Data from diary cards was confirmed and clarified with the participant at each review.

Safety measures instituted included 24-hour contact with a study doctor, involvement of the participant’s general practitioner, notification of the participant’s close-contacts of involvement in the study, and provision of a 24-hour emergency contact who could be approached if the participant could not be contacted. All screened individuals were consented for inclusion onto The Over-volunteering Prevention System Database (TOPS)[25].

### Statistical methods

Based on previous findings a challenge inoculum of 1-5x10^4^ CFU was used to achieve an attack rate of 60–75%. Assuming a similar attack rate in the placebo group using the same TD definitions, then to demonstrate a protective effect of 83%, resulting in a reduction in attack rate to 10%, 21 participants would be needed per group. If the attack rate in the placebo group were to fall to 50%, then 30 participants would be needed per group to demonstrate a protective effect of vaccination of 80% with 90% power (1-β) at the 5% significance level (α). To include an additional 10% dropout rate, target enrollment was 33 individuals per group.

Statistical comparisons were made between vaccine groups and placebo using the *per protocol* population, which included all participants completing the 14-day challenge period. The study was not powered to compare the two active vaccine groups with each other and thus it was pre-specified that these comparisons would not be performed.

The frequencies of TD in M01ZH09 (primary endpoint) or Ty21a recipients were compared with placebo to calculate vaccine protective efficacy (VE, i.e. the percentage reduction in attack rate in those vaccinated compared to the unvaccinated group) and presented along with 95% confidence intervals. Sensitivity analyses compared VE under different definitions of typhoid fever. Time to diagnosis (hours), time to development of fever (≥38°C, hours), and time to bacteraemia (point at which blood culture was collected, hours) were summarised using the Kaplan-Meier method. A *post hoc* proportional hazards model assessed factors associated with time to typhoid infection or time to bacteraemia, including vaccine receipt, baseline antibody status and age, sex and travel to endemic regions.

Vaccine immunogenicity was assessed by log_10_-transformation of antibody levels measured by ELISA and ASC counts (with 0 counts given a nominal value of 0.25). Comparisons between groups were analysed using analysis of covariance (ANCOVA) models to adjust for pre-vaccination titres. Comparisons of bacterial load and other non-normally distributed variables were conducted using Mann-Whitney U tests. All reported *p*-values are two-tailed with statistical significance at *p*<0.05. Analyses were performed using Stata version 13.0 (StataCorp, Texas, USA) and SPSS Statistics version 22 (IBM, Portsmouth, UK).

### Ethics statement

The UK National Research Ethics Service provided ethical approval for the trial (Oxfordshire Research Ethics Committee A, 11/SC/0302), which was performed in accordance with the principles of the ICH-Good Clinical Practice guidelines and amendments. All trial participants provided written informed consent in accordance with the Declaration of Helsinki (**S1 Checklist**).

The study was registered at Clinicaltrials.gov (NCT01405521) and with the European Clinical Trials database (EudraCT 2011-000381-35).

## Results

### Study participants

**Fig 1:**
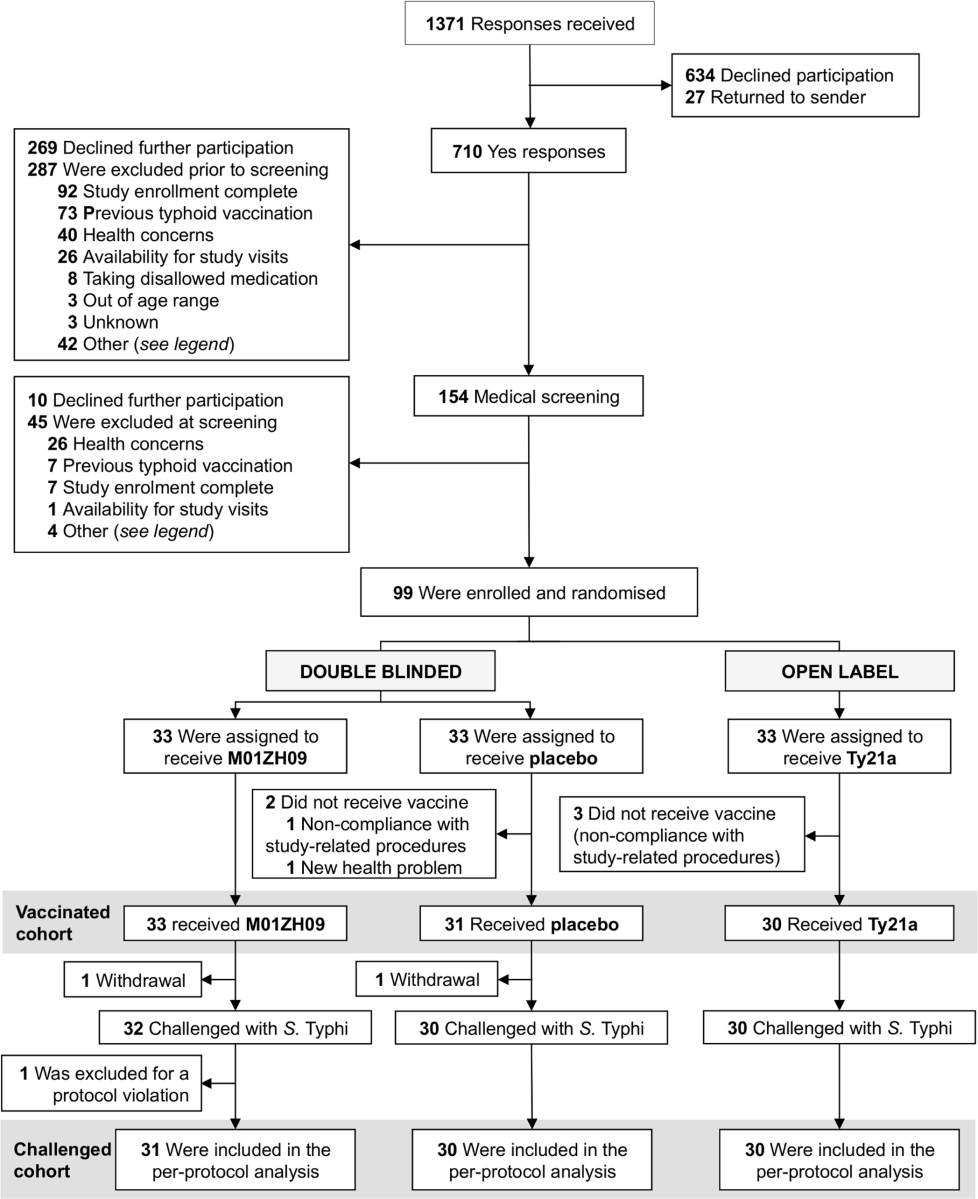
Study profile. During the screening process, participant exclusions were made either during initial telephone screening or at/after the study centre screening visit. ‘Other’ reasons for exclusions were: contact with young children (3), contact with vulnerable individuals (9), food-related occupation (5), previously resident in typhoid-endemic area for >6 months (15), unable to contact (6) & unknown (8).

Ninety-nine participants were enrolled and randomised to one of three vaccine groups between November 28, 2011, and June 27, 2012 (**Fig 1**, **S1 Dataset**). The demographic characteristics of each group were similar (**Table 1**), with an overall median (range) participant age of 30.2 (19–60) years, 64.6% male sex and 88.9% self-declared white British ethnicity. Of note, 29.6% of all participants reported previous short periods of travel (<6months duration) to areas known to be endemic for typhoid.

**Table 1:** Participant characteristics by enrolled vaccine group.

	Vaccine group		
	M01ZH09	Placebo	Ty21a
Number	33	33	33
Male sex, number (%)	22 (66.7)	19 (57.6)	23 (69.7)
Age years, median (IQR)	24 (21–43)	23 (21–39)	25 (22–31)
White British ethnicity, number (%)	29 (87.9)	31 (93.9)	28 (84.8)
**Employment status, %**			
Employed	31.3	45.5	48.4
Self-employed	6.3	3	6.5
Unemployed	12.5	6.1	0
Retired	6.3	0	0
Student	43.8	45.5	45.2
Alcohol consumption (any), %	81.8	90.9	97
Tobacco smoker (any), %	37.5	36.4	29.1
Other drug use (any), %	6.5	3	9.1
Previous travel to endemic area (<6 months duration),* %	31.2	24.2	33.3

* Note, previous travel to a typhoid endemic area >6months in duration was an exclusion criterion.

Of those randomised, 94/99 participants attended for vaccination. Compliance with fasting requirements and vaccine schedules was high (98.5%). Four weeks after vaccination 92/94 participants remained eligible and consented to challenge with *S*. Typhi. Challenge was performed a median (range) of 27 (21–33) days after vaccine course completion and the median (range) dose of *S*. Typhi bacteria ingested was 1.82 (1.46 to 2.66) x10^4^ CFU (**S1 Fig**). Ninety-one participants successfully completed the 2-week challenge period, and were included in the efficacy and outcome assessments presented below.

### Challenge outcomes and vaccine efficacy

Overall, 51/91 (56.0% [95% CI 45.2 to 66.4]) participants met the pre-specified criteria for typhoid infection during the 2-week period post-challenge. Of these, 47/51 (92.2% [95%CI 81.1 to 97.8]) diagnoses were confirmed by positive blood culture. In the placebo group, 20/30 (66.7% [95%CI 39.1 to 75.5]) were diagnosed with typhoid producing an attack rate similar to previous challenge studies (**Table 2**)[19]. After challenge, typhoid was diagnosed in 18/31 (58.1% [95%CI 39.1 to 75.5]) participants in the M01ZH09 and 13/30 (43.3% [95%CI 25.5 to 62.6]) in the Ty21a group, resulting in a calculated VE [95%CI] of 13% [-29% to 41%]) and 35% [-5% to 60%], respectively (**Table 2**).

**Table 2:** Summary of vaccine efficacy endpoints and severity measures reached during the 14-day challenge period.

	Attack rate, n (%)			Vaccine efficacy, % [95% CI]		Adjusted Vaccine efficacy, % [95% CI]	
	M01ZH09	Placebo	Ty21a	M01ZH09	Ty21a	M01ZH09	Ty21a
	(n = 31)	(n = 30)	(n = 30)				
**PRIMARY OUTCOME—TYPHOID DIAGNOSIS**							
**ALL** reaching clinical or microbiological diagnosis definition	18 (58)	20 (67)	13 (43)	13 [-29 to 41]	35 [-5 to 60]	19 [-17 to 43]	31 [-8 to 55]
Clinical definition ^A^	10	9	4	-	-		
Microbiological definition ^B^	8	11	9	-	-		
**SEVERE INFECTION**							
**ALL** reaching severe infection	3 (10)	4 (13)	1 (3)				
Oral temperature ≥40.0°C	0	1	1				
Systolic blood pressure ≤85mmHg	0	0	0				
Significant confusion or lethargy	0	0	0				
Gastrointestinal bleeding or suspected/confirmed perforation	0	0	0				
Grade 4 laboratory abnormality	3 ^C^	3 ^D^	0				
***Sensitivity Analyses of the primary outcome–varying definition of typhoid diagnosis***							
Typhoid symptoms: Typhoid triad (any fever, headache plus abdominal pain)	11 (31)	10 (30)	8 (30)				
**Fever threshold (any duration)**							
≥37.0°C	29 (94)	27 (90)	21 (70)	-4 [-21 to 11]	22 [-1 to 40]	-6 [-33 to 16]	22 [-4 to 42]
≥37.5°C	19 (61)	21 (70)	12 (40)	12 [-26 to 39]	43 [6 to 65]	21 [-12 to 44]	40 [5 to 63]
≥38.0°C	16 (52)	18 (60)	9 (30)	14 [-35 to 45]	50 [7 to 73]	19 [-27 to 48]	48 [4 to 72]
≥38.5°C	13 (42)	13 (43)	7 (23)	3 [-73 to 46]	46 [-16 to 75]	7 [-69 to 49]	44 [-21 to 74]
≥39.0°C	9 (29)	9 (30)	3 (10)	3 [-110 to 55]	67 [-11 to 90]	10 [-99 to 59]	66 [-14 to 90]
**Microbiological**							
Any S. Typhi bacteraemia	16 (52)	20 (67)	11 (37)	23 [-18 to 49]	45 [6 to 68]	28 [-7 to 52]	41 [2 to 64]
Bacteraemia or stool culture positive	21 (68)	26 (87)	16 (53)	22 [-3 to 41]	38 [12 to 57]	21 [-4 to 40]	29 [-2 to 50]

^A^ Clinical endpoint: oral temperature ≥38°C sustained for ≥12 hours or more after Day 5 of challenge.

^B^ Microbiological endpoint: blood culture positive for *S*. Typhi taken after Day 7 of challenge, or, a blood culture positive for S. Typhi plus objective symptoms of typhoid infection taken after Day 5 of challenge.

^C^ Grade 4 laboratory abnormalities M01ZH09 group: 2 hyperkalaemia (>5.6mEq/L), 1 Hypokalaemia (<3/1mEq/L).

^D^ Grade 4 laboratory abnormalities Placebo group: 1 hyperkalaemia (>5.6mEq/L), 1 hypokalaemia (<3/1mEq/L), 1 elevated liver transaminases (>10x upper limit of normal).

Adjusted vaccine efficacy estimates are adjusted for baseline Vi titre.

Overall, a similar number of participants in each group were diagnosed by either clinical (i.e. temperature ≥38°C for ≥12 hours) or blood culture criteria. This split in diagnosis type was largely due to the time required for blood culture incubation and clinician notification; the majority of participants (40/47, 85%) were bacteraemic at sampling points prior to the onset of fever (≥38°C).

Estimates of VE were sensitive to changes in the definition of TD, ranging from 3% to 23% for M01ZH09 and from 22% to 67% for Ty21a (**Table 2**). Of particular clinical interest, the finding of fever (≥38°C) with a subsequent positive, confirmatory blood culture (an approximation for passive surveillance in field-testing conditions) resulted in VEs of 52% [-25% to 81%] and 80% [16% to 95%] for either single dose M01ZH09 or 3-doses of Ty21a, respectively.

**Fig 2:**
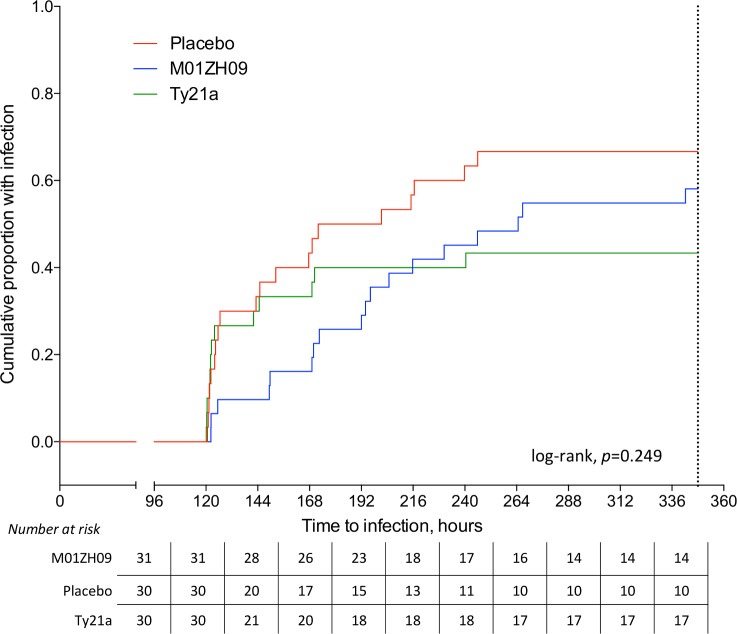
Cumulative incidence of typhoid infection after *S*. Typhi challenge at Time = 0. Time to infection, measured from challenge agent ingestion to development of first fever ≥38°C or first positive blood culture sampling. Non-diagnosed participants censored at 348 hours (dashed line). P value from log-rank test comparing all three groups.

By day 8 post-challenge, 8/31 (26%) M01ZH09 recipients and 12/30 (40%) Ty21a participants had reached the infection endpoint compared with 15/30 (50%) placebo recipients. The Kaplan-Meier median [95%CI] time elapsed between challenge ingestion to development of fever (≥38°C) or positive blood culture sampling was 265 [127 to 403] hours in M01ZH09 recipients or 172 [109 to 236] hours in placebo recipients, respectively (*p* = 0.249, log-rank; **Fig 2**).

**Fig 3:**
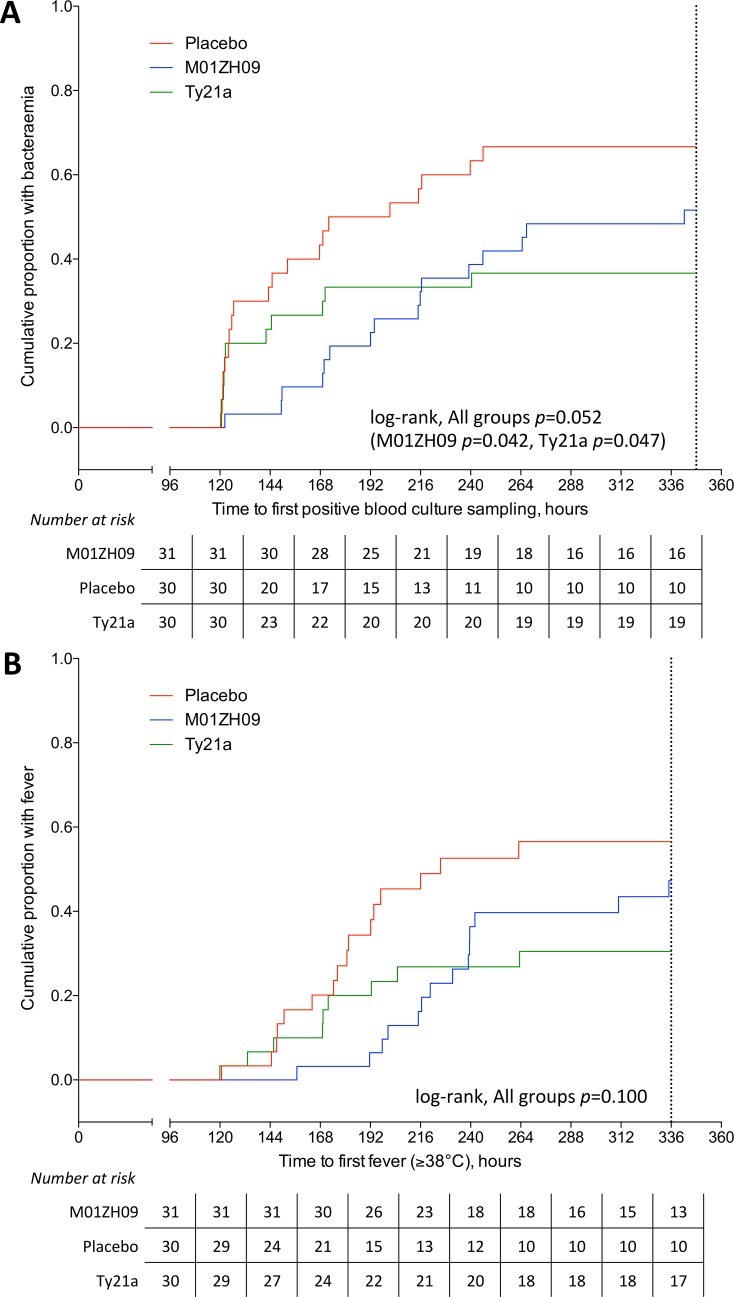
Cumulative incidence of bacteraemia or fever after *S*. Typhi challenge at Time = 0. (A) Time to bacteraemia, measured from challenge agent ingestion to time of first positive blood culture sampling. Non-bacteraemic participants censored at time of diagnosis or at 348 hours (*dashed line*). P value from log-rank test comparing all three groups and comparing M01ZH09 and Ty21a to placebo, respectively. (B) Time to fever, measured from challenge agent ingestion to first recording of fever (oral temperature ≥38°C). Afebrile participants censored at time of diagnosis or at 336 hours (*dashed line*). P value from log-rank test comparing all three groups.

Time to first positive blood culture in participants receiving M01ZH09 or Ty21a was delayed compared with placebo (*p* = 0.042 and 0.047 respectively, log-rank; **Fig 3A**). Time to onset of fever in participants was similar across groups (p = 0.1; **Fig 3B**).

Severe typhoid infection was diagnosed in 11/91 (12%) study participants, with rates of 3/31 (10%) in M01ZH09, 4/30 (13%) in placebo and 1/30 (3%) in Ty21a recipients, respectively. Most severe diagnoses (6/8) were defined by abnormalities in measured blood parameters only (see **Table 2**).

**Fig 4:**
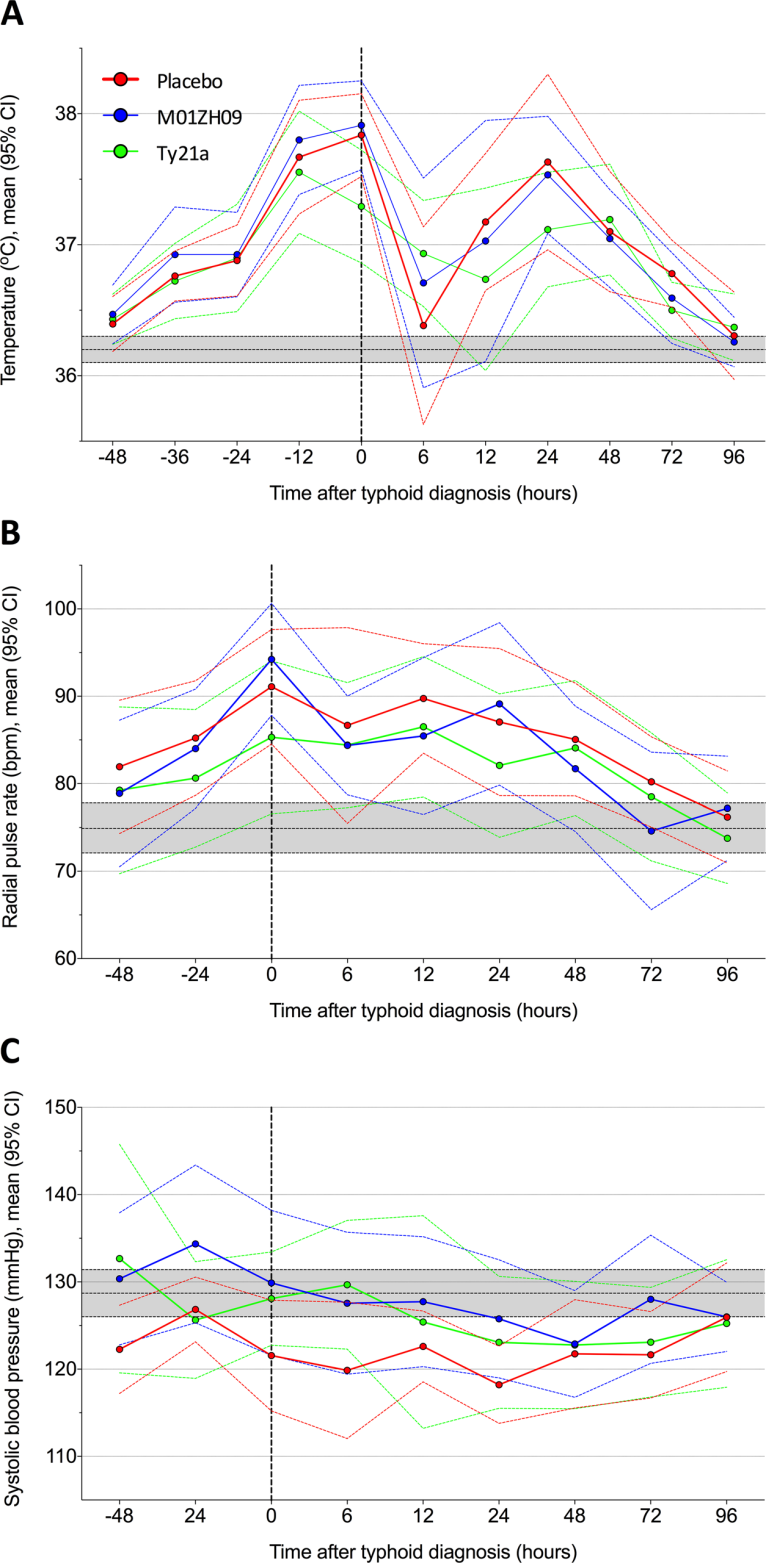
Changes in physiological signs in those participants developing typhoid by time after diagnosis according to vaccine group allocation. (A) Temperature, (B) Heart rate and (C) Systolic blood pressure. Mean change from baseline and 95% confidence interval. Dashed black vertical line marks point of typhoid diagnosis; grey horizontal bar indicates all participant mean (95% CI) values pre-vaccination.

**Fig 5:**
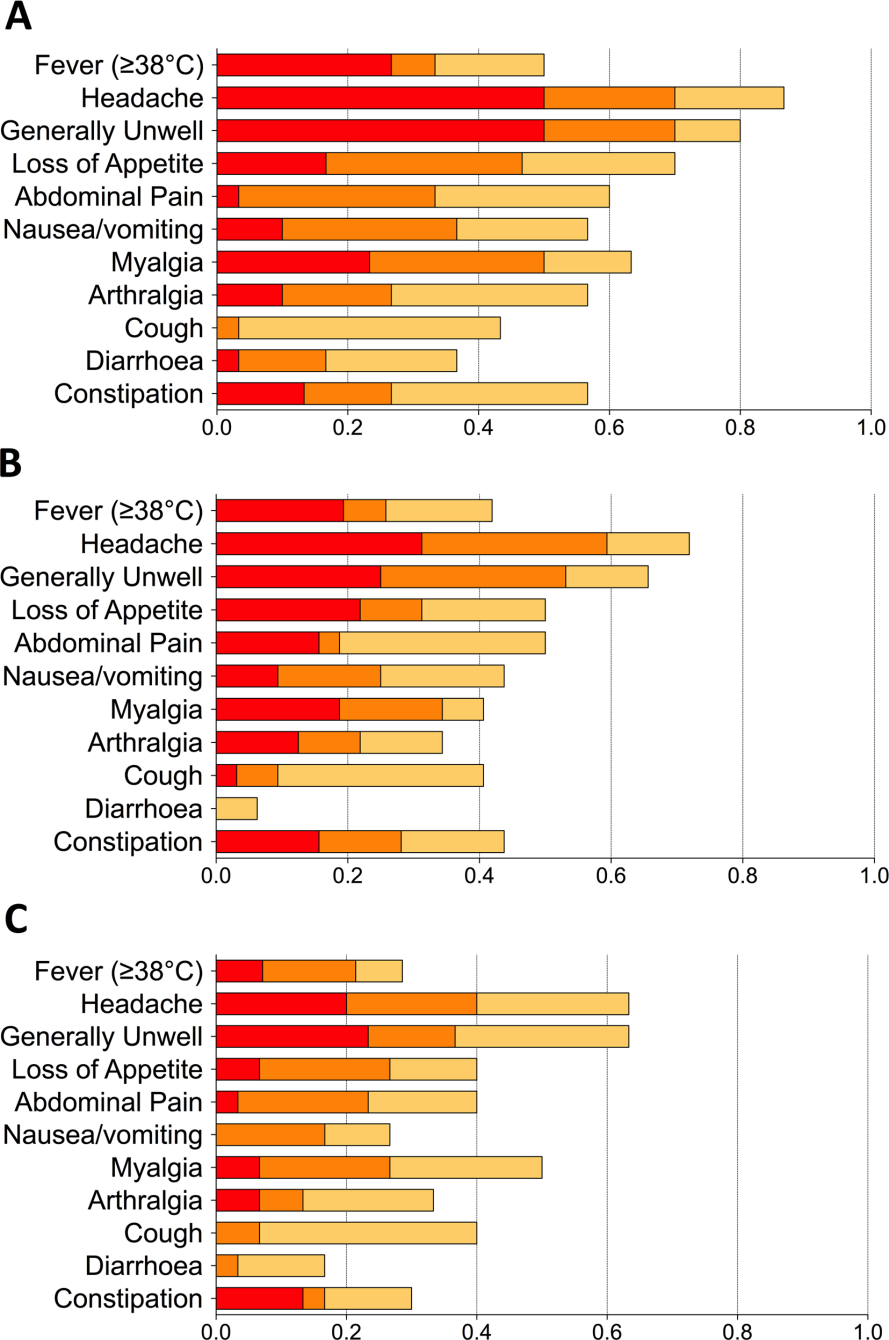
Proportion of participants reporting each solicited symptom during 14 days after challenge according to vaccine group allocation. (A) Placebo, (B) M01ZH09, and (C) Ty21a vaccine recipient groups. Maximum severity score per participant for each symptom was used and graded according to criteria detailed in the study protocol: fever thresholds are Grade 1: 38.0–38.4°C; Grade 2: 38.5–38.9°C; Grade 3: 39.0–40.0°C; Grade 4: >40.0°C.

**Fig 6:**
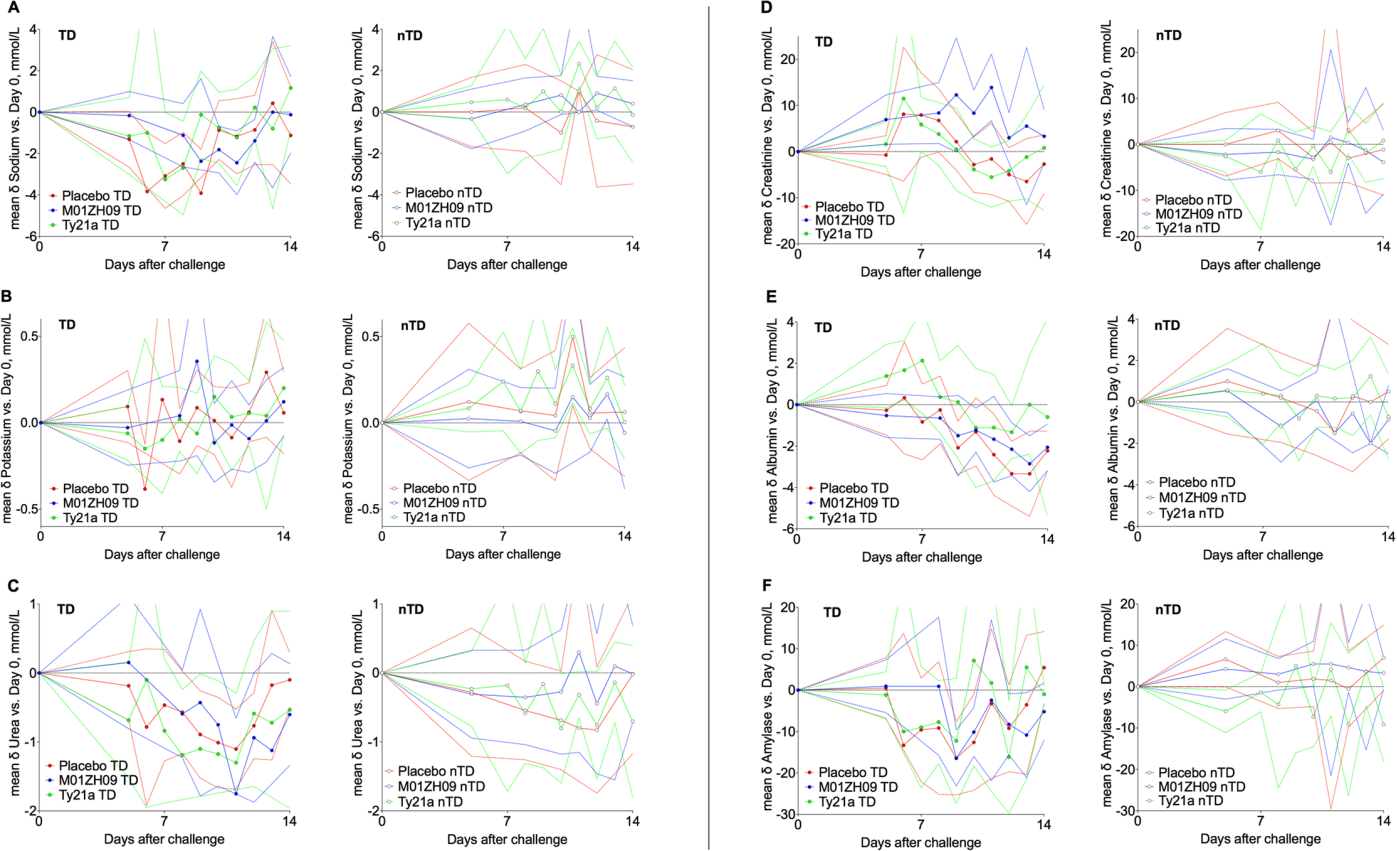
Group mean changes (95% confidence intervals) in haematological blood parameters compared to pre-challenge measurements according to vaccine allocation and challenge outcome. (A) Haemoglobin, (B) platelets, (C) total white cell count, (D) neutrophils, (E) lymphocytes, and (F) eosinophils. TD, typhoid diagnosis; nTD, non-typhoid diagnosis.

**Fig 7:**
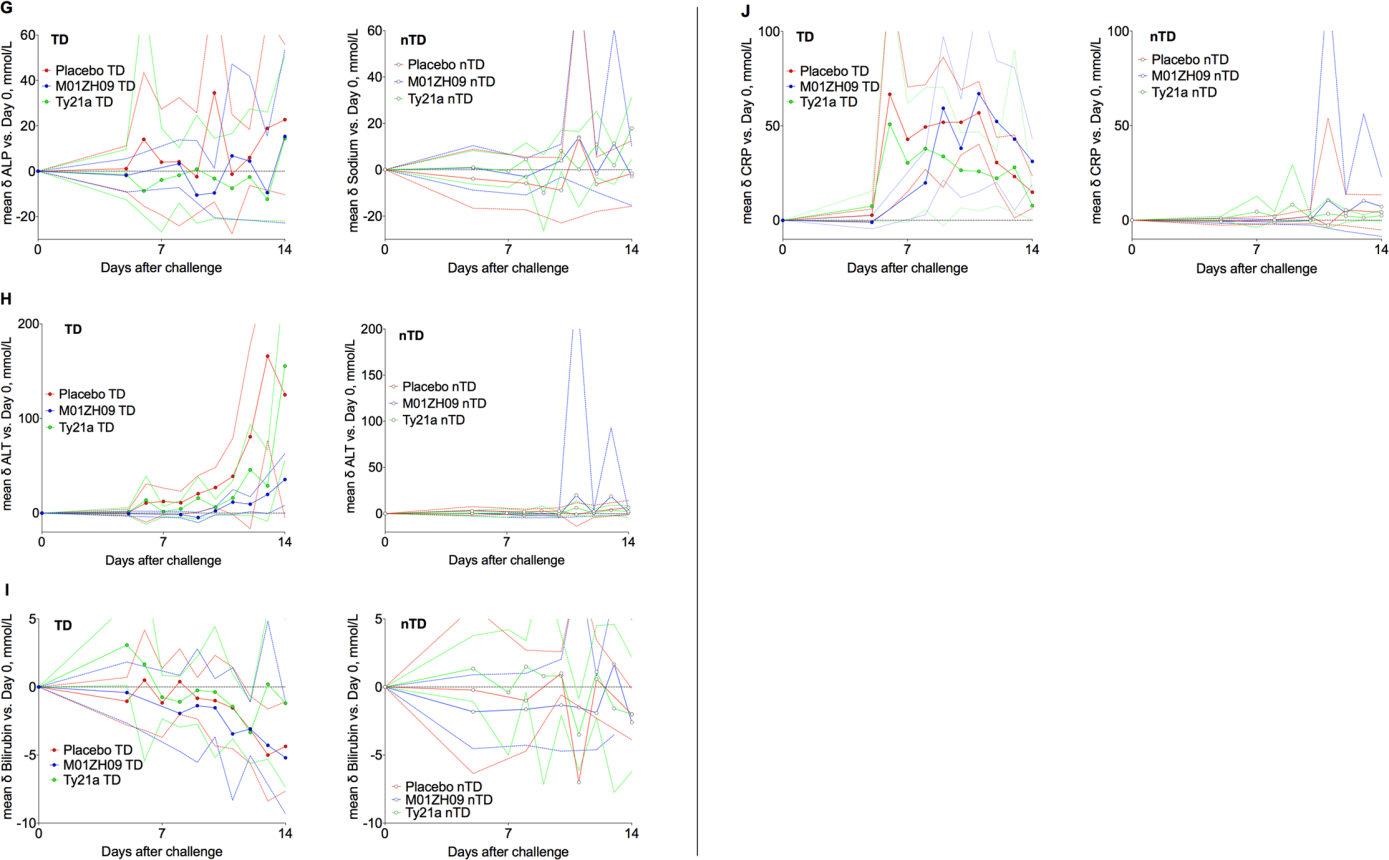
Group mean changes (95% confidence intervals) in biochemistry blood parameters compared to individual baseline measurements according to vaccine allocation and challenge outcome. (A) Sodium, (B) potassium, (C) urea, (D) creatinine, (E) Albumin, (F) amylase, (G) alkaline phosphatase, (H) alanine aminotransferase, (I) bilirubin, and (J) C-reactive protein. TD, typhoid diagnosis; nTD, non-typhoid diagnosis.

Frequent and marked physiological changes and symptoms were found in the majority of participants after challenge, particularly in those developing infection (**Fig 4**). Diary card symptoms in blinded participants showed that participants receiving M01ZH09 had fewer, milder symptoms compared to placebo (**Fig 5**, **S1 Table**). There were no clear differences in the physiological parameters of participants diagnosed with infection among the different vaccine groups (**Figs 6** and **7**).

### Microbiological outcomes

Over 1000 blood culture samples were collected from participants after challenge; due to the later clinical presentation, M01ZH09 participants tended to have a greater number of samples obtained than those in either of the other two groups (**Table 3**). The average number of positive blood cultures collected from each typhoid-diagnosed participant was similar across groups (2.25, 2.70 and 2.63 for those in M01ZH09, placebo and Ty21a groups, respectively). Of note, the median [IQR] duration of blood culture positivity (measured from time of first positive to last positive sampling) was similar between the M01ZH09 and placebo groups (28.7 [0 to 41.5] and 27.7 [21.4 to 31.3] hours, respectively) but longer in Ty21a vaccine recipients (46.3 [28.4 to 53.3] hours).

**Table 3:** Summary of microbiological results obtained over the entire study period (including stool clearance samples).

	Vaccine group, *n* (%)			
	M01ZH09	Placebo	Ty21a	ALL
***Blood cultures***				
*S*. Typhi	36 (9.7)	54 (15.6)	29 (8.7)	119 (11.3)
*Others (i*.*e*. *contaminants)*				
Coagulase negative *Staphylococci*	3	6	6	15 (1.4)
*Micrococcus* sp.	0	1	0	1
Diphtheroids	0	0	1	1
Negative	331	285	295	911 (86.8)
Missing sample	0	0	1	1
**Total sent**	**372**	**346**	**332**	**1050**
***Stool cultures***				
*S*. Typhi	47 (11.5)	53 (14.1)	49 (11.9)	149 (12.5)
Negative	362	321	361	1044
Missing sample	1	1	1	3
**Total sent**	**410**	**375**	**411**	**1196**

**Fig 8:**
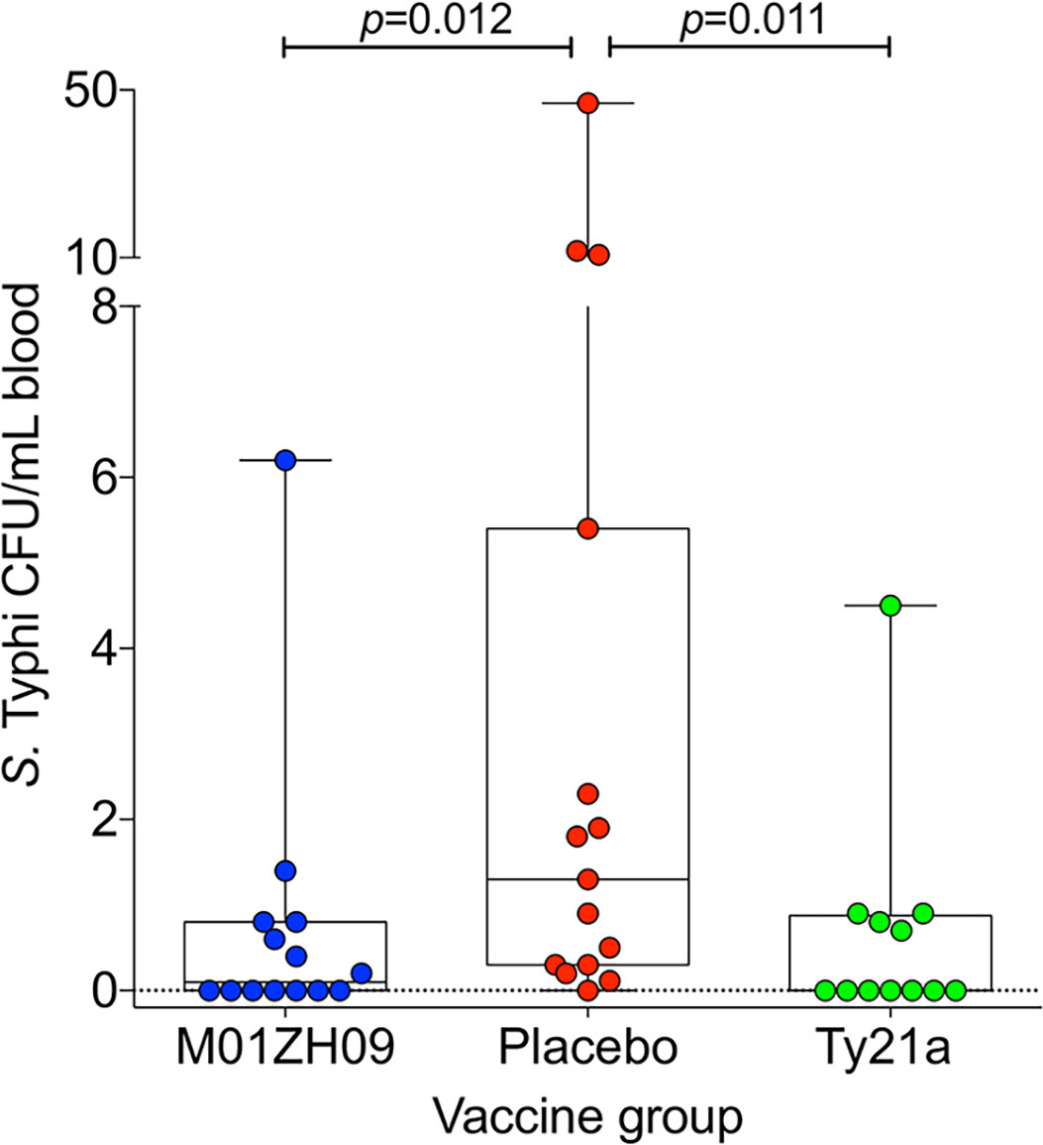
Blood quantification of Salmonella Typhi bacteria present at point of typhoid diagnosis by vaccine group. 10mL peripheral blood was collected at typhoid diagnosis (prior to antibiotic treatment) into an Isolator 10 tube (Wampole Laboratories). Lysis centrifugation was performed (30min x 3000G without brake) after which the deposit was plated to XLD and incubated aerobically at 37°C for 24 hours. After incubation, colony counts and slide agglutination tests were performed. P values calculated using Mann Whitney U test. Median [IQR] bacterial loads in CFU/mL were: M01ZH09 (n = 14): 0.13 [0.05–0.80]; Placebo (n = 15): 1.30 [0.30–5.40]; Ty21a (n = 12): 0.05 [0.05–0.99]. Lower limit of detection, 0.1 CFU/mL; zero values were substituted with LOD/2, i.e. 0.05 CFU/mL.

The quantitative *S*. Typhi load (available for 41/51 diagnosed participants) at diagnosis (prior to antibiotic initiation) was significantly lower in the blood of M01ZH09 and Ty21a recipients compared with placebo (median [IQR] bacterial load CFU/mL, M01ZH09: 0.13 [0.05–0.80], placebo: 1.30 [0.30–5.40]; *p* = 0.012; Ty21a: 0.05 [0.05–0.88]; *p* = 0.011, Mann-Whitney U test; **Fig 8**).

Early shedding of *S*. Typhi in participant stool samples (within the first 72 hours after challenge) was frequent (44/91, 49% participants) and similar between vaccine groups (M01ZH09 55%, placebo 49%, Ty21a 47% of participants; **Table 4**). Identification of early shedding was significantly associated with subsequent diagnosis of typhoid infection (relative risk [95% CI] 1.71 [1.15 to 2.53], *p* = 0.005, Chi-square test). From 72 hours after challenge onwards, *S*. Typhi was cultured from 92/790 (12%) stool samples collected from 41/91 (45%) participants. Overall, no differences in numbers of participants shedding *S*. Typhi were found in those diagnosed compared with participants who did not develop evidence of infection, either after day 4 or at any time point overall (*p* = 0.089 and p = 0.370 respectively, Chi-square test).

**Table 4:** Summary of stool shedding by participants between Day 0 (including pre-challenge) until completion of challenge, according to vaccine group allocation, challenge outcome and phase of shedding.

Group	Challenge outcome	Time point				Any time point
		Day 0 to Day 3	Day 4 onward^$^	*Day 4 to TD*	*TD onward*	
**M01ZH09**	nTD	5/13 (38)	4/13 (31)			8/13 (62)
	TD	12/18 (67)	7/18 (39)	*7/18*	*0/16*	12/18 (67)
	**ALL**	**17/31 (55)**	**11/31 (35)**			**20/31 (65)**
**Placebo**	nTD	3/10 (30)	6/10 (60)			7/10 (70)
	TD	10/19 (53)	10/20 (50)	*10/20*	*0/20*	12/20 (60)
	**ALL**	13/29^*^ **(49)**	**16/30 (53)**			**19/30 (63)**
**Ty21a**	nTD	5/17 (29)	4/17 (24)			8/17 (47)
	TD	9/13 (69)	10/13 (77)	*10/13*	*0/13*	10/13 (77)
	**ALL**	**14/30 (47)**	**14/30 (47)**			**18/30 (60)**
**ALL**	nTD	13/40 (33)	14/40 (35)			23/40 (58)
	TD	31/50 (62)	27/51 (53)			34/51 (67)
	**ALL**	**44/90 (49)**	**41/91 (45)**			**57/91 (63)**

nTD, Typhoid not diagnosed; TD, typhoid diagnosis.

*Note, one placebo recipient produced no stool samples before Day 4.

^$^ Day 4 to Day 18 (day of last sample submission from individual still having challenge follow-up visits, i.e. last TD+96 hours visit).

Antibiotic initiation rapidly terminated stool shedding, with no positive stool cultures being obtained after the first dose of treatment had been taken. No evidence of convalescent or long-term carriage of *S*. Typhi was found in any of the two follow-up stool cultures obtained.

### Vaccine safety and immunogenicity

**Fig 9:**
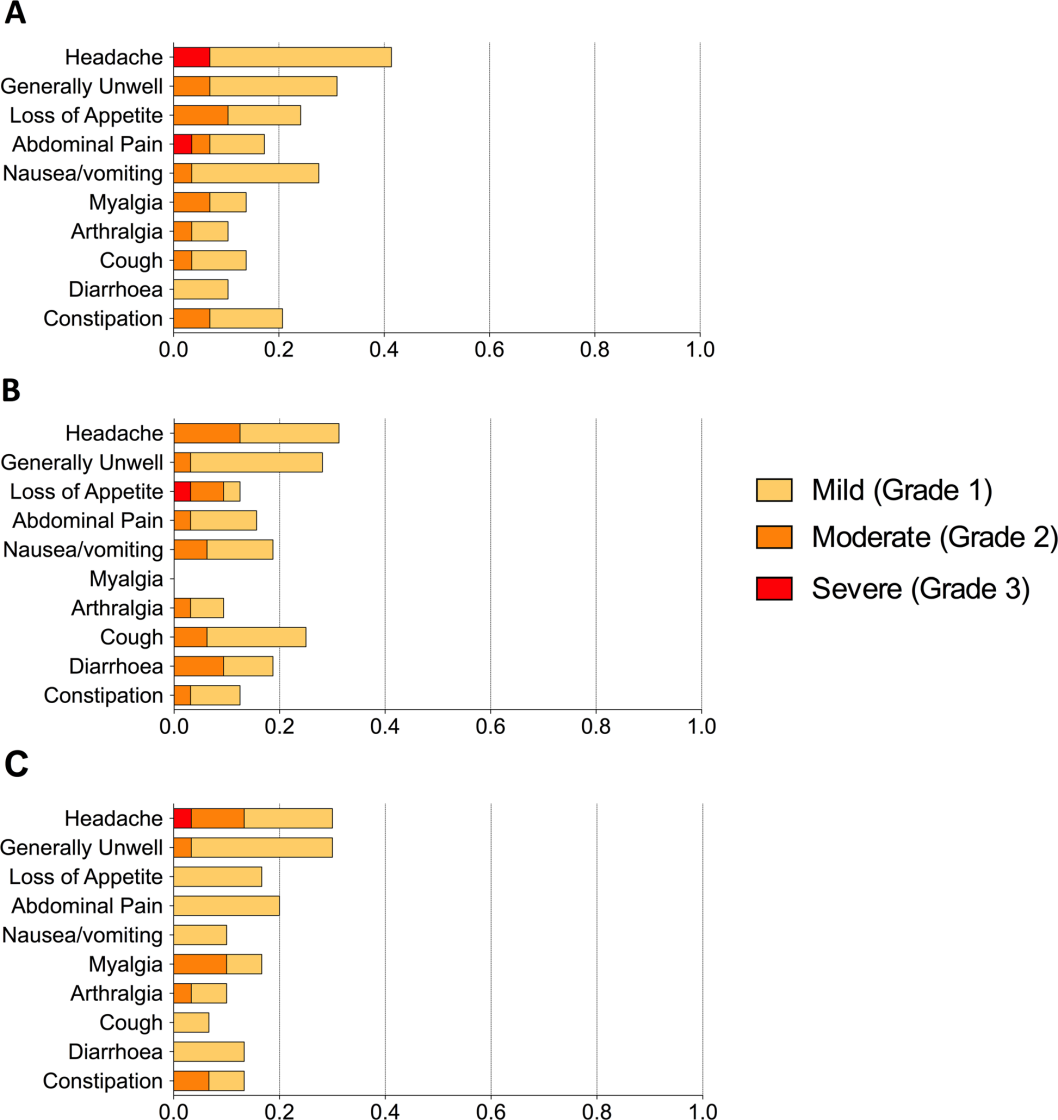
Proportion of participants reporting each solicited symptom during the 7-days after receipt of (first) vaccine dose. (A) Placebo, (B) M01ZH09, and (C) Ty21a vaccine recipient groups. Maximum severity score per participant for each symptom was used; grade 1: symptom reported but no interference with daily activity; grade 2: some interference with normal daily activities; grade 3: significant symptoms preventing normal daily activity; grade 4: potentially life-threatening (see **S1 Protocol**).

Both active vaccines and placebo were well tolerated and no Serious Adverse Events were identified related to vaccine receipt. Participants from each group reported a similar number and severity of symptoms during the 7 days after vaccine receipt (**Fig 9** and **S1 Table**).

**Fig 10:**
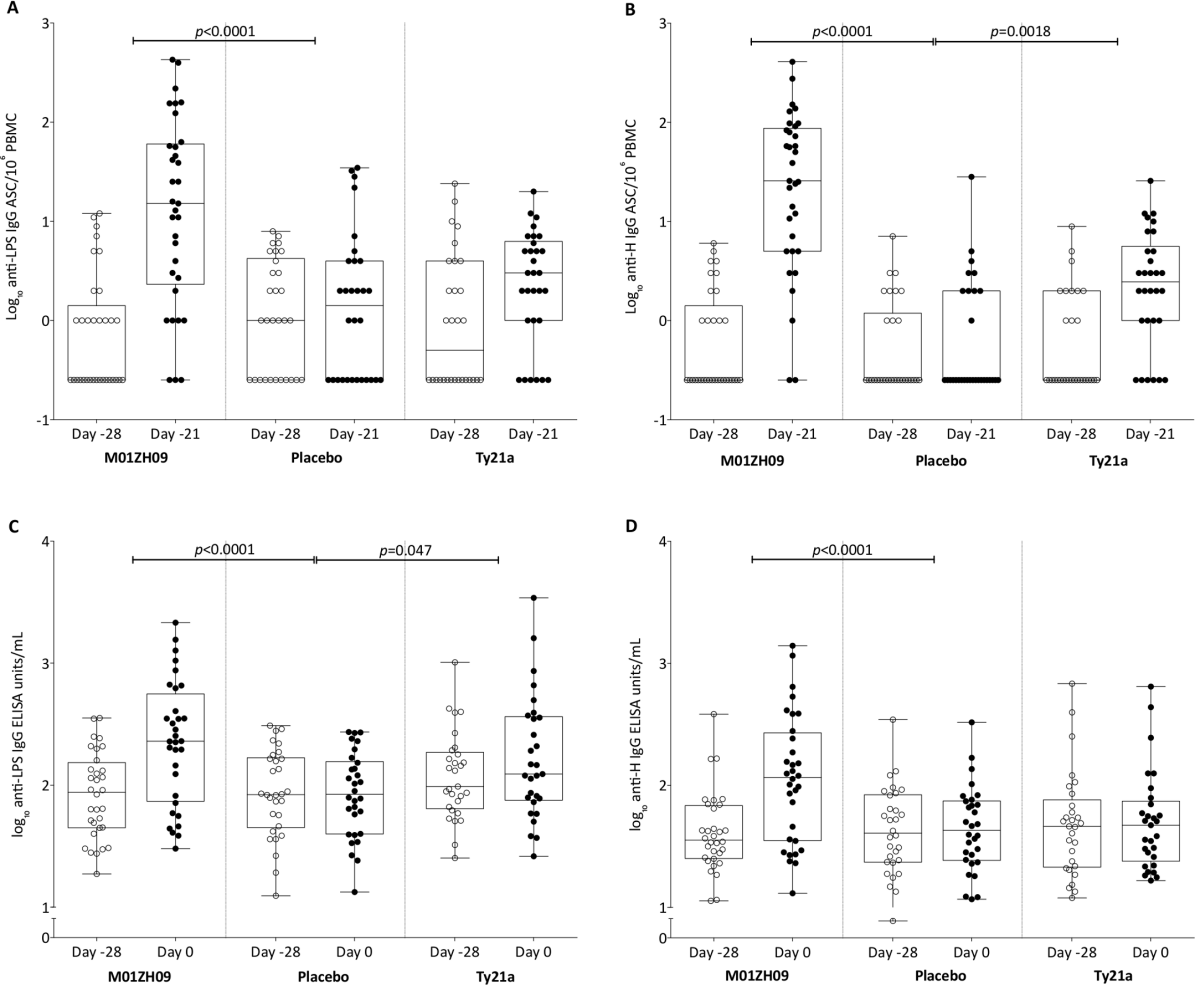
Immunoglobulin G ASC and antibody responses to *S*. Typhi LPS and flagellin before (Day -28) and after vaccination (either Day -21 or Day 0 for ASC and ELISA assays, respectively). (A) LPS and (B) flagellin specific IgG antibody secreting cell responses, respectively, measured pre-vaccination (Day -28) and 7 days later (Day -21). (C) Anti-LPS and (D) anti-flagellin antibody titres, respectively, measured pre-vaccination (Day -28) and 28 days later (Day 0, i.e. prior to *S*. Typhi challenge). Data are grouped according to vaccine allocation.

Pre-vaccination (day -28) anti-LPS, -H and -Vi ASC levels measured by ELISpot and antibody titres measured by ELISA were similar between groups (**S2 Table** and **S3 Table**). One week after a single dose of M01ZH09 or three doses of Ty21a, most participants showed a significant increase in anti-LPS and anti-H ASC isotype assays compared with placebo (**Fig 10A** and **10B**, **Table 5**). Corresponding significant increases were seen in all anti-LPS and anti-H antibody isotypes between day -28 and day 0 (prior to challenge ingestion) in response to M01ZH09 vaccine when compared with placebo (**Fig 10C and 10D**, **Table 6**). In contrast, vaccination with Ty21a resulted in a borderline significant increases in anti-LPS IgG only (*p* = 0.047, ANCOVA).

**Table 5:** Analysis of covariance comparisons for increase in ASC titres against LPS, flagellin (H) and Vi between prevaccination (Day -28) and Day -21, between each active vaccine group (1. M01ZH09 and 2. Ty21a) and placebo. Analyses were performed with the dependent variable log(change from baseline) with adjustment for log(pre-vaccination) values. GMR, geometric mean ratio compared to placebo.

Variable	Vaccine group	GMR	95% CI	P value
		(vaccine/placebo)		
LPS IgG	M01ZH09	11.2	4.7 to 26.2	< .0001
	Ty21a	1.8	0.7 to 4.3	0.189
LPS IgM	M01ZH09	74.9	37.0 to 151.6	< .0001
	Ty21a	6.2	3.0 to 12.7	< .0001
LPS IgA	M01ZH09	43.4	20.8 to 90.8	< .0001
	Ty21a	5.6	2.6 to 11.8	< .0001
H IgG	M01ZH09	35.9	16.4 to 78.3	< .0001
	Ty21a	3.6	1.6 to 8.1	0.002
H IgM	M01ZH09	119.5	63.1 to 226.4	< .0001
	Ty21a	7.6	3.9 to 14.6	< .0001
H IgA	M01ZH09	64.4	32.3 to 128.5	< .0001
	Ty21a	8.0	3.9 to 16.2	< .0001
Vi IgG	M01ZH09	0.6	0.3 to 1.2	0.1670
	Ty21a	0.7	0.4 to 1.4	0.334
Vi IgM	M01ZH09	2.3	1.1 to 4.7	0.031
	Ty21a	1.0	0.5 to 2.2	0.925
Vi IgA	M01ZH09	1.1	0.5 to 2.1	0.880
	Ty21a	1.2	0.6 to 2.5	0.586

**Table 6:** Analysis of covariance comparisons for increase in antibody titres against LPS, flagellin (H) and Vi between pre-vaccination (Day -28) and Day 0 (prior to challenge), between each active vaccine group (1. M01ZH09 and 2. Ty21a) and placebo. Analysis were performed with the dependent variable log(change from baseline) with adjustment for log(pre-vaccination) values. GMR, geometric mean ratio compared to placebo.

Variable	Vaccine Group	GMR	95% CI	P value
		(vaccine/placebo)		
LPS IgG	M01ZH09	2.7	1.9 to 4.0	< .0001
	Ty21a	1.5	1.0 to2.2	0.047
LPS IgM	M01ZH09	1.6	1.4 to 2.0	< .0001
	Ty21a	1.1	0.9 to 1.3	0.255
LPS IgA	M01ZH09	1.8	1.4 to 2.2	< .0001
	Ty21a	1.2	1.0 to 1.5	0.092
H IgG	M01ZH09	2.7	1.9 to 3.6	< .0001
	Ty21a	1.0	0.7 to 1.4	0.900
H IgM	M01ZH09	26.7	14.0 to 51.2	< .0001
	Ty21a	1.4	0.7 to 2.7	0.282
H IgA	M01ZH09	2.7	2.0 to 3.5	< .0001
	Ty21a	1.1	0.8 to 1.5	0.466
Vi IgG	M01ZH09	1.0	0.9 to 1.2	0.790
	Ty21a	1.0	0.8 to 1.1	0.593

No significant increases in anti-Vi IgG antibody levels were found in response to vaccination. Of note, a range of anti-Vi IgG antibody titres was found at baseline. These included 6/32 (19%), 12/30 (40%) and 8/29 (28%) participants in the M01ZH09, placebo and Ty21a vaccine groups respectively, with levels detectable above the lower detection limit (LLD, 7.4EU/mL) and the 75% percentile measurement found in a UK adult blood donor population sample (*n* = 81)[19].

**Fig 11:**
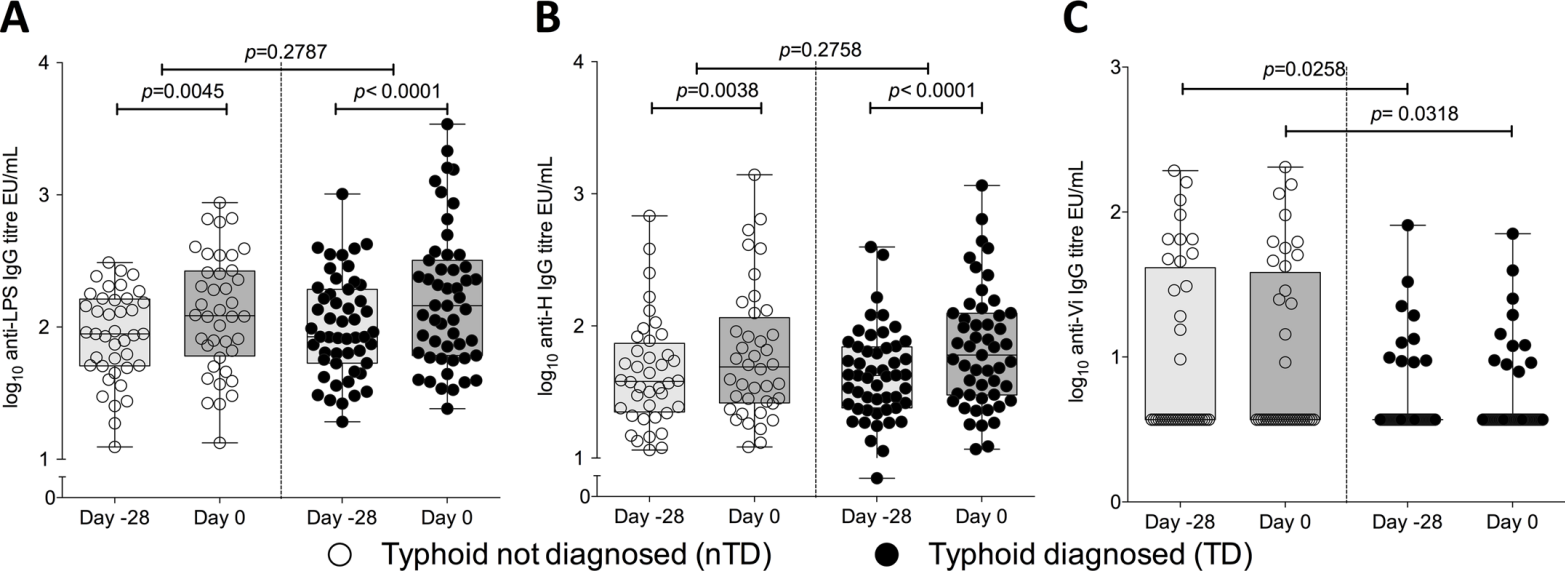
Pre-vaccination (Day -28) and pre-challenge (Day 0) IgG antibody titres according to outcome after challenge. (A) Anti-LPS IgG, (B) anti-H IgG, and (C) anti-Vi IgG. Clear circles, non-typhoid diagnosed participants; black circles, typhoid diagnosed participants. Difference between Day -28 and Day 0 anti-LPS and anti-H antibodies analysed by ANCOVA adjusted for vaccine group and baseline titre; P values for anti-Vi antibody calculated by Wilcoxon Rank Sum. Lower limit-of Vi-antibody detection, 7.4EU/mL.

Despite finding robust overall humoral anti-LPS and anti-H responses to vaccination, these responses failed to confer protection against challenge (**Fig 11A** and **11B**). While anti-Vi IgG antibody titres were unaffected by the vaccines used in this study, baseline titres were significantly higher in those subsequently found to be protected after challenge (**Fig 11C**). In an exploratory proportional hazards analysis, baseline parameters were assessed for their impact on developing typhoid during the 2-week challenge follow-up period. Anti-Vi antibody titre prior to vaccination was the only variable found to be predictive of TD. Challenge dose, fold increase in anti-LPS or anti-H antibody titres due to vaccination, sex and prior travel to endemic regions were not significant. When baseline anti-Vi antibody levels were accounted for in the model, the hazard rate of TD in the M01ZH09 group was approximately half that of the placebo group (0.513, *p* = 0.048) and, similarly, time to bacteraemia rates were 60% lower in both active vaccine groups compared with placebo (**Table 7)**.

**Table 7:** Hazard Ratios (each active vaccine group vs. placebo) and 95% confidence intervals from proportional hazards models adjusting for baseline anti-Vi IgG antibody titres.

	Predictor	HR	95% CI	*p* value
Time to typhoid diagnosis	Ty21a	0.572	0.283 to 1.157	0.120
	M01ZH09	0.513	0.264 to 0.994	0.048
	Log_10_(anti-Vi)*	0.290	0.120 to 0.700	0.006
Time to bacteraemia	Ty21a	0.428	0.204 to 0.901	0.026
	M01ZH09	0.407	0.205 to 0.810	0.011
	Log_10_(anti-Vi)*	0.272	0.106 to 0.695	0.007

* hazard ratio per 1 log_10_ increase in anti-Vi IgG titre

## Discussion

For the first time in over 40 years we have demonstrated the utility of a human challenge study in the assessment of a new typhoid vaccine candidate, M01ZH09. In phase I and IIa studies performed to-date, in low and high transmission settings and in both adults and children, a single dose of M01ZH09 has proven to be well tolerated and highly immunogenic [10–13]. In this study, we found that neither a single dose M01ZH09 nor three doses of Ty21a given 28 days prior to challenge resulted in significant overall protection. Both vaccines caused a significant reduction in the microbiological burden of infection and alteration of the clinical disease profiles, however, when compared with those participants receiving placebo only. With adjustment for baseline anti-Vi titre, M01ZH09 vaccine receipt resulted in significant protection against developing typhoid fever during the two-week period after challenge.

We successfully demonstrated the reproducibility of this model for vaccine assessment by recreating the attack rate of 67% in placebo recipients, identical to that described in our preliminary dose-escalation study [19]. The selected challenge dose (and anticipated attack rate) was chosen to avoid exposing excessive numbers of participants to a potentially infectious pathogen while also attempting to reduce the risk of overwhelming potentially protective vaccine-induced responses, as has been observed in some of the historical studies performed [26]. While we were able to document many of the features of clinical typhoid fever in our volunteers at this dose, this high attack rate in placebo recipients means that our model is likely to be a more stringent test of VE than the historical Maryland model. In these studies, challenge with 10^5^ CFU *S*. Typhi Quailes strain 5–9 weeks after vaccination with 5–8 doses of Ty21a resulted in high rates of anti-LPS antibody seroconversion and 87% protective efficacy, albeit without the constraints of a two-week follow-up period [16].

To provide some measure of the ability of our challenge model to evaluate VE, we incorporated an open label Ty21a arm using the standard European 3-dose schedule. Despite resulting in expected levels of immunogenicity [27], we found that Ty21a vaccination resulted in a protective efficacy of only 35% after challenge, a point estimate that did not reach significance. Reasons for lower efficacy compared with that found in the historic Maryland studies might include different dosing schedules and vaccine formulations used, the background immunity of study participants, challenge doses and the methods used and the availability of automated blood culture technology. It is interesting to note however, that a 35% VE corresponds to that shown for the 3-dose schedule of Ty21a at Year 1 in a recent meta-analysis including 20,543 participants [6]. Likewise, with a similar definition for TD (fever with subsequent microbiological confirmation) as was used in the original vaccine/challenge efficacy study by Gilman *et al*, Ty21a VE reached 80% [95%CI, 16 to 95] in this study compared to 87% [95% CI, 47 to 96][16].

Lower Ty21a efficacy is also likely to reflect the higher challenge dose (and thus attack rates) used in our study. Of note, the corresponding attack rate in the Maryland Ty21a studies was 53% after challenge with 10^5^ CFU in non-vaccinated volunteers [16]. During the field trials of Ty21a, VE was lowest in areas with higher infection rates and therefore probably higher exposure doses. In Indonesia, where rates of infection were 1,206/100,000 for example, VE ranged from 52.7% [95%CI. 23.9 to 58.6] in 3–19 year olds given three doses of liquid Ty21a to 23.6% [95%CI, -78.8 to 67.3] in 20–44 year olds given 3 doses of enteric-coated Ty21a during 30 months of follow-up [28]. Reasons given for this lower efficacy in the older age group included a lower number of cases in the placebo arm, possible variation in circulating *S*. Typhi strain types or vaccine production. It is interesting to note both that the same enteric-coated formulation was used in our study, and the relatively close phylogenetic relatedness of the Quailes strain to more recently found Indonesian strains [19].

In keeping with the previous studies, we found M01ZH09 to be highly immunogenic and well tolerated when given as a single oral dose. In addition to anti-LPS responses, significant increases in anti-H ASC and antibody titres were also seen in M01ZH09 recipients, in contrast to those vaccinated with Ty21a. Of note, a single dose of M01ZH09 contained approximately twice as many CFU than 3-doses of Ty21a (1x10^10^ versus 6x10^9^ CFU) although both vaccine formulations would also contain non-viable bacteria. Despite the immune responses seen, and against the expectation that high LPS antibody levels might correlate with protection against *Salmonella* infection [14, 15, 26], vaccination failed to confer significant protection against typhoid infection after challenge. Other beneficial effects of M01ZH09 vaccination were seen however, suggesting that active vaccine-mediated mechanisms were in effect. These included a noticeable delay in infection onset (effectively extending the clinical incubation period) characterised as a delay in the appearance of fever and symptoms. It is noteworthy that delay in onset of infection is used in many vaccine evaluation challenge models as evidence of VE [8]. There was also a measurable reduction in the microbiological burden of infection, both in time to bacteraemia, the level of bacteraemia at TD and some reduction in stool shedding in the few days preceding TD. While the precise immune mechanisms responsible for protection against typhoid infection are still under investigation, these data confirm that, at least in typhoid-naïve individuals, anti-LPS and anti-H responses may moderate the onset or severity of infection symptoms, but are not sufficient alone to prevent invasion and systemic dissemination.

Generation of anti-LPS antibodies is frequently used as an indicator by which to select potential oral vaccine candidates in addition to being the focus of several *Salmonella*-based vaccine programmes. Support for the protective role of anti-LPS responses comes from both field trials, in which a correlation has been found between rates of anti-LPS seroconversion and subsequent risk of typhoid infection [14], and mouse/*S*. Typhimurium models in which monoclonal antibodies confer protection against homologous challenge [29]. A central argument to using live attenuated vaccines in endemic settings is that protective immune responses may be boosted by background exposure to *S*. Typhi in food or water supplies or due to persistence of antigen in reticuloendothelial niches. This is likely responsible for the finding in field trials that VE often increases over time. The two consequences for our study relate to the very low levels of background LPS exposure, possibly resulting in suboptimal vaccine responses and thus less protection to challenge, and the short window between vaccination and challenge. It is worth noting that infants and young children are less likely to have experienced this background exposure and therefore multiple vaccine doses may be more efficacious.

Post-hoc multivariate analyses to explore factors contributing to the development of typhoid after challenge revealed the apparent protective contribution of vaccination when adjusted for baseline anti-Vi IgG antibody titre. Participants were carefully selected to be both typhoid and typhoid-vaccine naïve, by taking self-reported histories and confirming individuals’ vaccine and medical histories with their general practitioners. The finding that 29% of participants had some measurable Vi antibody detectable (albeit at low levels) at baseline was therefore unexpected. Reasons for this may include colonisation/exposure to cross-reactive or homologous antigens (*Citrobacter freundii* 5396/38) or that those with very short travel histories may have been exposed, albeit briefly and sub-clinically to *S*. Typhi whilst abroad. Notably there was no correlation found between overseas travel and detectable anti-Vi IgG. If confirmed in future planned studies, evidence of the protection afforded by even low levels of anti-Vi IgG supports current Vi polysaccharide vaccine recommendations by the World Health Organisation and prevention strategies in travellers and high-risk populations in endemic regions [7], and for the development of newer more immunogenic Vi-conjugate vaccines.

Alternatively, low-level anti-Vi responses may be a marker for other cross protective immune responses. Additional host mucosal and adaptive immune responses are likely to play a major role in the protection afforded against infection by live-attenuated vaccine strains. These include secretory IgA, classical and non-classical (HLA E-restricted) CD8^+^ cytotoxic T cells [30], mucosal associated invariant T (MAIT) cell responses [31], regulatory T cell function activation [32], and functional properties of monocyte and dendritic cells [33], and, as yet incompletely characterised alterations in the gut microbiome [34]. Maturation of several of these non-humoral responses may take longer than the 28-day period allowed between vaccination and challenge in this study [30]. Additionally, thus far we have only explored the major surface antigenic determinants (LPS, Vi and flagellin). Additional serum and secreted antibody responses (to, for example, outer membrane proteins or GroEL) are also likely to play a role in protection against infection. Likewise, functional antibody activity and antibody avidity may be important in prevention of disease [15, 35].

To provide a uniform repeatable model of typhoid challenge, non-immune, typhoid-naïve adults were specifically selected. While these findings may therefore not be directly extrapolated to endemic field settings, they are likely to be relevant to travellers venturing to those regions. The VE findings are therefore also likely to be an underestimate of efficacy in an endemic setting, due to reasons of background exposure and antigen durability.

A major advantage of performing vaccine evaluation in a closed human challenge study, is recognition of subclinical phenotypes of disease. While this allows a more accurate estimation of vaccine effect, it also makes calculation of VE more susceptible to the disease endpoint definitions used. Our study incorporated demonstrable bacteraemia in the primary endpoint definition; previous challenge studies and field efficacy studies predated the development of more sensitive automated culture techniques and used a passive surveillance system to detect illness in the community participants. We therefore likely ‘overcalled’ those with infection, resulting in an apparently higher attack rate in our highly-controlled, intensively sampled participants than may have been found in these historical studies and had the vaccine been assessed in under field conditions.

In this unique study, we demonstrate the safety and utility of an ambulant outpatient human challenge study in evaluating the efficacy of a new typhoid vaccine candidate. While M01ZH09 failed to demonstrate significant protection in the per protocol analysis, post hoc analyses provide the intriguing possibility that this single dose vaccine may provide up to 50% protection in this stringent challenge model. That low level anti-Vi antibody appears to be protective supports current efforts to develop a conjugate-Vi vaccine suitable for use in younger children, however the emergence of Vi-negative *S*. Typhi strains and *S*. Paratyphi as underscores the importance of pursuing alternative strategies, including the development of live-attenuated vaccines. If a single dose of M01ZH09 reduces the risk of infection after challenge by half and has an impact on shedding, and therefore potentially transmission, oral vaccination could be a readily delivered public health control strategy.

## Supporting Information

S1 Checklist Completed CONSORT Randomised Clinical Trial reporting checklist. (DOC)

S1 Dataset Supporting clinical trial, microbiological and immunological data. (XLSX)

S1 Protocol Understanding typhoid disease after vaccination: A single centre, randomised, double-blind, placebo-controlled study to evaluate M01ZH09 in a healthy adult challenge model, using Ty21a vaccine as a positive control. OVG2011/02
(PDF)

S1 Fig Challenge doses dispensed to 92 participants according to batch (co-challenged participants) and typhoid challenge outcome. TD, typhoid diagnosed, filled circles; nTD Typhoid not diagnosed, clear circles. Challenge dose administered was measured by direct plating from the challenge suspension onto tryptone soya agar (Oxoid) prior to colony counting after 24 hours incubation (37°C, 5%CO_2_). Challenge doses ranged from 1.46–2.66x10^4^CFU *S*. Typhi Quailes strain; median dose given was 1.82x10^4^ CFU denoted by dashed horizontal line. Scheduling of participants to receive challenge occurred at their convenience but in batches so far as possible, to enable logistic configuration of laboratory and clinical staffing. Batch size ranged from 1–10 participants (median *n* = 4, IQR 2–5); all participants within a batch were challenged on the same day and with the same challenge dose.
(PDF)

S1 Table Frequency of solicited reports of adverse events during the first 7 days after vaccine administration and during the first 21 days after challenge, according to vaccine group allocation. Severity is mean score. Symptoms in bold text represent the ‘classical triad’ of typhoid fever presentation: fever, headache and abdominal pain.
(PDF)

S2 Table Plasma antibody-secreting cell responses to vaccination with M01ZH09, placebo or Ty21a. Geometric mean concentration, GMC (95%CI). Lower limit-of-detection, 0.25 cells/10^6^PBMC. PBMC, peripheral blood mononuclear cells. GMC, measured in ASC/10^6^ peripheral blood mononuclear cells (PBMC).
(PDF)

S3 Table Anti-LPS, anti-H, anti-Vi antibody responses to vaccination with M01ZH09, placebo or Ty21a. Geometric mean titre (95%CI). Lower limit-of-antibody detection, 7.4EU/mL.
(PDF)
