# Supplementary material for: Using a Human Challenge Model of Infection to Measure Vaccine Efficacy: A Randomised, Controlled Trial Comparing the Typhoid Vaccines M01ZH09 with Placebo and Ty21a
Source: PLoS Negl Trop Dis. 2016 Aug 17;10(8):e0004926. doi: 10.1371/journal.pntd.0004926 (PMC4988630; doi:10.1371/journal.pntd.0004926)
Supplement: S1 Protocol — OVG2011/02 (PDF) [file pntd.0004926.s003.pdf]

Understanding typhoid disease after vaccination:

a single centre, randomised, double-blind, placebo-controlled study to evaluate M01ZH09 in a healthy adult challenge model, using Ty21a vaccine as a positive control.

Study reference no.: OVG 2011/02

Ethics ref.: 11/SC/0302

EudraCT no: 2011-000381-35

Date and version no.: 06.02.2013, version 5.1

**Chief Investigator:**	Professor Andrew Pollard
**Centre:**	Oxford Vaccine Group,<br>Centre for Clinical Vaccinology and Tropical Medicine,<br>Churchill Hospital,<br>Old Road, Headington, Oxford, OX3 7LJ<br>Tel/Fax. 01865 857420
**Co-investigators:**	Dr Brian Angus, University of Oxford<br>Professor Derrick Crook, University of Oxford
**Collaborators:**	Professor Gordon Dougan, Wellcome Trust Sanger Institute<br>Professor Jeremy Farrar, University of Oxford<br>Dr Paul Langford, Imperial College London<br>Professor Myron Levine, University of Maryland<br>Professor Marcelo Sztein, University of Maryland
**Sponsor:**	University of Oxford,<br>Clinical Trials and Clinical Governance<br>Joint Research Office<br>Block 60, Churchill Hospital<br>Headington, Oxford. OX3 7LJ
**Funder:**	The Wellcome Trust

Investigator Agreement

"I have read this protocol and agree to abide by all provisions set forth therein. I agree to comply with the International Conference on Harmonisation tripartite guideline on Good Clinical Practice."

Prof. Andrew Pollard

Chief Investigator Investigator Signature Date

Confidentiality Statement

This document contains confidential information that must not be disclosed to anyone other than the Sponsor, the Investigator Team, host NHS Trust(s), regulatory authorities, and members of the Research Ethics Committee.

TABLE OF CONTENTS

SYNOPSIS 11
ABBREVIATIONS 15
1. BACKGROUND AND RATIONALE 18
1.1 Pathogenesis 18
1.2 Treatment 18
1.3 Vaccines 19
1.3.1 Licensed vaccines 19
1.3.2 Vaccines in development 20
1.3.3 M01ZH09 vaccine 21
1.4 Aim of the project 22
1.5 Previous typhoid challenge studies 22
1.6 Preliminary findings from OVG 2009/10, the OVG challenge study 23
1.7 Rationale for testing M01ZH09 in a challenge model 24
1.8 The Quailes strain 25
2. OBJECTIVES AND STUDY DESIGN 27
2.1 Primary objective 27
2.2 Secondary objectives 27
2.3 Summary of study design 27
Figure 1: Flow chart summarising study plan 29
2.4 Study endpoints (including safety and tolerability endpoints) 31
2.4.1 Primary endpoint 31
2.4.2 Secondary endpoints 31
3. STUDY PARTICIPANTS 37
3.1 Overall description of study participants 37
3.2 Inclusion Criteria 37
3.3 Exclusion Criteria 38
3.3.1 Temporary exclusion criteria for vaccination visits 40
3.3.2 Temporary exclusion criteria to challenge with *S.* Typhi (Quailes strain) 41
3.4 Potential risks to participants 41
3.4.1 Venepuncture 42
3.4.2 Complications of use of the study vaccine, M01ZH09 42
3.4.3 Complications of typhoid Fever 42
3.4.4 Relapse of typhoid Fever 43
3.4.5 Chronic carrier state 43
3.4.6 Antibiotics 44
3.4.7 Pregnancy and contraception 44

Figure 2: Duration of contraception to be used during study, OVG 2011/02 44
3.4.8 Person-to-person spread of *S.* Typhi 44
3.5 Potential risks to participant contacts 45
3.6 Potential benefits 46
4. RECRUITMENT, ASSESSMENT AND RANDOMISATION 47
4.1 Recruitment and pre-screening 47
4.2 Informed Consent 48
4.3 Screening and eligibility assessment 49
4.4 Randomisation and code breaking 51
4.4.1 M01ZH09/vaccine placebo arm 51
4.4.2 Positive control arm (Ty21a vaccine) 52
4.4.3 Functional genomics subgroup 52
5. VACCINATION PROCEDURES 53
5.1 Initial vaccination visit 53
5.2 Further Ty21a vaccination visits 54
5.2.1 Recording vaccine-related side-effects 55
5.3 Additional functional genomics visits 55
5.4 Vaccination follow-up visits 55
6. *S.* TYPHI CHALLENGE PROCEDURE 57
6.1 Baseline assessment 57
6.2 Preparation of challenge agent 57
6.3 Administration of *S.* Typhi (Quailes strain) 58
6.4 Assessment after challenge 58
6.5 Assessment 12 hours after challenge 59
6.6 Subsequent assessments for all participants 59
6.6.1 Days 1 to 14 (V2-13) 59
6.6.2 Follow-up telephone calls 60
6.7 24-hour physician contact 61
6.7.1 Days 21 to 3 years (V14-V21) 61
6.7.2 Blood sampling 61
6.7.3 Obtaining participant’s weight 63
Table 2: Blood test schedule 63
7. MANAGEMENT OF PARTICIPANTS WITH TYPHOID FEVER 69
7.1 Definition of illness 69
7.1.1 Typhoid fever 69
7.1.2 Severe typhoid fever 69
7.2 Reporting to the Health Protection Unit 70

7.3 Admission to inpatient facility ... 70
7.4 Blood sampling for participants with typhoid fever ... 71
7.5 Medication ... 71
7.5.1 Antipyretics and analgesics ... 71
7.5.2 Diarrhoea ... 71
7.5.3 Constipation ... 71
7.5.4 Nausea and vomiting ... 71
7.5.5 Allergy ... 71
7.5.6 Antibiotics ... 72
7.6 Antibiotic treatment ... 72
7.7 Clearance of infection ... 73
7.8 Screening of close contacts for carriage of *S.* Typhi ... 73
7.9 Transport of samples ... 73
7.10 Blinding of laboratory samples ... 74
7.11 Bacteriology ... 74
7.11.1 Blood culture ... 74
7.11.2 Stool culture ... 74
7.11.3 Blood PCR detection ... 75
7.12 Immunology ... 75
7.12.1 Inflammatory responses ... 75
7.12.2 Antibody responses ... 76
7.12.3 Mucosal immune responses ... 76
7.12.4 Cellular immune responses ... 77
7.13 Functional genomics ... 78
7.14 Mass spectrometry ... 78
7.15 Other laboratory investigations ... 78
7.16 Participant questionnaire ... 79
8. DEFINITION OF END-OF-STUDY ... 80
9. SOURCE DATA ... 81
10. TREATMENT OF PARTICIPANTS ... 82
10.1 Description of study vaccines ... 82
10.1.1 M01ZH09 vaccine ... 82
10.1.2 Vaccine placebo (for M01ZH09) ... 82
10.1.3 Ty21a vaccine ... 83
10.2 Administration of vaccines and placebo ... 83
10.2.1 M01ZH09 and vaccine placebo ... 83
10.2.2 Ty21a vaccine ... 84

10.3 Storage of study vaccines 84
10.4 Compliance with vaccine dosing regimens 85
10.5 Accountability for the study vaccines 85
10.6 *S.* Typhi challenge strain 85
10.6.1 GMP manufacture 85
10.6.2 Storage 85
10.7 Accountability for the challenge strain 86
10.8 Concomitant Medication 86
10.9 Discontinuation/withdrawal from study at any stage 86
10.9.1 Additional safety measures for discontinuation/withdrawal after challenge 87
11. SAFETY REPORTING 88
11.1 Definitions 88
11.1.1 Adverse Event (AE) 88
11.1.2 Adverse Reaction (AR) 88
11.1.3 Serious Adverse Events 88
11.1.4 Serious Adverse Reaction (SAR) 89
11.1.5 Suspected Unexpected Serious Adverse Reaction (SUSAR) 89
11.1.6 Medically significant event 89
11.2 Reporting procedure for all Adverse Events 90
11.2.1 Vaccination-related AEs 91
11.2.2 Challenge-related AEs 91
11.2.3 Causality assessment 92
11.2.4 Severity grading criteria for adverse events 93
Table 3: Grading of solicited AE severity 95
Table 5: Severity grading of clinical examination findings 97
Table 6: Grading of unsolicited AEs 98
11.3 Reporting procedures for Serious Adverse Events 99
11.4 Procedure to be followed in the event of abnormal findings 100
11.5 Study Management Committee 100
11.6 Trial Steering Committee 100
11.7 Data and Safety Monitoring Committee 100
12. STAFF AND INVESTIGATOR SAFETY 102
13. STATISTICAL PLAN 103
13.1 Statistical hypothesis 103
13.2 Sample size and power considerations 103
Table 7. Power calculation 104
13.3 Populations for analysis 104

13.4 Analysis of demographic and baseline characteristics 106
13.5 Analysis of study endpoints 106
13.5.1 Statistical method for the primary endpoint 106
13.5.2 Statistical methods for the secondary endpoints 106
13.5.3 Clinical 107
13.5.4 Inflammatory 107
13.5.5 Microbiology 108
13.5.6 Functional genomics 108
13.5.7 Diagnostics 109
13.5.8 Immunology 109
13.5.9 Safety and tolerability 109
13.6 Level of statistical significance 109
13.7 Criteria for termination of study 110
13.8 Accounting for missing, unused or spurious data. 110
13.9 Procedure for reporting deviation from the original statistical plan 110
14. ACCESS TO SOURCE DATA AND STUDY DOCUMENTS 111
15. QUALITY CONTROL AND QUALITY ASSURANCE PROCEDURES 112
15.1 Protocol deviations 112
16. ETHICS 113
16.1 Declaration of Helsinki 113
16.2 ICH Guidelines for Good Clinical Practice 113
16.3 Approvals 113
16.4 Participant Confidentiality 113
16.5 Compensation for harm 114
17. DATA HANDLING AND RECORD KEEPING 115
17.1 Blinding of laboratory samples 116
17.2 Data integrity 116
17.3 Data archiving and storage 116
18. FINANCE AND INSURANCE 117
18.1 Insurance 117
18.2 Funding 117
18.3 Compensation 117
19. PUBLICATION POLICY 119
20. REFERENCES 120
APPENDIX 1: GMP development of a *Salmonella* Typhi challenge agent for clinical use ..125

AMENDMENT HISTORY

Amendment No.	Protocol Version No.	Date issued	Author(s) of changes	Details of Changes made
1	2.0	04.10.2011	T. Darton	1. Update of findings from OVG 2009/10.<br>2. Addition of study flowchart (figure 1).<br>3. Clarification of Pregnancy section (3.4.7) and addition of figure 2.<br>4. Alteration of randomisation method to sealed envelopes.<br>5. Removal of medical examination from visits after day 28.<br>6. Clarification of typhoid fever diagnosis in participants who are bacteraemic and symptomatic before day 5 following challenge.<br>7. Clarification of method to be used to ensure accurate IMP vaccine dose given.<br>8. Alteration of formatting and clarification of adverse event section (12.2).<br>9. Conversion of units in FDA laboratory abnormalities table (table 4).
2	3.0	11.11.2011	C. Waddington	Addition of stool samples for microbiome
3	4.0	14.12.2011	T. Darton	1. Challenge dose clarified using now available results from study OVG 2009/10, Understanding typhoid disease, Developing a *Salmonella* Typhi challenge model in healthy adults.<br>2. Section 1.6, Preliminary findings from OVG 2009/10, the OVG challenge study updated.<br>3. Correction to Section 2.1.1.1, 11.2 – post-vaccination symptoms will be recorded for 7 days following the first or only dose has been given.<br>4. Amendment to Section 4.1; individuals over age 60 may be contacted using the electoral role

				information provided, however are not eligible for study inclusion.<br>5. Clarification of Section 0 and 4.4.1 describing the process of randomisation of participants.<br>6. Addition to 4.4.3 – participants in the functional subgroup will also be asked for additional stool samples for investigation of the faecal microbiome.<br>**Correction of blood volume taken taken at typhoid diagnosis calculation in 6.7.3 Obtaining participant's height and weight**<br>To allow exploratory analysis on the effect of challenge dose per kilo of body weight, participants will be contacted by phone or email to ask them what their height and weight was at the time of challenge. Participants will also be measured and weighed at the CCVTM when attending their next available routine follow up visit if they verbally consent to do so.<br>7. Table 2: Blood test schedule (5mls more).<br>8. Correction of the e-mail address and fax number for reporting vaccine-related AEs, SAEs and SUSARs to.
4	5.0	15.08.2012	C. Waddington	2.2 and 7.16 Addition of a participant questionnaire
5	5.1	06.02.2013	R. Sewell	6.7.3 Clarification that participants height and weight will be obtained as part of study procedures

SYNOPSIS

**Sponsor**	University of Oxford	**Funding (ref. no.)**	Wellcome Trust (092661)
**Study ref. no.**	OVG 2011/02	**EudraCT no.**	2011-000381-35

**Study title**	Understanding typhoid disease after vaccination: a single centre, randomised, double-blind, placebo-controlled study to evaluate M01ZH09 in a healthy adult challenge model, using Ty21a as a positive control.
**Study start date/period**	November 2011/ participants will be followed for 37 months each
**Study duration**	4 years
**Clinical phase**	Phase II (therapeutic exploratory)
**Rationale**	It is estimated that *Salmonella enterica* serovar Typhi accounts for 21 million cases of enteric fever resulting in 200,000 to 600,000 deaths every year. Vaccination represents the most cost-effective method of controlling typhoid, although current vaccines offer limited protection to the populations most at risk. In order to profile the immunobiological response to typhoid infection and to accelerate the introduction of novel typhoid vaccines, a human challenge model has been established at the Oxford Vaccine Group.<br>M01ZH09 vaccine is an oral, live attenuated *Salmonella* Typhi vaccine based on a parent Ty2 strain containing two independently attenuating gene deletions (*S.* Typhi (Ty2 *aroC*$^-$ *ssaV*$^-$) ZH9) which has demonstrated a good safety profile and immunogenicity. In this study, the protective effect of M01ZH09 will be assessed in a human challenge model and correlates of protection will be investigated, in order that these may be used in future phase III typhoid vaccine trials.
**Study agents/ intervention descriptions**	Group 1: M01ZH09 vaccine: 1 dose, oral, 28 days pre-challenge.<br>Group 2: vaccine placebo: 1 dose, oral, 28 days pre-challenge.<br>Group 3: Ty21a vaccine (Vivotif ®): 3 doses, oral, 32, 30 and 28 days pre-challenge.<br>Challenge: 1-5x10^4 CFU of *S.* Typhi (Quailes strain) oral suspension in 30mL $NaHCO_3$ following 120mL $NaHCO_3$ oral buffer solution.

**Primary objective**	To determine the relative protective effect of M01ZH09 compared to placebo in a healthy adult typhoid challenge model using the licensed Ty21a vaccine as a positive control.
**Secondary Objectives**	1) To compare the clinical and laboratory features of the host responses following challenge with *Salmonella* Typhi (Quailes strain) in participants vaccinated with M01ZH09, placebo or Ty21A, including the time course of illness, development of bacteraemia and the inflammatory response.<br>2) To compare the host immune response following vaccination with M01ZH09, placebo or Ty21a, including innate, antibody and CMI responses and persistence of immunity and to relate these responses to the protective effect of vaccination.<br>3) To assess the safety and tolerability of M01ZH09 and to compare tolerability with Ty21a vaccine and vaccine placebo.<br>4) To develop diagnostic methods for *Salmonella* Typhi infection (including PCR and mass spectrometry-based techniques).<br>5) To explore the variation in genomic response to vaccination with M01ZH09, placebo or Ty21A and subsequent *Salmonella* Typhi challenge in participants.<br>6) To confirm the scientific integrity of the demonstrated protective effect of vaccination in the human challenge model by contemporary demonstration of the protective effect of the established Ty21a vaccine.
**Methodology**	This is a phase II single-site, double-blind randomised trial enrolling healthy adult participants in Oxford. After enrolment, participants will be randomised to receive either double-blinded M01ZH09/placebo or open-label Ty21a vaccine. These agents will be administered orally at 28 days and at 32, 30 and 28 days prior to challenge, respectively. At challenge, participants will receive a single dose of the challenge agent, *S.* Typhi (Quailes strain). At point of diagnosis (as determined by confirmation of Gram negative bacteraemia or development of a fever >38ºC for ≥12hours) participants will be treated with antibiotics to arrest infection. All participants will receive a 2-week course of antibiotics, either at typhoid diagnosis or at Day 14 post-challenge.

	All participants will be monitored throughout the study and the immunobiological profile of vaccinated participants before and after typhoid challenge will be investigated using clinical data and laboratory analysis of blood, stool, urine and saliva specimens.	
**Planned sample size**	Participants will be randomised 2:1 to receive M01ZH09/placebo or Ty21a (the positive control arm); those in the former will subsequently be randomised 1:1 to receive M01ZH09 or vaccine placebo. 33 participants will be required per group, assuming a minimum attack rate in the placebo group of 50% (and a drop-out rate of 10%) in order to demonstrate a protective effect of vaccination of 80% (1-β =90%, α=5%).	
**Study participants**	Healthy adults aged 18 to 60 years fulfilling inclusion and exclusion criteria (see sections 0, 0).	
**Vaccines**		
**1) Investigational**	Emergent BioSolutions live attenuated oral vaccine containing *Salmonella enterica* serovar Typhi Ty2 ($aroC^{-}$ $ssaV^{-}$) ZH9 (M01ZH09)	
	Dose/formulation:	$1x10^{10}$ cfu suspended in sodium bicarbonate prior to oral ingestion. Contents of glass vial(s) containing $0.2\text{-}1.7x10^{10}$ cfu in M9S basal medium (see section 0) plus 10% (w/v) sucrose reconstituted in sodium bicarbonate solution, defined volume containing $1x10^{10}$ cfu removed to administration solution.
**2) Comparator**	Emergent BioSolutions M01ZH09 vaccine-placebo	
	Dose/formulation:	M9S basal medium plus 10% (w/v) sucrose reconstituted as above
**3) Positive control (licensed)**	Crucell live attenuated oral vaccine containing *Salmonella enterica* serovar Typhi Ty21a (Vivotif®)	
	Dose/formulation:	not less than 2×10^{9} viable cells per enteric-coated capsule, taken on day 1, 3 and 5 by oral ingestion
**Challenge agent**	*Salmonella enterica* serovar Typhi (Quailes strain)	
	Dose/formulation:	$1\text{-}5x10^{4}$ CFU suspended in sodium bicarbonate prior to oral ingestion

**Primary endpoint**	The proportion of participants developing typhoid fever after challenge with 1-5x10^4 CFU of *Salmonella* Typhi (Quailes strain) in a sodium bicarbonate buffer given 28 days after M01ZH09 vaccine in comparison to placebo.
**Secondary endpoints**	Post-vaccination and post-challenge clinical characteristics and immunobiological responses will be measured for participants in each group as described in sections 5, 6 and 0. Comparisons will be made as described in section 13 and the (separate) Statistical Analysis Plan.
**DSMC**	Prof. David Lalloo (Chair), Liverpool School of Tropical Medicine<br>Dr. David Hill, National Travel Health Network and Centre<br>Dr. Philip Monk, Health Protection Agency, East Midlands South<br>Prof. Andrew Nunn, MRC Clinical Trials Unit

ABBREVIATIONS

AE	Adverse event
ALT	Alanine transaminase
AR	Adverse reaction
ASC	Antibody secreting cell
AST	Aspartate transaminase
BD	bis in die (Latin: twice a day; prescription medicines)
CI	Chief investigator or confidence interval
CCVTM	Clinical Centre for Vaccinology and Tropical Medicine
cfu	Colony forming unit
CMI	Cell-mediated immunity
CRF	Case Report Form
CRO	Contract Research Organisation
CRP	C-reactive protein
CT	Clinical Trials
CTA	Clinical Trials Authorisation
CTL	Cytotoxic T-lymphocyte
CTRG	Clinical Trials & Research Governance, University of Oxford
DSMC	Data Safety and Monitoring Committee
EDTA	Ethylenediamine tetraacetic acid
ELISA	Enzyme linked immunosorbent assay
ELISPOT	Enzyme linked immunosorbent spot assay
ESR	Erythrocyte sedimentation rate
GCP	Good Clinical Practice
GP	General Practitioner
HADS	Hospital Anxiety and Depression Scale
HPA	Health Protection Agency
(TV) HPU	(Thames Valley) Health Protection Unit
IB	Investigators Brochure
ICF	Informed Consent Form
ICH	International Conference of Harmonisation

IMP	Investigational medicinal product
IRB	Independent Review Board
ISF	Investigator site file
LFT	Liver function tests
LLN	Lower limit of normal
LPS	Lipopolysaccharide
MAX	Maximum (prescription medication)
MHRA	Medicines and Healthcare products Regulatory Agency
NRES	National Research Ethics Service
ORH	Oxford Radcliffe Hospitals
OVG	Oxford Vaccine Group
OXTREC	Oxford Tropical Research Ethics Committee
PBMC	Peripheral blood mononuclear cell
PCR	Polymerase chain reaction
PIL	Participant/ Patient information leaflet
PO	Per oral (by mouth)
PR	Per rectum (by rectum)
PRN	pro re nata (latin: as required; prescription medicines)
QDS	quater die sumendus (Latin: four times a day; prescription medicines)
R&D	NHS Trust Research & Development Department
REC	Research Ethics Committee
RPM	Revolutions per minute
RRT	Renal replacement therapy
SAE	Serious adverse event
SAR	Serious adverse reaction
SBA	Serum bactericidal assay
SMP(C)	Summary of Medicinal Product (Characteristics)
SOP	Standard Operating Procedure
SPI	Salmonella pathogenicity island
SUSAR	Suspected unexpected serious adverse reactions
TMF	Trial Master File

TOPS	The Over volunteering Prevention System (see: http://www.tops.org.uk)
TSB	Tryptone soya broth
TSC	Trial Steering Committee
TSG	Oxford Radcliffe Hospitals Trust / University of Oxford Trials Safety Group
ULN	Upper limit of normal
V[*n*]	Visit [number]
Vi	Virulence antigen
ViPS	Virulence antigen polysaccharide (vaccine)
WBC	White blood cell/count
XLD	Xylose lysine deoxycholate

1. BACKGROUND AND RATIONALE

Salmonella enterica serovar Typhi causes an estimated 21 million new cases of enteric fever and 216,000 deaths every year.[1] Typhoid fever is transmitted faecal-orally through the ingestion of water and food vehicles contaminated with *S.* Typhi. It is found most commonly in developing countries where infrastructural facilities including provision of clean drinking water and sewage disposal facilities are inadequate. Most parts of South Asia, South-east Asia, Central Asia, Africa, and South America are considered endemic for this disease with an annual incidence of >100 per 100,000 population.[2] Although conventionally thought to be a disease of school-aged children and young adults, there is increasing evidence of high rates of infection in those under 5 years of age in these areas.[3-5] In developed countries typhoid fever also remains an important health consideration for travellers visiting endemic areas and laboratory workers.[6-8] The increasing prevalence of antibiotic resistance amongst clinical isolates compounds the problems presented by *Salmonella* Typhi.[9-16] With a high burden of disease and the rise of multi-resistant strains, it is recognised that vaccines represent the most cost-effective approach in controlling typhoid infection.[17,18] Recent evidence also suggests that there is an increasing burden of related *Salmonella* Paratyphi and non–typhoidal salmonellosis in South-east Asia in particular, for which current diagnostic tests and vaccines are even less suited.[19-21]

1.1 Pathogenesis

After ingestion and transit to the small intestine *Salmonella* Typhi adheres to epithelial M (microfold) cells overlying Peyer's patches, where mucosal invasion occurs mediated by a type III secretion system (T3SS) which is encoded by SPI-1 (see below) . The organism is taken up by macrophages by bacterial-mediated macropinocytosis or phagocytosis where intracellular survival, and thus establishment of systemic infection, is mediated by SPI-2. Following drainage to local lymph nodes, sub-clinical bacteraemia disseminates the organism further around the reticulo-endothelial system (to the liver, spleen and bone marrow) within 24-hours of ingestion. One to two weeks later there is a second, more sustained bacteraemia that accompanies the onset of fever and other constitutional symptoms (headache, malaise, lethargy, and abdominal pain). In the 2nd week, hepato-splenomegaly and rose spots may appear. In the untreated or inadequately treated patient, intestinal haemorrhage and perforation may occur after the 3rd week of illness resulting from hyperplasia, ulceration and necrosis of the Peyer's patches at the site of *S.* Typhi invasion.

1.2 Treatment

Fluoroquinolone and cephalosporin antibiotics, chloramphenicol and azithromycin may be used to effectively treat *Salmonella* Typhi infection.[22-24] Antimicrobial resistance is

increasingly reported from regions with high rates of enteric fever, however; a recent survey of 3000 isolates in Nepal found 5% resistance to ciprofloxacin, 14% to amoxycillin, 12% to cotrimoxazole and 13% to chloramphenicol.[25]

Vaccines

Licensed vaccines

There are three vaccines currently licensed for the prevention of typhoid fever by active immunisation. These are:

1) The inactivated whole-cell vaccine, which is immunogenic in all age groups but also highly reactogenic, making it unpopular for widespread use as a control measure. It is virtually no longer used.[26]

2) The virulence factor (Vi) capsular polysaccharide (ViPS) vaccine was developed in the 1980s, and offers the possibility of typhoid control at the population level.[27-30] As a Thymus independent type-2 antigen, however, ViPS does not generate immunological memory and its effect is not boosted by repeated vaccination.[31-33] In common with other polysaccharide vaccines, it is also non-immunogenic in children under 2 years of age, presumably owing to the absence of a splenic marginal zone in early childhood necessary for generation of the anti-polysaccharide immune response. ViPS vaccine is, at best, only moderately efficacious with protective efficacy demonstrated to be 72, 64 and 69% in field trials in Nepal, South Africa and China, respectively.[28,34,35] Its duration of efficacy is also very limited, with protection lasting 2 to 3 years only.[36,37]

3) As a live attenuated oral vaccine, Ty21a stimulates local mucosal immunity within the gut as well as systemic cell-mediated immunity and antibody responses following oral administration.[38,39] There are extensive safety data available for Ty21a demonstrating excellent tolerability; it is the only currently licensed oral vaccine for the prevention of typhoid fever. Limitations include the multiple dosages required for full immunogenicity and efficacy to occur. A three dose, alternate day regimen is recommended in all countries except the USA and Canada, where a four dose schedule is recommended for travellers.[40] Furthermore, Ty21a is not licensed for use in children below 6 years of age, principally because the only formulation currently available, enteric coated capsules containing lyophilized vaccine, is not readily amenable for use in toddlers and pre-school children. In contrast, a “liquid” formulation of the vaccine (reconstitution of lyophilised vaccine buffer and water) has been shown to be practical, well tolerated and immunogenic as an oral vaccine cocktail.[41] Similar to ViPS vaccine, the Ty21a vaccine is only moderately efficacious.

Protective efficacy for the live oral vaccine, as obtained from a trial in 109,000 schoolchildren in Santiago, Chile, is reported to be 67% over three years following three doses of enteric-coated preparation given on alternate days.[42] An efficacy of 62% was observed after 7 years follow-up. Three doses of Ty21a (spaced one week apart) in enteric-coated capsules conferred 42% vaccine efficacy over 30 months in Indonesia.[3] Three doses of a “liquid” formulation in the Indonesian field trial gave 53% protection,[3] while the same formulation given as three doses spaced 48 hours apart conferred 77% protection over 3 years in a field trial in Chile.[43]

It can be seen that currently available vaccines are limited in their efficacy, cannot be used in young children and do not easily fit into the World Health Organisation’s Extended Programme of Immunization (WHO EPI).[44] Novel vaccines with improved efficacy that can be given as a single dose to all age groups, including young children, are needed to reduce the morbidity and mortality seen with typhoid fever.

S. Typhi is a human restricted pathogen with no known environmental reservoir, thus elimination of typhoid fever by effective control measures including vaccination is a possible achievement.

Vaccines in development

In order to improve typhoid vaccine efficacy and to make an effective vaccine suitable for use in young children, a ViPS-recombinant *Pseudomonas aeruginosa* exotoxin A protein conjugate vaccine (Vi-rEPA) was developed by John Robbins and colleagues at the NIH in 1994.[45] Such glycoconjugate vaccines are T-cell dependent antigens and produce immunological memory that can be boosted and might be expected to provide protection in young children. Indeed, two doses of the Vi-rEPA conjugate vaccine administered 6 weeks apart was highly immunogenic and had a protective efficacy of 91.1% in children aged 2–5 years in Vietnam over 27 months follow-up.[46] Moreover, no serious adverse effects were reported from the 5525 children who received two doses of the Vi-rEPA vaccine; minor adverse reactions included temperature of 37.5°C or over in 1.35% of participants after the first dose and swelling of at least 5 cm (which resolved within 48 h) in 0.36% of participants following the second dose. A recent study has also confirmed its efficacy in newborn infants and its compatibility with the EPI schedule in Vietnam.[47] It is hoped that by conjugation, a new generation of polysaccharide-conjugates may offer a greater degree of protection than previous vaccines, especially in young children.[48,49] There has been delay in this particular vaccines licensure however, due to lack of regulatory precedent for rEPA carrier protein-based vaccines. Diphtheria toxin-based Vi conjugates, using *Citrobacter freundii* derived Vi, are currently being investigated as possible alternatives.[50-52]

Oral vaccination confers additional advantages in inducing mucosal immunity at the site of the body that first encounters the pathogen, and those based on live attenuated organisms may also lead to enhanced and broader immune responses.[38,53] By avoiding use of needles, vaccines delivered by the oral route also offer advantages in ease of administration, increased age-dependent and cultural acceptability and may also be associated with fewer systemic side effects.[53-55] A new generation of live oral vaccines designed to be more immunogenic than Ty21a is in various stages of clinical testing. These include the Δ*aroC*, Δ*aroD*, Δ*htrA* strain CVD908-*htrA*;[56,57] strain χ4073 with mutations in *cya*, *crp*, *cdt*; strain Ty800 with a mutation in *phoP*/*phoQ*; strain CVD909, an Δ*aroC*, Δ*aroD*, Δ*htrA* strain that constitutively expresses Vi;[58] and the Δ*aroC*, Δ*ssaV* strain ZH9, which forms the basis of the M01ZH09 vaccine.[59]

M01ZH09 vaccine

The M01ZH09 vaccine is a live attenuated *Salmonella* Typhi vaccine based on the parent Ty2 strain containing two independently attenuating gene deletions. Mutation of the *aroC* gene prevents synthesis of aromatic amino acids required for bacterial growth. A second mutation in the *ssaV* gene causes structural abnormality in the specialised type III secretion system encoded by SPI-2 (Salmonella pathogenicity island-2).[60] Based on work performed with *S.* Typhimurium in mice, it is thought that absence of this secretion system causes the inability of *S.* Typhi to survive within macrophages.[61] As intracellular survival is important for the systemic spread of *Salmonella* Typhi, mutation of *ssaV* is also thought to prevent systemic spread of the bacteria.[62] In trials of this vaccine to date, systemic spread of the vaccine strain has yet to be observed.[63,64]

The safety and immunogenicity of M01ZH09 has been studied in six trials in the US, UK and Vietnam. The vaccine is given as a single dose and has been well tolerated at all tested doses with gastrointestinal side effects being the most commonly reported adverse event.[64] Almost all adverse events have been of mild severity and short in duration. Immunogenicity studies have shown both IgA and IgG responses to the lipopolysaccharide (LPS) of the vaccine.[63,65] IgG and IgA responses to LPS are generally accepted as correlates of protection against typhoid infection, although their precise contribution is undefined.[66]

Recently, M01ZH09 has also demonstrated immunogenicity and acceptability when used in children aged 5 to 14 years during field trials in Vietnam.[65] Phase III trials of M01ZH09 are now required to move this vaccine forwards to licensure. In the absence of definite correlates of protection, however, phase III trials would need to be large and of sufficient duration to demonstrate a significant reduction in the local incidence of typhoid fever. This makes these trials prohibitively costly.

In this trial, we will use M01ZH09 to investigate correlates of protection in a bacterial challenge model in order that these may be used in future phase III trials. The degree of protection afforded by the vaccine in the challenge model will also demonstrate the potential efficacy of M01ZH09 in an immunologically naive population, which would map directly onto the likely degree of protection afforded by this vaccine to travellers from developed regions.

The efficacy of the licensed Ty21a vaccine was demonstrated in the 1960-70s during previous human challenge studies performed in Maryland.[67] Ty21a vaccine will be used in this trial as a positive control to confirm whether its previously demonstrated protective effect may be replicated in the OVG challenge model.

Aim of the project

The aim of this project is to accelerate the introduction of typhoid vaccines into populations with a high burden of disease. Currently vaccine efficacy against typhoid fever cannot be predicted as correlates of protection against typhoid are unknown. Hence implementation of vaccine programmes in disease endemic regions currently requires large and expensive phase III trials in each new population, significantly delaying programme implementation. This project aims to use the established model of infection with *Salmonella* Typhi in healthy adult participants to demonstrate the protective effect of the novel oral attenuated M01ZH09 vaccine. If the vaccine confers significant protection, it is hoped that by combining the efficacy data and detailed immune response data correlates of protection can be identified. This will help accelerate the development and assessment of novel vaccine candidates.

Previous typhoid challenge studies

Challenge studies involving almost 2000 participants were conducted in the 1960s/70s at the University of Maryland. This work described the generation of humoral immunity to *Salmonella* Typhi and demonstrated the utility of a live challenge study in accelerating the development of novel vaccines.[68,69] However, because, at that time, techniques to measure cell-mediated immunity (CMI) were rudimentary, limited CMI data were obtained. In the typhoid challenge model used at the University of Maryland during the 1960s and 1970s, wild-type *Salmonella* Typhi (Quailes strain) was given to immunised and non-immunised control participants in 45 ml of skimmed milk; no additional buffer was given. The usual challenge inoculum used in vaccine efficacy studies was 10^5 CFU, which resulted in clinical attack rates of 30-55% among the controls.[68,70,71] In earlier dose-response studies with this strain, 0/14 participants experienced clinical illness when 10^3 *Salmonella* Typhi were given, whereas a high attack rate was achieved when 10^7 CFU were ingested.[68] In the early challenge models, once criteria for illness were met, therapy was instituted with chloramphenicol, the antibiotic of choice at that time. However, while all ill participants

responded to the course of chloramphenicol, approximately 0-15% of participants subsequently experienced clinical relapses (generally a much milder illness), that began days or weeks after the course of chloramphenicol was completed. Moreover, chloramphenicol had little effect on excretion of *Salmonella* Typhi. The fluoroquionlone ciprofloxacin is an important antibiotic treatment for acute typhoid fever as well as for the treatment of chronic biliary carriers.[72,73] Relapses almost never occur after a 14-day course of ciprofloxacin and excretion of *Salmonella* Typhi is rapidly curtailed within a few days of initiating therapy. Due to the exquisite sensitivity of *Salmonella* Typhi to ciprofloxacin and its concentration in bile, ciprofloxacin has also been very useful in the eradication of chronic typhoid carriage.[74] Before ciprofloxacin, the definitive treatment of chronic gall-bladder carriage involved cholecystectomy followed by a month of amoxicillin therapy. By contrast, four weeks of ciprofloxacin is 90% efficacious, without surgery, even when gallstones are present.[74] Great care has been taken in the development of this model to select participants with a low risk of becoming chronic carriers of *Salmonella* Typhi, in particular by the planned exclusion of anyone with gallstones or gall bladder disease identified on ultrasound scanning.

A model of infection in healthy adult volunteers has been established at the Oxford Vaccine Group, University of Oxford based on the original challenge experiments.[75] This Oxford Vaccine Group protocol used the same typhoid strain used in the previous challenge model in Maryland but with the major modification of using sodium bicarbonate to neutralize gastric acid of the participants immediately before administering the pathogenic *Salmonella* Typhi. This modification served to reduce the inoculum size required to cause clinical illness, and produce a more homogeneous clinical response. The same typhoid strain used in the previous Maryland studies and in the development of the OVG challenge model will be used in this study – the Quailes strain.

Preliminary findings from OVG 2009/10, the OVG challenge study

A total of 41 healthy adult volunteers were successfully challenged with *Salmonella* Typhi (Quailes strain) between February and October 2011 in a dose escalation study.

21 volunteers were challenged at the initial dose of 1-5x10^3 CFU. Of these, 20 were included in the per protocol analysis, in whom an attack rate of **55%** was seen. In the 11 volunteers fulfilling the criteria for typhoid diagnosis, 6 reached both the microbiological and clinical endpoint, 3 reached only the microbiological endpoint and 2 reached only the clinical endpoint.[75] *S.* Typhi has been isolated in stool samples from 11 participants, both in those subsequently diagnosed with typhoid infection (n=9) and those remaining well (2). In general, all participants have tolerated infection well; the most predominant symptom of fever settling between 48 and 72 hours following diagnosis of infection. Other symptoms reported include

headache, constipation and myalgia. Total reported symptom duration has lasted a median of 4 days (range 2-7 days).

As the attack rate desired was not achieved, using the dosing algorithm given in protocol OVG 2009/10, the challenge dose was subsequently increased to 1-5x10^4 CFU. A further 20 participants were successfully challenged using this dose – an overall attack rate of 65% (13/20) was seen. At the higher dose participants tended to develop infection sooner (median 6 days vs. 8.5 days) and, in general, remained symptomatic for a longer duration. Symptoms continued to remain mostly mild or moderate in severity. No study participants required admission to the John Warin (Infections Diseases) ward, however, several participants were observed in the participant rest area out-of-hours. 3 participants required a home visit as they felt too unwell to attend the outpatient clinic; none required subsequent admission. Of the 20 participants challenged at the higher dose, 2 developed severe typhoid based on a fever >40ºC. One participant experienced severe diarrhoea for 48 hours after the initiation of ciprofloxacin antibiotics. Their symptoms settled after initiation of second line antibiotic treatment (azithromycin), therefore the symptoms were thought to be antibiotic rather than typhoid related. All participants completed a course of antibiotics that cleared the infection (as defined by negative stool clearance samples 4 weeks following the antibiotic course). There were no cases of secondary transmission reported.

Rationale for testing M01ZH09 in a challenge model

The challenge model will be used to investigate the protective effect of the novel oral M01ZH09 vaccine in immunologically naive volunteers. It is hoped that by combining the efficacy data from the vaccine with immune response data the correlates of protection for typhoid fever with this vaccine can be identified. This study will provide opportunities to further progress our understanding of typhoid fever and the response to oral vaccines. These include:

1) The opportunity to study the course of the disease and how it is modified by prior vaccination with the novel M01ZH09 or the established Ty21a vaccine. In particular, it will allow the study of both the inflammatory response and development of bacteraemia following acute infection using molecular methods, enabling us to study typhoid infection without using fulminant disease as a diagnostic endpoint.

2) The opportunity to fully describe the early host immune responses including the innate response to vaccination, subsequent development of CMI immunity and persistence of antibody, using modern laboratory techniques.

3) The identification of "correlates of protection" against *Salmonella* Typhi infection (and carriage), which will provide future vaccine studies/clinical trials with potential

immunogenicity endpoints. This would have a major effect in accelerating vaccine development and implementation, and in providing a means for bridging efficacy trial data into new populations using immunological correlates of protection rather than undertaking further expensive efficacy trials.

4) Providing data to support the value of large-scale field trials in endemic areas. Randomized, placebo-controlled, double-blind field trials in endemic areas are expensive, and results are often not available for several years. Data from this model could support earlier implementation of typhoid vaccine initiatives.

5) Providing data on the protective effect of the vaccine in persons from non-endemic areas, such as travellers from industrialised countries.[42,76] Populations participating in field trials in endemic areas may have repeated contact with wild-type *Salmonella* Typhi, either prior or subsequent to study-vaccination. Consequently many participants have been immunologically primed, artificially increasing a vaccines immunological effectiveness; or, the vaccine-derived immunity of vaccinees may be boosted by subsequent contact with wild-type infection. Travellers from industrialized countries lack antecedent immunologic priming and are not subject to repetitive boosting. Therefore, the challenge model will provide useful information as to whether the candidate vaccine can protect immunologically naive participants. It is worth noting that attenuated strain Ty21a, the only currently licensed live oral typhoid vaccine, was shown to be protective in participant challenge studies in the early 1970s prior to demonstrating effectiveness in field trials in endemic areas.[70,76,77]

6) The study will provide further safety and tolerability data for the M01ZH09 vaccine.

7) Establishing methodologies applicable to other related diseases, including (but not limited to) *Salmonella* Paratyphi and non–typhoidal salmonellosis, facilitating further understanding of related infection/immunological processes.

8) Attenuated *Salmonella enterica* serovar Typhi strains have been proposed as live oral vectors for delivery of vaccine antigens. Oral vaccination is easier to administer and more acceptable to recipients, and the ability of *Salmonella* Typhi to elicit a wide ranging immune response raises the theoretical possibility that oral serovar Typhi live vector vaccines may serve as an alternative to certain parenteral vaccines currently in use. Information from this study will help further this approach.[78,79]

The Quailes strain

Salmonella Typhi (Quailes strain) was used extensively for human challenge studies in the 1960s/70s and has been provided by the University of Maryland to establish a master cell bank in Oxford. Full antibiotic sensitivity of the strain in the master cell bank has been

demonstrated and further characterization work including genome-sequencing has been completed at the Sanger Institute, Cambridge UK.

Prior to the development of the challenge model, study investigators met with the UK regulator (MHRA) and established the regulatory framework for challenge studies and received advice that the Quailes strain is not an Investigation Medicinal Product. Use of the Quailes strain therefore, does not require assessment or approval by the MHRA. Details of the GMP manufacturing process and testing of batches of the Quailes strain are described in Appendix 1.

2. OBJECTIVES AND STUDY DESIGN

Primary objective

To determine the relative protective effect of M01ZH09 vaccine compared to placebo in a healthy adult typhoid challenge model, using the licensed Ty21a vaccine as a positive control.

Secondary objectives

1) To compare the clinical and laboratory features of the host responses following challenge with *Salmonella* Typhi (Quailes strain) in participants vaccinated with M01ZH09, placebo or Ty21A, including the time course of illness, development of bacteraemia and the inflammatory response.
2) To compare the host immune response following vaccination with M01ZH09, placebo or Ty21a, including innate, antibody and CMI responses and persistence of immunity and to relate these responses to the protective effect of vaccination during subsequent challenge.
3) To assess the safety and tolerability of M01ZH09 and to compare tolerability with Ty21a vaccine and vaccine placebo.
4) To develop diagnostic methods for *Salmonella* Typhi infection (including PCR and mass spectrometry-based techniques).
5) To explore the variation in genomic response to vaccination with M01ZH09, placebo and Ty21A, and subsequent *Salmonella* Typhi challenge in participants.
6) To confirm the scientific integrity of the demonstrated protective effect of vaccination in the human challenge model, by contemporary demonstration of the protective effect of the established Ty21a vaccine.
7) To gather information on participant experiences of being in the study.

Summary of study design

This is a single centre, randomised, double-blind, placebo-controlled study of the live attenuated oral vaccine candidate, M01ZH09, in a healthy volunteer challenge model of typhoid infection. The established Ty21a vaccine will be given to a further cohort of participants in the same study to demonstrate the integrity of the model in measuring the protective effect of vaccination (a positive control).

Participants will be randomised in a 2:1 ratio to receive either M01ZH09 or placebo (66 participants), or the positive control (Ty21a vaccine) (33 participants). Within the vaccine and

placebo cohort, further randomised allocation to receive M01ZH09 or placebo will be effected through blinded packaging containing either active vaccine or vaccine placebo in a 1:1 ratio. M01ZH09 and placebo will be given as a single-dose regimen 28 days prior to challenge (Day -28). Open-label Ty21a vaccine will be given as 3 doses 48-hours apart on Days –32, -30 and -28.

Four weeks after completion of the immunisation or placebo course, participants will be challenged with *Salmonella* Typhi (Quailes strain) at an infective dose (1-5x10^4 CFU) previously demonstrated to give the desired clinical/laboratory attack rate.[75] A summary of the overall trial can be seen in **Figure 1 and Table 1** below. The day of challenge is referred to as the zero time point (Day 0) to maintain consistency with the previous protocol 'Understanding typhoid disease: developing a *Salmonella* Typhi challenge model in healthy adults' (OVG 2009/10)[75] to aid in the comparison of findings from the two studies. Hence, vaccination and post-vaccination (Va and Vb) visits occur at days prefixed with a minus sign, e.g., Va, the first post-vaccination visit, occurs at day -21 which is 21 days/3 weeks prior to challenge.

Figure 1: Flow chart summarising study plan

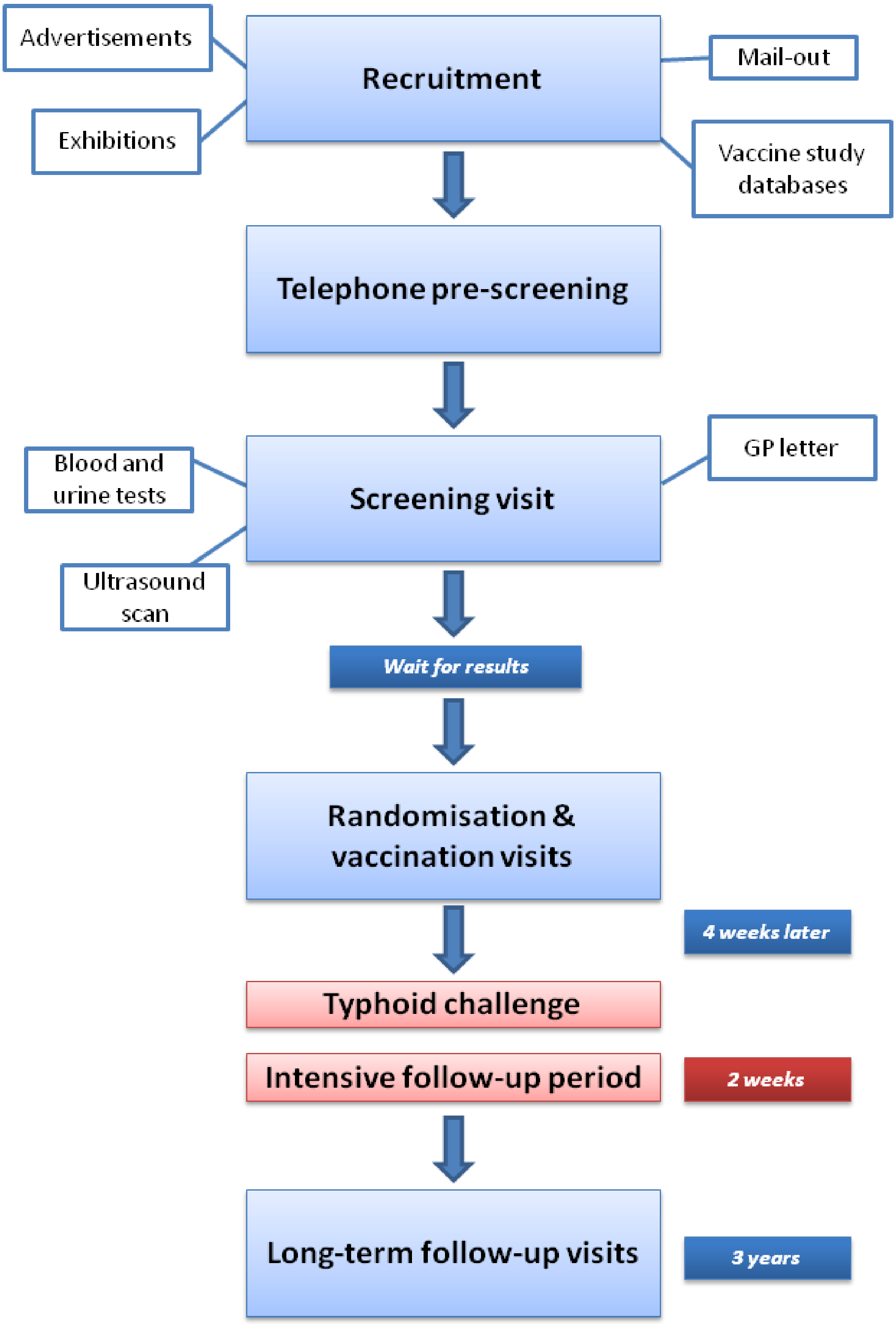

Table 1: Summary of study procedures

	Screening	Vaccinations (days -32, -30 and -28 for Ty21a, day -28 for M01ZH09 or placebo)	Post vaccination follow-up, Va (Day -21)	Post vaccination follow-up, Vb (Day -14)	Day 0 (Visit 1)	Days 1 -14 (Visits 2-13)	On diagnosis of typhoid fever (TD) or at day 14	Days 21, 28, 60, 90, 180 (visits 14- 18)	1 year, 2 years and 3 years post-challenge (visits 19- 21)
Consent	X	X €							
Medical exam	X	X			X	X	X		
Blood sample †	X	X	X	X	X	X	X	X	X
Urine sample	X				X	X	X	X	X
Saliva sample		X	X	X	X	X	X	X	
Pregnancy test *	X	X			X		X ‡		
Stool sample		X Ω	X Ω	X Ω	X	X	X	X ¥	X
Abdominal ultrasound	X								
ECG	X								
Psychological assessment	X	X			X	Day 7 and 14			
Vaccination(s)		X							
S. Typhi Challenge					X				
Diary card review			X			X	X	X	
Antibiotic treatment							X		
Telephone call/text					X $	X $	X #		

€ Informed continued consent; * for females of child bearing potential; ‡ prior to antibiotics; † refer to **Table 2** (section 0); Ω coproantibodies and faecal microbiome only; ¥ plus screening for chronic carriage/clearance; #until completion of antibiotic treatment; $ twice daily, unless visit scheduled.

Study endpoints (including safety and tolerability endpoints)

Primary endpoint

The proportion of participants who develop typhoid fever after challenge with 1-5x10^4 CFU of *Salmonella* Typhi (Quailes strain) in sodium bicarbonate buffer given 28 days after M01ZH09 vaccine in comparison to placebo.

Secondary endpoints

2.1.1.1 Post-vaccination symptoms

From administration of vaccine (first or only dose, as applicable) and for the next 7 days, the number and proportion of participants experiencing the following symptoms in each vaccine group will be compared:

- Malaise
- Headache
- Myalgia/ Arthralgia
- Anorexia/ Loss of appetite
- Nausea/ Vomiting
- Flatulence
- Constipation/ Diarrhoea
- Abdominal pain
- Cough
- Rash
- Fever (oral temperature)

Symptom severity, graded as none, mild, moderate or severe, will also be compared.

2.1.1.2 Post-vaccination immune responses

The following responses will be measured in all participants following vaccination and prior to challenge. Exploratory, descriptive analyses will be made by vaccine group, as specified in section 0.[i]

- Geometric mean IgG, IgM and IgA antibody concentrations to *Salmonella* Typhi O, H and Vi antigens at screening, Va (day -21) and Vb (day -14).
- Mean fold-rise in IgG, IgM and IgA antibody concentrations to *Salmonella* Typhi O, H and Vi antigens from screening to Va (day -21) and Vb (day -14).
- Proportion of participants demonstrating a 4-fold or greater rise in IgG, IgM and IgA antibody concentrations to *Salmonella* Typhi O, H and Vi antigens between screening and day 0 (i.e. 28 days after completion of vaccine course).

---

[i] Note: 'Baseline' refers to sample taken at the first vaccination visit, prior to vaccination. Other vaccination response timings refer to time post completion of vaccine course.

- Geometric mean S*almonella* Typhi specific serum bactericidal antibody (SBA) titres at baseline, Va (day -21) and Vb (day -14).
- Geometric mean number of cells per 10^6 PBMCs, secreting IgG and IgA antibody in response to *Salmonella* Typhi O, H and Vi antigens at baseline and Va (day -21).
- Proportion of participants demonstrating a greater than 8-fold increase in the number of cells per 10^6 PBMCs secreting IgG and IgA antibody in response to *Salmonella* Typhi O, H and Vi antigens between baseline and Va (day -21).
- Mean proportion of specific memory T cell subsets (including T effector/memory, T_{EM}; T central/memory, T_{CM}; CD45RA$^+$ T_{EM}, T_{EMRA}) able to secrete IFN-γ, TNF-α and/or IL-2 as measured by colour flow-cytometry at baseline and Vb (day -14).
- Measurements of cytotoxic T cell activity (CTL) against *Salmonella* Typhi-infected targets at baseline and Vb (day -14). These assays will include **(1)** autologous targets for classical class-Ia-restricted CTL and **(2)** the 721.221.AEH cell line for class-Ib HLA-E-restricted CTL. Results will be expressed as percentage specific cytotoxicity (in chromium release or similar non-radioisotope cytotoxicity assays) or as the percentage of cells expressing CD107 and/or intracellular perforin or granzyme B (measured by flow-cytometry).
- Mean serum concentration of pro-inflammatory cytokines (including, but not limited to IL-1β, IL-2, IL-4, IL-5, IL-6, IL-8, IL-10, IL-12, TNF-α and IFN-γ) at baseline, Va (day -21) and Vb (day -14).
- Mean concentration of cytokines in PBMC culture supernatants produced in response to soluble *Salmonella* Typhi antigens (e.g., flagella, OmpC, GroEL) and *Salmonella* Typhi-infected targets measured by ELISA or multiplex assays at baseline and Vb (day -14).
- Mean proportion of cells secreting cytokines in response to soluble *Salmonella* Typhi antigens (e.g., flagella, OmpC, GroEL) and *Salmonella* Typhi-infected targets as measured by flow-cytometry and/or ELISPOT (expressed as mean spot counts per 10^6 PBMCs) at baseline and Vb (day -14).
- The relative abundance (ratio to baseline) of gene expression (measured by gene expression microarrays and/or mRNA-seq technologies) at baseline, Va (day-21) and Vb (day -14); and at the additional four time points (FG-1, FG-2, FG-3 and FG-4) for those in the functional genomics subgroup.
- Geometric mean salivary IgA antibody concentration to *Salmonella* Typhi O, H and Vi antigens at baseline and days Va (day -21) and Vb (day -14).

- Geometric mean concentration of total faecal IgG and IgA and specific IgG and IgA (including IgA1 and IgA2 subclass determination) antibodies against *Salmonella* Typhi LPS O, H and Vi antigen in stool samples at baseline and Va (day -21) and Vb (day -14).

2.1.1.3 **Post-challenge symptoms**

After challenge with 1-5x10^4 CFU of *Salmonella* Typhi (Quailes strain) until completion of the antibiotic course, the number and proportion of participants experiencing the following symptoms in each vaccine group will be compared:

- Malaise
- Headache
- Myalgia/ Arthralgia
- Anorexia/ Loss of appetite
- Nausea/ Vomiting
- Flatulence
- Constipation/ Diarrhoea
- Abdominal pain
- Cough
- Rash
- Fever (oral temperature)

Symptom severity, graded as none, mild, moderate or severe, will also be compared.

Additionally, for participants given a diagnosis of typhoid fever (see section 0), the following descriptive analyses will also be made by vaccine group:

- Number and proportion of participants with *Salmonella* Typhi bacteraemia (as detected by blood culture).
- Number and proportion of participants with severe typhoid infection (as defined in section 0).
- Number and proportion of participants with a diagnosis of *Salmonella* Typhi infection based on blood PCR-based assays measured at 12 and 24 hours post-challenge and on days 2 to 14.
- Number and proportion of participants excreting *Salmonella* Typhi (Quailes strain) in stool (as detected by stool culture) on days 1 to 14, 21 and 28 post-challenge.

2.1.1.4 **Post-challenge immune responses**

The following responses will be measured in all participants and exploratory, descriptive analyses will be made by vaccine group and/or attainment of typhoid fever diagnosis:

- Mean CRP level on days 0, 5, 8, 10, 12, and 14.
- Geometric mean IgG, IgM and IgA antibody concentrations to *Salmonella* Typhi O, H and Vi antigens at days 0, 1, 5, 7, 10, 14, 28, 60, 90, 180 and years 1, 2 and 3.

- Mean fold-rise and proportion of participants achieving a 4-fold or greater rise in IgG, IgM and IgA antibody concentrations to *Salmonella* Typhi O, H and Vi antigens between day 0 and subsequent time points.
- Geometric mean IgG, IgM and IgA antibody concentrations to *Salmonella* Typhi LPS, membrane preparation and whole cell in lymphocyte supernatant, at days 0, 7, 10, 14 and 28.
- Geometric mean S*almonella* Typhi specific serum bactericidal antibody (SBA) titres at days 0 1, 5, 10, 14, 28, 60, 90, 180 and years 1, 2 and 3.
- Geometric mean number of cells per 10^6 PBMCs, secreting IgG and IgA antibody in response to *Salmonella* Typhi O, H and Vi antigens at day 7.
- Proportion of participants demonstrating a greater than 8-fold increase in the number of cells per 10^6 PBMCs secreting IgG and IgA antibody in response to *Salmonella* Typhi O, H and Vi antigens between baseline and day 7.
- Mean proportion of specific memory T cell subsets (including T effector/memory, T_{EM}; T central/memory, T_{CM}; CD45RA$^+$ T_{EM}, T_{EMRA}) able to secrete IFN-γ, TNF-α and/or IL-2 as measured by colour flow-cytometry at days 0, 7, 10, 14, 21, 28, 60, 90, 180 and years 1, 2 and 3.
- Mean frequency of specific memory B cells to *Salmonella* Typhi antigens (including LPS and flagella) measured by ELISPOT on expanded PBMC at days 0, 7, 10, 14, 21, 28, 60, 90, 180 and years 1, 2 and 3.
- Measurements of cytotoxic T cell activity (CTL) against *Salmonella* Typhi-infected targets at days 0 and 180 and years 1, 2 and 3. These assays will include **(1)** autologous targets for classical class-Ia-restricted CTL and **(2)** the 721.221.AEH cell line for class-Ib HLA-E-restricted CTL. These results will be expressed as percentage specific cytotoxicity (in chromium release or similar non-radioisotope cytotoxicity assays) or as the percentage of cells expressing CD107 and/or intracellular perforin or granzyme B (measured by flow-cytometry).
- Mean serum concentration of pro-inflammatory cytokines (including, but not limited to IL-1β, IL-2, IL-4, IL-5, IL-6, IL-8, IL-10, IL-12, TNF-α and IFN-γ) at day 0 (hour 0), day 0 (hour 12) and days 7, 10, 14, 21, 28, 60, 90, 180, and years 1, 2 and 3.
- Mean concentration of cytokines in PBMC culture supernatants produced in response to soluble *Salmonella* Typhi antigens (e.g., flagella, OmpC, GroEL) and *Salmonella* Typhi-infected targets measured by ELISA or multiplex assays at day 0 and days 7, 10, 14, 21, 28, 60, 90, 180 and years 1, 2 and 3.

- Mean proportion of cells secreting cytokines in response to soluble *Salmonella* Typhi antigens (e.g., flagella, OmpC, GroEL) and *Salmonella* Typhi-infected targets measured by flow-cytometry and/or ELISPOT (expressed as mean spot counts per 10^6 PBMCs) at day 0 and days 7, 10, 14, 21, 28, 60, 90, 180 and years 1, 2 and 3.
- The relative abundance (ratio to baseline) of gene expression (measured by gene expression microarrays and/or mRNA-seq technologies) at 0, 12 hours and days 1 to 14, 21, 28, 60, 90, 180 and years 1, 2 and 3 years.
- Geometric mean salivary IgA antibody concentration to *Salmonella* Typhi O, H and Vi antigens at day 0 and days 1 to 14, 21, 28, 60, 90 and 180.
- Mean faecal lactoferrin concentration on days 0, 4, 7, 10, 14, 21 and 28.
- Geometric mean concentration of total faecal IgG and IgA and specific IgG and IgA (including IgA1 and IgA2 subclass determination) antibodies against *Salmonella* Typhi O, H and Vi antigen in stool samples at days 0, 7, 14, 28, 180 and years 1, 2 and 3.
- Exploratory analysis for presence of urine biomarkers by mass spectrometry

Additionally, for participants given a diagnosis of typhoid fever (see section 0) the following descriptive analyses will also be made, for all infected participants combined and for infected participants by vaccine group:

- Median time from challenge to ***clinical*** diagnosis of typhoid fever (i.e. oral temperature ≥38^0C for ≥12 hours, not development of bacteraemia).
- Individual and median time to *Salmonella* Typhi bacteraemia (as detected by blood culture).
- CRP level 0, 24, 48 and 96 hours after diagnosis of typhoid fever.
- Mean number of *Salmonella* Typhi CFU in blood at diagnosis (hour 0) of typhoid fever.
- Mean number of *Salmonella* Typhi CFU in stool at days 1 to 4 after diagnosis of typhoid fever.
- Geometric mean IgG, IgM and IgA antibody concentrations to *Salmonella* Typhi O, H and Vi antigens at hours 48 and 96 after diagnosis of typhoid fever.
- Geometric mean IgG, IgM and IgA antibody concentrations to *Salmonella* Typhi LPS, membrane preparation and whole cell in lymphocyte supernatant, at hours 0, 48 and 96 after diagnosis of typhoid fever.
- Geometric mean S*almonella* Typhi specific serum bactericidal antibody (SBA) titres at hours 0, 48 and 96 after diagnosis of typhoid fever.

- Geometric mean number of cells per 10^6 PBMCs, secreting IgG and IgA antibody in response to *Salmonella* Typhi O, H and Vi antigens at 48 hours after the diagnosis of typhoid fever.
- Mean proportion of specific memory T cell subsets (including T effector/memory, T_{EM}; T central/memory, T_{CM}; CD45RA$^+$ T_{EM}, T_{EMRA}) able to secrete IFN-γ, TNF-α and/or IL-2 measured by flow-cytometry at 48 and 96 hours after diagnosis of typhoid fever.
- Mean frequency of specific memory B cells to *Salmonella* Typhi antigens (including LPS and flagella) measured by ELISPOT on expanded PBMCs at hours 48 and 96 after diagnosis of typhoid fever.
- Mean proportion of cells secreting cytokines in response to soluble *Salmonella* Typhi antigens (e.g., flagella, OmpC, GroEL) and *Salmonella* Typhi-infected targets measured by flow-cytometry and/or ELISPOT (expressed as mean spot counts per 10^6 PBMCs) at hours 48 and 96 after diagnosis of typhoid fever.
- Measurement of the relative abundance (ratio to pre-vaccination level) of gene expression (measured by gene expression microarrays and/or mRNA-seq technologies) at diagnosis of typhoid and 0, 6, 12, 24, 48, 72 and 96 hours after diagnosis of typhoid fever.
- Geometric mean salivary IgA antibody to to *Salmonella* Typhi O, H and Vi antigens, 0, 12, 24, 48, 72 and 96 hours after diagnosis of typhoid fever.

3. STUDY PARTICIPANTS

Overall description of study participants

Male or female participants aged 18-60 years who are in good health (as determined by a study doctor, medical investigation and agreement of their general practitioner) and who are able to provide written informed consent, will be eligible for inclusion in this study.

Inclusion Criteria

Participants must satisfy each of the following criteria to be eligible for study inclusion:

- Male or female aged 18 - 60 years inclusive.
- Willing and able to give informed consent for participation after the nature of the study has been explained.
- In good health as determined by:
    - a) Medical history
    - b) History-directed physical examination
    - c) Clinical judgment of the investigators.
- Have an abdominal ultrasound scan result documented demonstrating no evidence of gallbladder pathology or cholelithiasis/gall stones (see sections 0 and 0).[ii]
- Able and willing (in the opinion of the investigators) to comply with all study requirements, including capacity for good personal hygiene.
- Able and willing to remain in England for 21 days after vaccination.[iii]
- Willing to allow their general practitioner and/or hospital consultant (if relevant), to be notified of participation in the study.
- Willing to allow the Health Protection Unit to be informed of participation in the study.
- *For those involved in provision of health or social care to vulnerable groups* ***only*** – willing to allow their employer to be notified of participation in the study
- Willing to give their close contacts (defined as someone who is likely to have been exposed to the excreta of a challenged participant, usually a household or sexual

---

[ii] An individual who has had a cholecystectomy will still be eligible, but may still require an ultrasound scan to be performed.

[iii] As per DEFRA application for GMO release.

contact) letters informing them of the participants involvement in the study and offering the contacts screening for *Salmonella* Typhi carriage.

- Agree to refrain from blood donation (to the National Blood Service) in the future if they are diagnosed with typhoid fever.
- Be willing to have 24-hour contact with study staff during the four weeks post-challenge.

Exclusion Criteria

The participant may not enter the study if **ANY** of the following apply:

- Are unwilling or unable to give written informed consent to participate in the study.
- Have previously received any typhoid vaccine.
- Have previously been resident in a typhoid endemic country for >6 months.
- Have previously been diagnosed with probable or confirmed typhoid infection.
- Have previously been challenged with *Salmonella* Typhi or enrolled in a typhoid challenge study.
- Have any known or suspected impairment or alteration of immune function, resulting from, for example:
    - Congenital or acquired immunodeficiency (including IgA deficiency),
    - Human Immunodeficiency Virus infection or symptoms/signs suggestive of an HIV-associated condition,
    - Autoimmune disease.
- History of significant cardiovascular disease (including congenital heart disease, previous myocardial infarction, valvular heart disease (or history of rheumatic fever), previous bacterial endocarditis, history of cardiac surgery (including pacemaker insertion), personal or family history of cardiomyopathy or sudden adult death).
- History of significant respiratory disease (e.g., uncontrolled asthma, chronic obstructive pulmonary disease).
- History of significant endocrine disorder (e.g., diabetes mellitus, Addison's disease).
- History of significant renal or bladder disease (including history of renal calculi).
- History of biliary tract disease (including biliary colic and/or gallstones, and asymptomatic gallstones detected by ultrasound screening).

- History of significant gastrointestinal disease (including inflammatory bowel disease, abdominal surgery, coeliac disease, liver disease (including hepatitis B or C infection, (as determined by detected hepatitis B surface antigen or hepatitis C antibody)), or requirement for H_2-receptor antagonists, proton pump inhibitors or laxatives).
- History of significant neurological disease (including seizures and myasthenia gravis).
- History of significant metabolic disease (e.g., glucose-6-phosphate dehydrogenase deficiency).
- History of significant haematological diagnosis (including anaemia, bleeding diathesis and sickle cell disease).
- History of psychiatric illness requiring hospitalisation, current known or suspected drug or alcohol misuse (defined as an alcohol intake exceeding 42 units per week).
- Moderate or severe depression or anxiety as classified by the Hospital Anxiety and Depression Score at challenge, that is deemed clinical significant by the Chief Investigator or consultant physician. If elevated scores are due to temporary life-events, the questionnaire may be repeated after resolution of the event with a view to inclusion if normalised.
- History of significant infectious disease (e.g., previous or current schistosomiasis infection, history of positive syphilis serology (determined by non-treponemal test), stool examination positive for an enteric pathogen at screening).
- History of non-benign cancer (except squamous cell or basal cell carcinoma of the skin and cervical carcinoma in situ).
- Presence of any implants or prostheses (e.g., artificial joints, pacemakers).
- Any clinically significant abnormal finding on biochemistry or haematology blood tests or urine analysis as assessed using **Table 4**.
- Hypersensitivity to any component of the vaccine or are hypersensitive to two or more of the following antibiotics: ciprofloxacin, azithromycin, ampicillin, trimethoprim sulfamethoxazole.
- Female participant who is pregnant, lactating or who is unwilling to ensure that they or their partner use effective contraception one month prior to vaccination and continue to do so until two negative stool samples obtained a week apart, a minimum of 1 week after completion of antibiotic treatment have been obtained.
- Current occupation involving:

- clinical or social work with direct contact with young children (defined as those attending pre-school groups, nursery or aged less than 2 years),
- highly susceptible patients or persons in whom typhoid infection would have particularly serious consequences (i.e. those who are immunocompromised or debilitated),
- care work involving the elderly.

Exemption: those willing not to work from point of vaccination until demonstrated to not be infected with *Salmonella* Typhi (in accordance with Health Protection Agency guidance).

- Current occupation as a commercial food handler involving the preparation or serving of unwrapped foods not subjected to further heating.
- Household contact with a young child (defined as above).
- Household/close contact who is immunocompromised (due to treatment, e.g., chemotherapy, or illness, e.g., HIV infection).
- Scheduled elective surgery or other procedures requiring general anaesthesia during the vaccine/challenge period, at time of enrolment.
- Participants who have taken part in other research involving an investigational product (IMP) that might affect risk of typhoid infection or compromise the integrity of the study within the 30 days prior to enrolment (e.g., significant volumes of blood already taken in previous study), as assessed by both participant questioning and 'The Over Volunteering Prevention System' (TOPS) database.
- Have received blood, blood products and/or plasma derivatives including parenteral immunoglobulin preparations in the previous 3 months
- Any other significant disease or disorder which, in the opinion of the investigator, may put the participants at risk because of participation in the study, may influence the result of the study, or affect the participant's ability to participate in the study.

Temporary exclusion criteria for vaccination visits

- Fever >37.5^{o}C within 24-hours prior to vaccination.
- Acute gastrointestinal illness within 24-hours prior to vaccination.
- Antibiotic therapy during the 14 days prior to vaccination or plan to take antibiotics in the period of vaccination until 14 days after the last dose of vaccine has been administered.

- Receipt of immunosuppressive treatment/therapy such as chemo- or radiotherapy within the preceding 6 months or long-term systemic corticosteroid therapy,[iv]

  or, any systemic corticosteroid (or equivalent) treatment within 14 days prior to challenge, or for more than 7 days consecutively within the previous 3 months.

- Receipt of another live vaccine within 4 weeks prior to vaccination or a killed vaccine within 7 days prior to vaccination
- Plan to receive any vaccine other than the study vaccine within 4 weeks following vaccination.
- Therapy with antacids, proton pump inhibitors or H_2-receptor antagonists in the 24-hours prior to vaccination.
- Unavailable for challenge visit at 28 days (+/- 5 days) following vaccination.
- Significant blood donation within the preceding 3 months (e.g., to the National Blood Service).

Temporary exclusion criteria to challenge with *S.* Typhi (Quailes strain)

- Have experienced significant acute or exacerbation of chronic infection within the previous 7 days or have experienced fever (>37.5°C) within the previous 3 days or on the day of challenge (enrolment).
- History of antibiotic therapy within the previous 14 days.
- Any significant corticosteroid treatment (such as prednisolone or equivalent) within the 14 days prior to challenge, or for more than seven consecutive days within the previous 3 months.
- Therapy with antacids, proton pump inhibitors or H_2-receptor antagonists within 24-hours prior to challenge.

Potential risks to participants

The general risks to participants in this study are associated with venepuncture, use of the IMP (M01ZH09 vaccine) and challenge with live typhoid-causing bacteria. Potential anticipated risks/complications arising from taking part in this study and the measures that are to be taken to avoid them are summarised below.

[iv] greater than 10mg oral prednisolone (or equivalent) daily within last 3 months.[80.] Committee., J.F. British National Formulary. (ed. Society, B.M.A.a.R.P.) (London, 2011).

Venepuncture

Venepuncture will be performed as described in section 0 and according to OVG SOP 009 version 7; *Taking venepuncture samples*. The volume of blood taken should not compromise an otherwise healthy adult and is compliant with the guidance supplied by the National Blood Service.[81] Participants will be closely monitored, however, both clinically and by laboratory parameters. Should participants develop evidence of anaemia, further advice will be sought from the Chief Investigator or Consultant Physician. Consent will be sought from all participants for their details to be registered with The Over-volunteering Prevention System (TOPS).[82] This will prevent inadvertent over-volunteering and thus also excess blood donation.

Complications of use of the study vaccine, M01ZH09

Clinical studies of the IMP (M01ZH09 vaccine) to-date involving both healthy adults (in the UK, USA and Vietnam)[63,64,83,84] and healthy children (Vietnam)[65] have demonstrated that the vaccine is safe and well tolerated. The use of M01ZH09 is therefore unlikely to pose any significant risk to participants. Side-effects reported in previous studies with a frequency of 5% or more include headache, flatulence, abdominal pain, nausea, diarrhoea, asthenia, myalgia, constipation, fatigue, anorexia, pyrexia and musculoskeletal pain.[60] The vast majority of these side-effects have been of short duration and mild severity. There is a theoretical risk of allergic reaction, but this has yet to be observed with the use of this vaccine. M01ZH09 (and the positive control, Ty21a) vaccine is a live, attenuated vaccine and therefore poses the theoretical risk of participant infection and also of dissemination to non-immunised subjects via faecal excretion.

The presence of either vaccine strain has not been detected in the blood of immunised volunteers (vaccinaemia). Nonetheless, vaccinaemia remains a theoretical risk and is known to occur with other licensed live vaccines without consequence; in this case it could lead to a clinical illness consistent with typhoid fever. In the unlikely event of this occurring, treatment with antibiotics is expected to fully resolve any illness.

Faecal excretion of the vaccine strains is known to occur up to 17 days after vaccination; however infection of a non-vaccine recipient has not been reported. Therefore, the risk from using an attenuated vaccine strain is thought to be negligible, particularly with the high standard of sanitation practiced in the UK.

Complications of typhoid Fever

Study participants may develop symptomatic typhoid infection following challenge, as demonstrated in the initial dose-finding study. Participants will be reviewed at least daily by a member of the study team and will, in addition, be telephoned every evening to ensure that

participants are well. Participants will be instructed to record their oral temperature, with a thermometer provided, twice-daily and if they feel feverish, and will be instructed to contact the study team immediately should they manifest any signs or symptoms they perceive as serious or worrying. The further management of typhoid fever is outlined in section 7. Fluoroquinolone antibiotics are the treatment of choice for typhoid fever and are generally well tolerated and rapidly efficacious. If any participant is unexpectedly unwell then an additional review will be arranged, if necessary by a study doctor visiting the participant in his or her own home. This has been required for two visits among the 5 participants challenged in the dose-finding study so far.

Complications of typhoid fever such as perforation or haemorrhage occur almost exclusively in patients who go without antibiotic treatment for an extended period of time. Participants in this study will be treated 12 hours after developing fever or if *Salmonella* Typhi is recovered from a blood culture drawn after day 5 (see section 0). They will be closely monitored during the initial study phase and until a 14 day course of antibiotics is completed, so that the risk of complications occurring is minimal.

The risks associated with typhoid challenge will be greatly minimised by complying with study visits and maintaining close contact with the study team. This will be emphasised at screening and throughout the study. Participants will be made aware of the potential symptoms of typhoid and will be monitored closely throughout the challenge for the development of these symptoms. Previous challenge studies using the same strain of *S.* Typhi undertaken in the 1960/70s at the University of Maryland and the current challenge study at OVG have demonstrated a good safety profile.

Relapse of typhoid Fever

Relapse rarely occurs after treatment with 14 days of ciprofloxacin; shorter durations of therapy have been associated with infrequent relapses.[73,74] For this reason we will use a 14 day course and review participants regularly for symptoms or laboratory evidence of relapse.

Chronic carrier state

A chronic carrier state in which *Salmonella* Typhi is excreted in the stools for many years without illness can develop after *S.* Typhi ingestion.[83] The chronic carrier state is usually seen in older women with pre-existing gallbladder disease (mainly gallstones); in this study, therefore, only participants with a normal ultrasound examination of the gallbladder will be included (see section 0). The likelihood of developing chronic carriage is extremely low however, particularly with newer antibiotics such as the fluoroquinolones being available. A previous study demonstrated that, of more than 200 patients treated for typhoid fever with ciprofloxacin, none became carriers.[73]

To ensure clearance of infection and to exclude chronic carriage, stool samples for culture will be obtained upon completion of the initial antibiotic course (section 0). Should chronic carriage occur (defined as stool cultures being positive for *Salmonella* Typhi 4 weeks after completion of antibiotics) then the participant will be referred to an Infectious Diseases Consultant at the Oxford Radcliffe Hospitals NHS Trust for further management.

Antibiotics

Potential participants with known antibiotic hypersensitivity or allergy to two or more of the first-line antibiotics (ciprofloxacin, azithromycin, ampicillin, trimethoprim sulfamethoxazole) will be excluded. The antibiotics to be used are generally well tolerated and only occasionally associated with side effects. Should an antibiotic cause allergy or intolerance this will be managed by a study physician and a different antibiotic used for subsequent management.

Pregnancy and contraception

The possible adverse effects of *Salmonella* Typhi infection or the effect of some antibiotics (including ciprofloxacin) on the outcome of pregnancy are unknown therefore pregnant women will be excluded from the study. If relevant, non-pregnant female participants will be required to use an effective form of contraception from 30 days prior to initial vaccination until deemed to be clear of infection (see section 7.7) – this will be at least 4 weeks following completion of the antibiotic course (see **Figure 2** below).

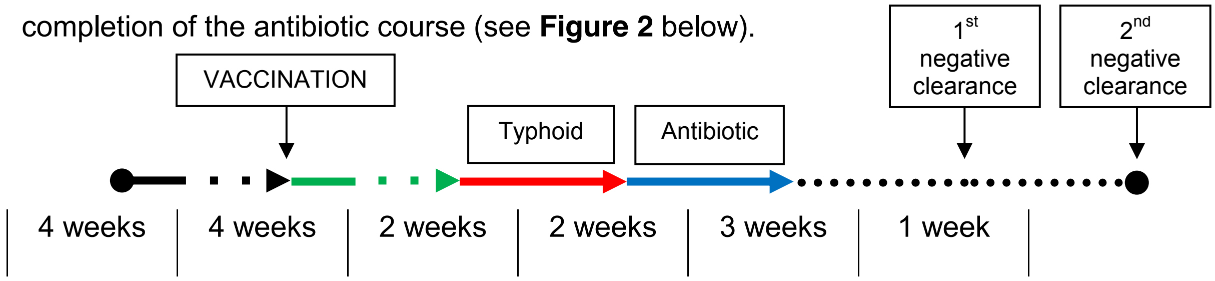

Figure 2: Duration of contraception to be used during study, OVG 2011/02

Diarrhoea and antibiotics can reduce the efficacy of oral hormonal contraceptives ('the pill') by altering absorption. For this reason female participants who are taking oral contraception will be advised to use additional barrier contraception should they experience symptoms of diarrhoea, whilst taking antibiotics and for 7 days after completion of antibiotics.

Should pregnancy occur, information about outcome of the pregnancy will be sought.

Person-to-person spread of *S.* Typhi

In view of the low infectivity of *Salmonella* Typhi without bicarbonate buffer and the high standard of hygiene and sanitation in the UK, secondary transmission of either the vaccine or challenge strain to household or other close contacts after discharge is highly unlikely.

Participants will be provided with hand soap and paper towels for use during the challenge period and will be counselled on the necessity of adequate hand hygiene. This will include written instructions on how to employ enteric precautions and participants will be taught and then observed demonstrating hand washing techniques to ensure they have understood how to avoid transmission.

Household or close contacts of participants will be offered the opportunity to be screened for *S.* Typhi by examination of up to 2 stool specimens (described in section 0).

Potential risks to participant contacts

In view of the low infectivity of *Salmonella* Typhi without gastric acid suppression and the general level of hygiene and sanitation in the UK, secondary transmission of either *Salmonella* Typhi vaccine strains (M01ZH09 or Ty21a) or challenge strain to household or other close contacts is highly unlikely. It is thought that *Salmonella* Typhi, unlike *Shigella sp.*, enterohaemorrhagic *Escherichia coli* or hepatitis A virus, is virtually never transmitted by direct faecal-oral contact. This is in part due to the higher oral inoculum of *Salmonella* Typhi bacteria required to cause clinical disease. The only (rarely) reported exception is direct transmission by ano-lingual sexual contact.[8]

It is acknowledged, however, that transmission within households can occur if the individual excreting *Salmonella* Typhi fails to practice effective hand washing after defecation and is subsequently involved in uncooked food preparation. If food is kept at ambient temperatures, bacterial proliferation occurs such that an infective dose level is reached, and the food then may act as a vehicle for typhoid transmission.

Throughout the period of possible excretion of the vaccine or challenge strains the participants must practice stringent hand washing techniques after defecation. Participants will be given soap and paper towels for use at home and detailed advice on how to prevent transmission of *Salmonella* Typhi. Participants will be taught and observed practicing good hygiene technique at their initial challenge visit (V1, Day 0). The importance of adhering to sanitation advice will be emphasised to participants at each visit. It is important to note that participants in this trial will be fully informed about the risks of transmission and how to prevent this prior to challenge. As such, participants will be in the position to implement this from the point of infection thus reducing the chances of secondary transmission. This is very different from the situation with travellers returning from abroad where diagnosis is usually delayed by several weeks allowing a prolonged period of exposure to contacts before precautions are put in place. In practice, since most individuals living in developed countries practice good personal hygiene and food hygiene, secondary transmission of *S.* Typhi within households by returning travellers with typhoid fever is rare. Furthermore, the delay in diagnosis that occurs in travellers with typhoid fever leads to a prolonged period of time in

which *S.* Typhi has been excreted. We will treat all participants in this trial very early in the course of disease, leading to rapid clearance of bacteria and a very limited period of excretion and thus potential exposure to contacts.

When occasional transmission of typhoid fever occurs, it is usually related to unknowingly infected food handlers,[6,84] and we will exclude food handlers from this study (section 0). Potential participants employed in clinical or social work with direct contact with young children (those attending pre-school groups, nursery or aged less than 2 years of age) or highly susceptible patients or persons in whom typhoid infection would have particularly serious consequences (such as the elderly) also represent an increased risk and will be excluded unless willing to not work until it has been demonstrated that they are not infected with *Salmonella* Typhi in accordance with Health Protection Agency guidance.[85]

Even in the absence of precautions to prevent secondary transmission (as is seen in returning travellers), the rate of transmission is exceptionally low within the UK. In a large recent study of 251 contacts of patients with typhoid fever in London, only 1 person was identified as a suspected case of secondary transmission.[86] Similarly a study in Scotland showed a maximum secondary transmission rate in the absence of precautions to be 6 out of 267 contacts.[87]

Potential benefits

Participants will not directly benefit from participation in this study. However, it is hoped that the information gained from this study will contribute to the development of an understanding of typhoid fever. Potential benefits for participants would be information about their general health status, primarily obtained at the screening visit, and possible vaccine or infection-derived protection from future typhoid infection. Participants will be reimbursed *pro rata* for costs associated with travel and time taken off work, as appropriate (see section 0).

4. RECRUITMENT, ASSESSMENT AND RANDOMISATION

Recruitment and pre-screening

Adult participants will be recruited by advertising and by using an information booklet containing detailed information about the study. In order to recruit the required sample of 99 participants several advertising strategies will be employed, including:

- Direct mail-out: this will involve obtaining names and addresses of adults from the most recent Electoral Roll. The contact details of individuals who have indicated that they do not wish to receive postal mail-shots would be removed prior to OVG being given this information. The company providing this service is registered under the Data Protection Act 1998. OVG would not be supplied with the dates of birth or ages of individuals; only the names and addresses of individuals aged over 18 years (as per the inclusion criteria) would be provided. Some individuals may be over the 60 year age limit – therefore a compliments slip will be enclosed with the information leaflet to apologise for contacting them inappropriately.
- Poster campaign: posters will be displayed in local hospitals (of the Oxford Radcliffe Hospitals NHS Trust), local GP surgeries, tertiary education institutions and other public places with the permission of the owner/ proprietor.
- Local newspaper advertisements: adverts will be placed in local newspapers with brief details of the study and contact details to obtain further information.
- CCVTM and OVC databases: we will contact individuals using databases supplied by other groups based within the Clinical Centre for Vaccinology and Tropical Medicine and Oxford Vaccine Centre. These databases contain information relating to participants in previous studies/trials, who have expressed an interest in receiving information about future studies for which they may be eligible. This will include individuals expressing interest in the previous dose-finding typhoid challenge study (OVG 2009/10).
- E-mail distribution: e-mails will be sent to various distribution groups or lists with the express agreement of the network administrator or equivalent authorisation.
- Internet display: a description of the study and copy of the information booklet on the Oxford Vaccine Group website and/or a link to a dedicated study webpage.
- Exhibitions: advertising material and/or persons providing information relating to the study will exhibit using stalls or stands at exhibitions and/or fairs, such as University Fresher's Fairs.

Inclusion and exclusion criteria will be made available to interested parties, in advance of the formal screening visit (via the study website or telephone). Potential participants will be encouraged to make an appointment only if they have seen and meet the inclusion and exclusion criteria. This will serve to prevent potential participants having to attend the study site unnecessarily if they will be automatically excluded subsequently, due to the extensive criteria.

Once an expression of interest has been received, potential participants will be contacted by telephone by one of the OVG study doctors or nurses to discuss the study and screen for inclusion and exclusion criteria. If they are still keen to proceed an appointment would be made for them to attend the Centre for Clinical Vaccinology & Tropical Medicine (CCVTM) on the Churchill Hospital site, where informed consent would be taken after a detailed description of the study, its rationale and the risks and benefits of taking part have been explained. The remainder of the screening visit would ensue as described below.

Informed Consent

The participant must personally sign and date the latest approved version of the informed consent form before any study specific procedures are performed. Consent will be sought as described in OVG SOP 030 version 2; *Participant Enrolment into Studies: Screening, Consent and Enrolment*.

Written and verbal versions of the participant information booklet and informed consent form will be presented to the participant, detailing no less than:

- the exact nature of and the rationale for performing the study;
- implications and constraints of the protocol;
- known efficacy and side-effects of vaccines being used;
- likely effects and outcome of being given the challenge agent;
- the risks and benefits involved in taking part.

In addition, participant consent will be required to provide the name and 24-hour contact telephone number of a close friend, relative or housemate who lives nearby, to be held by the study investigator. The participant will consent to keeping this person informed of their whereabouts for the duration of the study (until confirmed clear of infection, see section 0). This person would be contacted if study investigators were unable to contact the participant. The 24-hour contact will receive written information and be required to complete and sign a reply slip, which will be returned to the study doctor/nurse before challenge.

Consent will also require participants to inform their close contacts of involvement in the study; the participant will supply self-identified contacts with letters provided by us, detailing

the study and offering screening for typhoid carriage, if desired (see section 0). The low risk of spread will be emphasised to participant contacts to avoid undue anxiety. Consent will also be taken for allowing the relevant Health Protection Unit to be informed of participation. If the participant is involved in the provision of health or social care to vulnerable groups then consent will be taken to inform his/her employer of their participation in the study.

It will be clearly stated that the participant is free to withdraw from the study at any time for any reason without prejudice to future care, and with no obligation to give the reason for withdrawal. However, the need for treatment with a course of antibiotics if withdrawal occurs after challenge will be emphasised. Participants who withdraw after challenge may also be asked to attend additional visits for safety reasons, as deemed necessary by the study investigators. With consent, all participants enrolling onto the study will be added to the TOPS registry.[82]

The participant will be allowed as much time as wished to consider the information provided and to ask questions of the study team, their GP or other independent parties in order to form a decision regarding whether or not to participate in the study. Written informed consent will then be obtained by means of participant dated signature and dated signature of the investigator who presented and obtained the informed consent. The person who obtained the consent must be suitably qualified and experienced, and have been authorised to do so by the Chief Investigator. A copy of the signed informed consent will be given to the participants. The original signed form will be retained at the study site.

Screening and eligibility assessment

After supplying informed consent, participants will be assessed by a member of the study team to ensure that they satisfy the inclusion/exclusion criteria and to aid data analysis. Screening procedures including medical assessment and laboratory blood and urine tests will be performed within 90 days prior to vaccination (i.e. Day -120 to -30) to remain valid. If this duration is longer, then the informed consent process will be repeated together with any investigations required at the clinical judgement of the Chief Investigator and Consultant Physician.

The screening and eligibility assessment will include the recording of:

- Demographics: date of birth, ethnicity and gender.
- History of participating in previous typhoid challenge studies.
- Query of the TOPS database using the participant’s National Insurance or passport number.

- Medical history: details of any significant medical or surgical history based on participant recall. If medical clarification is required, medical notes and/or discussion with other medical practitioners will be undertaken. Medical history will be detailed in a letter to the participant's General Practitioner. The GP will be asked to return a form confirming the history as given and stating that they know of no other medical reasons why the participant should not be included. This is required prior to study enrolment.
- Concomitant medication: all current medication, medication taken for >7 days in the preceding 6 months and any medication or other vaccinations taken within the 4 weeks prior to vaccination, including herbal supplements and over-the-counter medication.
- Contraception history: female participants will be asked if they or their partners are willing to use effective barrier contraception from one month prior to vaccination until deemed to be clear from infection (see section 0).
- Typhoid immunisation history: participants will be questioned as to if they have previously received typhoid or travel vaccines. Those who have attended travel clinics or have had vaccines for travel but deny or are unsure if they have received a typhoid vaccine will require confirmation in writing from their GP.
- Social history: including history of tobacco, alcohol or other drug use; questioning regarding any personal or domestic reason that may lead to concern regarding an individual's ability to maintain good personal hygiene; history of living in a typhoid endemic country for more than 6 months.
- Physical examination including vital signs, cardiovascular, respiratory, abdominal and gross neurological exam.
- Psychological assessment including use of the Hospital Anxiety and Depression Score.[88]
- Bedside investigations: including: a 12-lead electrocardiograph, dipstick testing of mid-stream urine (with laboratory analysis if abnormal) and performance of a pregnancy test for females (exception: women who are menopausal (defined as having not menstruated for 1 year) or who have had tubal ligation).
- Blood test for full blood count, erythrocyte sedimentation rate, C-reactive protein, serum creatinine, urea, electrolytes, glucose (if indicated as being necessary from the urine dipstick result), liver function tests (AST, ALT, bilirubin, alkaline phosphatase, amylase), anti-endomysial and total serum IgA antibodies.
- Abdominal ultrasound scan (to screen for gallbladder disease).

Additionally, participants will be provided with a letter for their 24-hour contact (to bring completed reply slip at visit 1) and for their close contacts (see section 0).

All laboratory results will be reviewed and the reports signed by a study doctor who will record in the CRF whether they are normal, abnormal but not clinically significant, or abnormal ***AND*** clinically significant. Abnormality of these tests will be assessed in accordance with **Table 4** (section 0) and at the discretion of the study investigators. If results are abnormal and clinically significant the test may be repeated to ensure it is not a single occurrence. If a test remains clinically significant, the participant will be informed and the eligibility of the participants will be reviewed with the chief investigator and/or consultant physician. Appropriate medical care for any abnormal result or medical finding will be arranged with the participant's permission.

Details of the screening assessment will be recorded in the CRF. If the inclusion/exclusion criteria are satisfied and informed written consent has been obtained, the participant will undergo vaccine randomisation (see below).

Participants recruited and successfully screened for the previous dose-finding study (OVG 2009/10) but who have not been challenged, continue to fulfil the inclusion and exclusion criteria and who provide full written consent to this study, may forego the re-screening tests as long as they still remain within the valid time period (see above).

Randomisation and code breaking

Randomisation will be provided by the Centre for Statistics in Medicine (CSM), University of Oxford, using varying block sizes with an allocation ratio of 2:1 to M01ZH09/vaccine placebo or the positive control (Ty21a vaccine), and then 1:1 allocation to M01ZH09 or vaccine placebo. A randomisation list will be generated and sequentially numbered opaque sealed envelopes will be provided for the investigators. Once eligible to take part in the study, a study team member will open the next randomisation enveloped to reveal the group allocation. This will be recorded in the CRF and the participant will be notified in order to schedule further visits. The randomisation-code break list, which will be password protected, will be kept in confidence at the CSM. **At the point of randomisation the participant will be considered enrolled into the study**.

M01ZH09/vaccine placebo arm

The M01ZH09/vaccine placebo arm will be double blinded, such that neither the investigator nor the participant knows which vaccine has been given/received. The allocation of participants within the M01ZH09/ placebo arm to each treatment will be by means of blinded packaging containing either M01ZH09 or placebo, in a 1:1 distribution. Participants allocated to the M01ZH09/placebo group will be assigned the lowest available numbered kit(s)

provided by Emergent BioSolutions (labelled by Aptuit Ltd.) that will contain either vaccine or placebo. Randomisation code breaks will be held by the Statistician at CSM to unblind this arm if required. The code breaks will only be used if a situation arises where it is deemed necessary by the Chief Investigator to break the blinding process.

All blinded participants and study team members will be informed of the vaccine received 28 days post-challenge with *Salmonella* Typhi (Quailes strain). Investigations after this time point are based on immunological and microbiological parameters in body fluids and will therefore not be influenced by the un-blinding of participants.

Positive control arm (Ty21a vaccine)

Participants randomised to the positive control arm, will receive open-label Ty21a vaccine, as the aim of this arm is to demonstrate the scientific integrity of the model by demonstrating the known protective effect of vaccination. Also, the formulation of this vaccine and the dosing schedule is different from the investigational agent. Open-label Ty21a vaccine will be given as 3 doses, 48-hours apart on days –32, -30 and -28.

Functional genomics subgroup

A subgroup of up to 30 participants (maximum) will have additional visits during and after vaccination in order to investigate the functional genomic response to vaccination and to provide stool samples for investigation of the faecal microbiome. Participants will be asked in order of recruitment to the study if they are willing to participate in the functional genomic analysis. Therefore allocation to this subgroup will not be randomised.

5. VACCINATION PROCEDURES

Initial vaccination visit

The visit procedure for the initial vaccination visit will be as follows:

- Ensure that participant consent remains valid and, if so, request that they sign the informed continued consent form.
- Ask whether they would be prepared to attend for additional (compensated) visits for inclusion into the functional genomics sub-group (section 0).
- Obtain and document interim medical history since screening and check eligibility criteria (specifically temporary exclusion to vaccination).
- Record oral temperature.
- Perform urinary pregnancy test for females (exception: women who are menopausal (defined as not having menstruated for 1 year) or who have had tubal ligation).[v]
- Confirm with participant as to whether they are in the M01ZH09/placebo blinded arm or the positive control (Ty21a) group (randomisation having been performed prior to the first vaccination visit, see section 4.4).
- Ensure the participant has been fasting for one hour or more *before* vaccine dosing.
- Perform blood draw as per **Table 2** (see section 0 and OVG Clinical Study Plan).
- Obtain 20mL midstream urine sample for urine dipstick testing and mass spectrometry.
- Collect stool and saliva specimens for baseline immunological, bacteriological and lactoferrin measurements.
- Administer either vaccine or placebo, as per randomisation group
  - If Ty21a, administer with a cold or lukewarm drink if necessary. Advise the participant that the capsules should not be chewed and should be swallowed as soon as possible after placing in the mouth.
  - If M01ZH09 or placebo, use the next lowest available numbered kit for randomisation via packaging to occur.
- Fast participant for one hour *after* dosing (with the exception of clear fluids).
- Record all doses given on Study Vaccination Record Card.
- Complete letter to the GP with details of the vaccination visit, including whether the participant has been allocated to a blinded arm or has been vaccinated with the Ty21a vaccine.

---

[v] Any positive pregnancy tests will be managed according to OVG SOP 023 version 2; *Positive pregnancy test referral procedure*.

- Register participant with TOPS.[82]
- Provide participant with study centre contact details (including 24-hour telephone contact details for study investigator/clinician).
- Instruct participant on notifying study centre of any serious adverse events/reactions.
- Instruct participants to use antipyretics only to treat fever or other adverse reactions, rather than pre-emptively.
- Issue participant with oral thermometer and instruct in its use.
- Provide participant with a Diary Card to detail systemic effects, AEs and concomitant medications from day of first vaccination to 7 days after the last vaccination dose is given.
- Issue participant with an enteric precautions leaflet and advise regarding the need for preventing transmission of live vaccine strains.
- Advise female participants using hormonal contraceptives that additional barrier contraception should be used should they develop diarrhoea following vaccination.
- Schedule further visits as follows:
    - If in Ty21a arm, further vaccination visits to give doses 2 and 3 of the vaccine (days -30 and -28).
    - If in functional genomics subgroup, see section 0.
    - For all participants, vaccination follow-up visits in 7 and 14 days (i.e. at days -21 (Va) and -14 (Vb), respectively).
    - Challenge visit, V1, 28 days after vaccination (i.e. at day 0).

If a participant vomits within one hour of the vaccination being administered, a study investigator will decide whether to administer a further vaccine dose with the participant's consent or withdraw the participant from the study.

Further Ty21a vaccination visits

Two subsequent visits will be required to complete the Ty21a course. These will be required at days -30 and -28, such that the vaccine is administered in 3 doses on alternate days. Theses visits will be conducted as follows:

- Obtain interim history and check eligibility criteria and for occurrence of any serious adverse event.
- Record oral temperature.
- Ensure participant has fasted for at least one hour.
- Obtain blood sample for functional genomics as per **Table 2**, if applicable.

- Administer vaccination with a cold or lukewarm drink if necessary. Advise the participant that the capsules should not be chewed and should be swallowed as soon as possible after placing in the mouth.
- Instruct the participant to fast for a further hour.
- Record all doses given on Study Vaccination Record.
- Arrange further visits as necessary (i.e. for third vaccine dose or for challenge).

Vaccination visits will occur at day -28 (+/-3days) for participants allocated to the M01ZH09/placebo arm and day -32 (+/-3 days) for those in the positive control (Ty21a) arm.

Recording vaccine-related side-effects

Participants will be instructed to complete a Diary Card, recording oral temperatures twice-daily and describing any symptoms or usage of any medications daily. The diary card will be completed from point of first vaccination for 7 days. At this stage the participant will be asked to document additional details regarding any visits seeking medical advice (including GP and Emergency Departments). The Diary Card will be reviewed when the participants attends for the post-vaccination visit, Va (day -21) (see section 0).

Additional functional genomics visits

For up to 30 participants consenting to additional functional genomic studies, additional compensated visits will be scheduled as required at days -32, -30, -28, -26 and -24. Thus, participants randomised to the Ty21a arm will have blood drawn at each vaccination visit (days -32, -30 and -28) and two subsequent visits (days -26 and -24); participants in the blinded M01ZH09/placebo arm will have blood drawn at the vaccination visit (day -28) and two subsequent visits (days -26 and -24). Participants attending these visits will also be asked to provide a stool sample for microbiome analysis at these time points.

The procedure for these visits will be as follows:

- Obtain interim history and check eligibility criteria, check for occurrence of any serious adverse event.
- Obtain blood for functional genomics (see OVG Clinical Study Plan).
- Obtain stool sample for microbiome analysis.

Vaccination follow-up visits

Follow-up visits after vaccination will be scheduled at Va (day -21) and Vb (day -14). The procedure for these visits will be:

- Obtain interim history and check eligibility criteria and for occurrence of any serious adverse event.
- Review diary card from vaccination period (day of first vaccination to 7 days after the last).
- Obtain blood samples as per **Table 2** (section 0).
- Collect urine, stool and saliva specimens for laboratory investigations.
- Schedule further visits as required.

6. *S.* TYPHI CHALLENGE PROCEDURE

Baseline assessment and challenge with *S.* Typhi (Quailes strain) will take place on Day 0, visit (V) 1. These procedures are described below, and in further detail in the OVG Clinical Study Plan.

Baseline assessment

- Obtain interim medical history, check eligibility criteria (specifically for temporary exclusion criteria to challenge) and for occurrence of any new adverse or medically significant events since previous visit.
- Check details of the 24-hour contact (who will be kept informed by the participant of their whereabouts for the subsequent 14 days) (see 0).
- Record oral temperature, resting pulse and blood pressure.
- Perform a pregnancy test for females (exception: women who are menopausal (defined as not having menstruated for 1 year) or who have had tubal ligation).
- If participant is in good health and is still suitable for inclusion in the study, perform blood draw as per **Table 2** (section 0) and OVG Clinical Study Plan.
- Obtain 20mL midstream urine sample for urine dipstick and mass spectrometry.
- Collect stool specimens and saliva samples for immunological, bacteriological and/or lactoferrin investigations.
- Instruct participant to fast for a minimum of 90 minutes prior to ingestion.
- Perform HADS (Hospital Anxiety and Depression Scale) assessment.[88]

Preparation of challenge agent

The solution for ingestion (containing *S.* Typhi (Quailes strain) will be prepared in a category 2 biological safety cabinet within a category 3 containment laboratory that has been industrially cleaned and is solely used for the purposes of preparing the solution. Preparation will be conducted by a study investigator and double checked by a second investigator, immediately prior to ingestion. The water and bicarbonate used for preparation will be commercially available food products. Containers for the solution will be single-use and disposed of after autoclaving following ingestion by the volunteer. The strain will be prepared as outlined in the OVG Clinical and Laboratory Study Plans.

Administration of *S.* Typhi (Quailes strain)

Salmonella Typhi challenge will be administered by the oral route with sodium bicarbonate at a dose of 1-5x10^4 CFU. Participants will be nil by mouth for 90 minutes before and after challenge. The procedure for administration is:

- Remove the prepared sodium bicarbonate solution and *S.* Typhi suspension from the BIOJAR.
- Ask the participants to drink the 120 ml of bicarbonate solution (prepared by dissolving 2 grams of $NaHCO_3$ in 150ml mineral water).
- Wait one minute.
- Ask the participants to ingest the 30ml *S.* Typhi/bicarbonate solution (prepared by mixing the required dose of challenge agent in the remaining 30ml mineral water).
- Dispose of containers that have contained *S.* Typhi in clinical waste bag suitable for autoclaving and autoclave in accordance with local guidelines.

Assessment after challenge

- Fast participant for a further 90 minutes.
- Participants who vomit for any reason within 90 minutes of the challenge will be withdrawn from the trial and treated with antibiotics as described in section 0.
- Complete a notification of challenge letter for the participant's GP.
- Complete a notification of challenge for the Thames Valley Health Protection Unit.
- Provide participant with study centre contact details (including 24-hour telephone contact details for study investigator/clinician).
- Instruct participant to notify study centre of any serious adverse events/ reactions that occur prior to next review.
- Instruct participant not to use antipyretics.
- Instruct participant to notify study centre when temperature ≥38°C.
- Provide participant with a Diary Card for recording systemic effects and twice-daily oral temperatures.
- Issue participant with a Medic Alert-type card containing information including the antibiotic sensitivity of the *S.* Typhi strain, study doctor contact details and instruction for the research team to be contacted immediately in the event of illness/accident.

- Check details of a mobile telephone number that the participant will be carrying with them for the 14 days post-challenge. Participants who do not have their own mobile telephone will be issued one for the duration of the study. Counsel the participant on the importance of keeping the mobile switched on and with them at all times.
- Issue participant with information on enteric precautions.
- Educate participant on correct hand washing technique, including demonstration and observation.
- Advise participants to inform study investigators if any breaches of enteric precautions occur such that another individual comes into contact with excreta from a participant.
- Issue participants with liquid hand soap and paper towels to aid with adherence to enteric precautions.
- Instruct participant on obtaining urine and stool/ rectal swab specimens (as outlined in the Clinical Study Plan) and provide the participant with sampling equipment.

Assessment 12 hours after challenge

Participants do not have to remain on site between assessments but a rest room will be provided which participants may use if they wish. Internet access will be available to participants that register with the University computer service as a guest of the OVG. This will require the participant to provide the computer department with their full name and to agree to abide by the University's terms and conditions of internet use.

- Perform blood draw as per **Table 2** (see section 0), such that samples arrive at the laboratory +/- 1 hour from scheduled visit time.

Subsequent assessments for all participants

Days 1 to 14 (V2-13)

Participants will attend the CCVTM for each visit at which the following will be undertaken:

- Review by study doctor or nurse of diary card from previous day.
- Record oral temperature, resting pulse and blood pressure.
- Perform blood draw as per **Table 2**, such that samples arrive at the laboratory +/- 3 hours from scheduled visit time for V2 and +/- 6 hours for subsequent visits (to V15).
- Collect stool samples daily for qualitative and quantitative culture (+/- microbiome analysis) and molecular analysis. Participants who do not pass stool in any 24-hour period will be asked to take a rectal swab for culture. Samples may be taken +/-12

hours from scheduled visit time, but should be delivered to CCVTM within 1 hour of being taken. If this is not possible then samples should be kept cool if possible.[vi]

- Examine stool for gross blood once a day.
- Collect 20ml mid-stream urine sample daily (+/- 12 hours of scheduled visit time). Samples should arrive at the laboratory within 1 hour of being taken.
- Collect saliva sample daily (+/- 12 hours of scheduled visit time). Samples should arrive at the laboratory within 1 hour of being taken.
- On days 7 and 14, ask participant to complete a HADS assessment.
- Instruct participant to notify study centre of any serious adverse events/ reactions that occur prior to next review.
- Instruct participant not to use antipyretics.
- Instruct participant to notify study centre if/when temperature ≥38.0°C.
- Remind participant grade severity of symptoms and record evening oral temperature in Diary Card.

Participants will be provided with a rest area at the CCVTM for use between visits but will not be considered to be admitted for inpatient care, unless subsequently fulfilling any of the criteria for admission (as per section 0). Any participant who develops symptoms severe enough to stop all normal activity will be admitted for observation irrespective of whether the definition of illness (i.e. typhoid diagnosis) has been met.

Follow-up telephone calls

Participants will be contacted by telephone twice daily from the day after challenge until completion of the antibiotic course, unless a visit at the CCVTM is scheduled. This will be by telephone call from one of the study team members or by text message (using a pre-worded message) at their discretion. If there is no response (and a reasonable cause for concern) to this within a reasonable period of time, then the participant's 24-hour contact will be contacted to ensure the safety of the participant. In addition, participants will be telephoned in the morning if they are not due to be seen at the CCVTM for a follow up visit.

Participants may be asked the following questions during the telephone conversation:

- Have they had a temperature ≥38.0°C?

[vi] Samples not meeting these criteria should be cultured qualitatively only.

- Have they had any symptoms, and if so their severity?
- Have they taken their antibiotics (if prescribed)?
- Have they any other concerns?

24-hour physician contact

Participants will have access to a study physician 24-hours per day, from the time of vaccination until they are deemed to be clear of *Salmonella* Typhi infection (see section 0). Following challenge, participants will be encouraged to contact one of the study investigators on the 24-hour emergency telephone number if they develop symptoms of typhoid between the regular reviews, or when their temperature exceeds 38°C. The investigators will consider extra clinical reviews if the participants symptoms are moderate or severe, or at their request. Severity of signs and symptoms will be assessed by the tables outlined in section 0. If participants are unwell as a result of *S.* Typhi infection and unable to attend the CCVTM, they will be visited at home at least one of the clinical investigators/study physicians.

Days 21 to 3 years (V14-V21)

These visits will be performed within the following timelines from date of challenge:

- V14: day 21 +/- 2,
- V15: day 28 +/- 5,
- V16: day 60 +/- 10,
- V17: day 90 +/- 14,
- V18: day 180 +/- 21,
- V19: 1 year +/- 30 days,
- V20: 2 years +/- 42 days,
- V21: 3 years +/- 60 days,

At these visits the procedure performed will include the following:

- Draw blood as per **Table 2**.
- Collect saliva samples for immunological investigations (V14-18 only).
- Collect stool specimens for immunological, bacteriological and inflammatory investigations.
- Obtain midstream urine for mass spectrometry (20mL).

Blood sampling

During the study blood will be drawn according to the schedule shown in **Table 2**, by individuals approved by the Chief Investigator using the procedure described in OVG SOP

009 version 7; *Taking venepuncture samples* and the OVG Clinical Study Plan. The number of attempts required to draw blood will be recorded in the participants CRF. If the first attempt is unsuccessful, further attempts will only be performed with the verbal agreement of the participant. Local anaesthetic spray (ethyl chloride spray) or creams (Ametop® (tetracaine gel, 4%), Emla® (lidocaine 2.5% with prilocaine 2.5% cream)) may be used at the request of the participant.

Participants fulfilling the definition of typhoid fever (see section 0) will have bloods drawn as per rows marked 'TD' (typhoid diagnosis) in **Table 2,** from the point of onset of typhoid fever for 4 days. The TD schedule will replace blood draws that would otherwise have been due. After 4 days, the schedule will revert back to the nearest applicable time point.

Samples taken from the point of typhoid challenge until the participant has been deemed to be clear *Salmonella* Typhi infection (section 0) will be transported with a 'Danger of Infection' alert sticker attached (see section 0).

6.1.1.1 **Additional notes regarding blood sampling**

Blood cultures:

- will be performed from day 5 after challenge. If the participant reports symptoms of concern, such as a headache, rash or sore throat, or records a temperature ≥37.5ºC before this point, then daily blood culture monitoring will be started sooner.
- Blood culture monitoring will cease once ≥1 culture is reported negative at 48 hours incubation if and after a diagnosis of typhoid fever has been made.[vii]

Haematology/biochemistry bloods:

- ESR (erythrocyte sedimentation rate) will be performed for baseline screening purposes only.
- Any tests still abnormal at day 14 will be repeated at subsequent visits to ensure return to normality.

Antibodies:

- The antibody sample taken at screening will be used for anti-endomysial and total IgA antibody testing by the ORH immunology laboratory. Samples to be processed by the ORH Trust are highlighted blue below.

[vii] By 48 hours incubation, a further 2 blood cultures will already have been received by the ORH microbiology laboratories; thus 3 cultures would be in process. If *S.* Typhi is subsequently cultured from any of these, further blood cultures would be performed as clinically indicated.

6.7.3 Obtaining participant's height and weight

To allow exploratory analysis on the effect of challenge dose per kilo of body weight, participants will be contacted by phone or email to ask them what their height and weight was at the time of challenge. Participants will also be measured and weighed at the CCVTM when attending their next available routine follow up visit if they verbally consent to do so.

Table 2: Blood test schedule

**Investigation**		Blood culture	PCR	Bacterial quantification	Full blood count	CRP	U+E	LFT	Antibodies	Serum bactericidal assay	Antibody secreting cells	Cell mediated immunity	ALS assay	Cytokines	Functional genomics	
**Sample tube**		aerobic BACTEC bottle	heparinised falcon (15mL)	Wampole™ Isostat® Isolator	EDTA vacutainer	Heparin vacutainer			Serum vacutainer	Serum vacutainer	heparinised falcon (50mL)	EDTA vacutainer	heparinised falcon (15mL)	Heparin vacutainer	Tempus™ Blood RNA	
**Volume (mL)**		10	5	10	1	2			5	3	25	variable	5	2	3	
**Visit**	**Time**															**TOTAL**
Screening: Within 90 days					3	2			5							10
Vaccination	Day -32 (Ty21a only)								5	3	25	75	5	2	3	118
Vaccination	Day -30															0
Vaccination	Day -28 (M01ZH09 /Placebo)								5	3	25	75	5	2	3	118
FG- 1	Day -30														3	3
FG- 2	Day -28														3	3
FG- 3	Day -26														3	3
FG- 4	Day -24														3	3
Va	Day -21								5	3	25			2	3	38
Vb	Day -14								5	3		35		2	3	48
1	Day 0				1	2			5	3		75	5	2	3	96
1	12hrs		5											2	3	10
2	24hrs		5						5	3				2	3	18
3	D3		5											2	3	10

Investigation		Blood culture	PCR	Bacterial quantification	Full blood count	CRP	U+E	LFT	Antibodies	Serum bactericidal assay	Antibody secreting cells	Cell mediated immunity	ALS assay	Cytokines	Functional genomics	
4	D5	10	5		1	2			5	3				2	3	31
**Visit**	**Time**															
5	D6	10	5											2	3	20
6	D7	10	5						5		25	30	5	2	3	85
7	D8	10	5		1		2								3	21
8	D9	10	5												3	18
9	D10	10	5		1	2			5	3			5	2	3	36
10	D11	10	5												3	18
11	D12	10	5		1	2									3	21
12	D13	10	5												3	18
13	D14	10	5		1		2		5	3		45	5	2	3	81
14	D21								5	3					3	11
15	D28								5	3		75	5	2	3	93
TD	hr0	10	5	10	1	2			5	3			5		3	44
TD+6	hr6	10	5												3	18
TD+12	hr12	10	5												3	18
TD+24	hr24	10	5		1	2									3	21
TD+48	hr48	10	5		1	2			5	3	25	40	5	2	3	101
TD+72	hr72	10	5												3	18

Investigation		Blood culture	PCR	Bacterial quantification	Full blood count	CRP	U+E	LFT	Antibodies	Serum bactericidal assay	Antibody secreting cells	Cell mediated immunity	ALS assay	Cytokines	Functional genomics	
**Visit**	**Time**															
TD+96	hr96	10	5		1	2			5	3		40	5	2	3	76
**Total in 28 days**		**If NO typhoid diagnosis**														**804**
		**If typhoid diagnosed at day 14 & FG subgroup (i.e. maximum volume)**														**1117**
16	D60								5	3		45		2	3	58
17	D90								5	3		45		2	3	58
18	D180								5	3		80		2	3	93
19	D365								5	3		80		2	3	93
**Total in 1 year**																**1419**
20	Yr2								5	3		80		10	3	101
21	Yr3								5	3		80		10	3	101
**Total in 3 years**																**1621**

7. MANAGEMENT OF PARTICIPANTS WITH TYPHOID FEVER

Definition of illness

Typhoid fever

For the purposes of analysing data relating to the primary endpoint and for notification of cases to the Health Protection Unit, typhoid fever will be defined as:

- A positive blood culture for *Salmonella* Typhi from Day 7 post-challenge

  OR,

- A positive blood culture for *Salmonella* Typhi before Day 7 post-challenge with objective signs/symptoms of typhoid infection (such as a recorded temperature ≥38°C)

  OR,

- Oral temperature ≥38°C, persisting continuously for at least 12-hours in the absence of anti-pyretic medication, occurring from 72-hours after challenge.

Salmonella Typhi bacteraemia occurring before Day 7 may reflect a primary bacteraemia and not 'true' typhoid fever; however participants who are bacteraemic before day 7 AND have clinical symptoms/signs consistent with typhoid infection (such as a temperature ≥38°C) will be also be deemed to have reached the definition for typhoid fever. Fever occurring before 72-hours is unlikely to be due to typhoid fever.

Microbiologically, the earliest indication of *Salmonella* Typhi bacteraemia will be identification of Gram-negative bacilli by Gram staining of aerobic blood/broth culture specimens. Formal identification of the organism as *Salmonella* Typhi, using a combination of routine serological and biochemical techniques, will take a minimum of a further 24-hours. Participants from whom Gram-negative bacilli are identified in the aerobic blood culture bottle, will therefore be defined as having typhoid fever for the purposes of clinical management (including antibiotic treatment) and for handling blood, urine, stool and saliva samples. To maximise the diagnostic yield obtained with this definition, aerobic bottles will be inoculated with 10mls of blood at each time-point.

Severe typhoid fever

Severe typhoid fever will be defined as above, with the addition of any ***ONE*** or more of the following:

- Oral temperature ≥ 40ºC,
- Systolic blood pressure ≤ 85 mmHg,
- Significant lethargy or confusion,
- Gastrointestinal bleeding or suspected/confirmed perforation,
- Any Grade 3 or above laboratory abnormality (see section 0).

Reporting to the Health Protection Unit

The Thames Valley Health Protection Unit will be informed of the name, address and date of birth of all participants who:

- fulfil the definition of typhoid fever,

  and / or,

- have *Salmonella* Typhi cultured from one or more faecal specimens.

In addition, any breaches in enteric precautions that result in another individual coming into contact with the excreta of a participant should be reported to the proper officer/ HPU.

Admission to inpatient facility

When the definition of typhoid fever is reached, each participant will be clinically evaluated by a study physician. If any of the following criteria are met, admission to the John Warin Ward (Infectious Diseases Unit, Oxford Radcliffe Hospitals NHS Trust) will be considered.

1. Severe typhoid fever (defined in section 0),
2. Failure of symptoms to improve within 72 hours of starting antibiotic therapy,
3. Inability to tolerate oral antibiotics,
4. Dehydration/hypotension requiring intravenous fluid therapy,
5. Unanticipated concern about participant's home circumstances.

In addition, any participant that deviates from the protocol and takes antipyretics at home before the definition of typhoid fever is reached will be treated with antibiotics and a decision made by the Chief Investigator and Consultant Physician regarding withdrawal from the study.

Ultimately the decision regarding admission will be taken by the investigators in conjunction with the Infectious Diseases Consultant on-call. Inpatient care will be under the care of the Infectious Diseases Consultant on-call. Study procedures and investigations as described in **Tables 1** and **2** may be performed by study staff where this does not interfere with clinical care. The Infectious Diseases Consultant On-call will be made aware of the study protocol

and the suggested treatments outlined below, but management of inpatients is at their discretion.

Blood sampling for participants with typhoid fever

For participants who develop typhoid fever, blood tests will be performed as per **Table 2**; rows labelled Typhoid Diagnosis (TD). This schedule replaces other scheduled bloods during these days. After 96 hours from reaching the definition of typhoid fever, samples should be obtained according to the next applicable post-challenge time-point.

Medication

All medication will be dispensed in accordance with the Clinical Study Plan in accordance with the indications and dosages given in the British National Formulary [80]. All study medication will be stored on-site at the Centre for Clinical Vaccinology and Tropical Medicine.

Antipyretics and analgesics

Paracetamol 0.5 to 1g, every 4 to 6 hours/PRN, PO/PR, maximum 4g daily, will be prescribed to relieve symptoms and control temperature, if needed, after the definition of typhoid fever has been met.

If participants require alternative analgesia, **Codeine Phosphate 30mg-60mg,** every 4 hours/PRN, PO, max. 240mg daily can be prescribed.

Diarrhoea

Oral rehydration will be given to replace diarrhoeal output.

Constipation

Senna 2 to 4 tablets, PO, max. BD, will be prescribed to relieve constipation.

Nausea and vomiting

Domperidone 10 to 20mg, 3 to 4 times daily/PRN, PO, max. QDS or 80mg daily, will be prescribed to relieve nausea and vomiting.

Allergy

Chlorpheniramine 4mg, every 4 to 6 hours/PRN, PO, max. 24mg daily, will be prescribed for any participant experiencing a mild (as determined by a study doctor) allergic reaction.

Anaphylaxis will be immediately managed in keeping with OVG SOPs and referred to the Infectious Diseases Consultant for further management.

Antibiotics

See below (section 0).

Antibiotic treatment

A treatment course of **Ciprofloxacin, 500mg BD,** PO, will be given for 14 days to:

- Any participant developing typhoid fever (section 0),
- Any participant with 3 or more of the following symptoms severe enough to interfere with all normal activity after challenge:
  - Malaise
  - Headache
  - Myalgia/ Arthralgia
  - Anorexia/ Loss of appetite
  - Nausea/ Vomiting
  - Constipation/ Diarrhoea
  - Abdominal pain
  - Cough
  - Rash,
- Any participant in whom Gram-negative bacilli are identified by Gram staining of blood culture smear,
- Any participant who has not received antibiotics by Day 14 (V13),
- Any participant in whom antibiotic use is felt to be clinically necessary (as determined by a study physician).

Ciprofloxacin is a licensed antibiotic and is a first-line treatment for *S.* Typhi infection. Participants will be asked to take one dose per day in the presence of a study investigator, or, if the participant is not due to visit the CCVTM when an antibiotic dose is due, they will be contacted to remind them to take the antibiotic dose.

Ciprofloxacin is contraindicated in pregnancy. A pregnancy test will therefore be performed in female participants of childbearing potential prior to treatment; an alternative antibiotic will used if necessary (see list below).

Absorption of ciprofloxacin is decreased by antacids and iron supplements. Participants will be counselled not to take these during the 14-day antibiotic course.

Any participant in whom a contra-indication to ciprofloxacin becomes apparent (as per the British National Formulary),[80] and who have not taken sufficient antibiotics to treat infection (i.e., < 7 days or as determined by the Chief Investigator or consultant physician) the following regimens of licensed antibiotics will be used:

- 2nd line: **oral azithromycin 500mg** OD for 14 days,
- 3rd line: **oral amoxicillin 500 mg** TDS daily for 14 days,

- 4th line: **oral trimethoprim/sulfamethoxazole (Septrin) 160/800mg** for 14 days.

Any antibiotic may reduce the efficacy of hormonal contraceptives. Female participants will be advised to use additional barrier contraception for the duration of the antibiotic course and for 7 days subsequently, over and above the advice given as to the importance of not becoming pregnant one month before/during vaccination and subsequent challenge, until deemed clear of infection (sections 0 and 0). A participant's general practitioner will be notified in writing of the antibiotics received.

Clearance of infection

To exclude chronic carriage of *S.* Typhi in the gallbladder, microbiological culture of stool samples will be performed weekly starting 3 weeks after completion of the antibiotic course (using the method described in section 0).[85] Participants will be **deemed clear of infection** after 2 successive negative cultures (or 3 for health and social care workers). Once this criterion is satisfied the participant will considered to be fully treated for *Salmonella* Typhi infection and to no longer pose an infection risk.

If 2 or more successive stool specimens remain culture-positive for *Salmonella* Typhi (which would occur a minimum of 4 weeks after completion of antibiotics), then the participant will be referred to an Infectious Diseases Consultant for further management.

The proper officer/HPU will be informed of all participants in whom clearance has been demonstrated and of any participant who fails to demonstrate clearance after the initial 14 day course of antibiotics or after any other antibiotic treatment.

The employer of any participant involved in the provision of health or social care to vulnerable groups will be notified in writing once 3 successive samples are negative.

Screening of close contacts for carriage of *S.* Typhi

Close or household contacts will receive letters from the study team via the participant, offering the opportunity to be screened for *S.* Typhi infection. This would involve supplying their demographic details and 2 stool samples a minimum of 48-hours apart; and a minimum of 7 days after the participant with whom they have been in contact has begun antibiotic treatment. If either sample is *S.* Typhi culture-positive, he/she will be referred to an Infectious Diseases Consultant for appropriate antibiotic management, and the proper officer /HPU will be informed.

Transport of samples

All samples from volunteers must be a labelled with a 'Danger of Infection' sticker. If a specimen sample bag is to be used, this should also be labelled 'Danger of Infection'. Samples should be transported in accordance with local SOPs.

LABORATORY METHODS

Blinding of laboratory samples

Details regarding the process of blinding laboratory samples are given in the OVG Clinical and Laboratory Study Plans. Samples processed by departments at the Oxford Radcliffe Hospitals will be processed using the participant study number, date of birth and gender only.

Bacteriology

Blood culture

After inoculation of aerobic broth with 10mls of the participants blood (*BACTEC* PLUS Aerobic/F culture vial; BD, Oxford, UK), culture will be performed using the BACTEC 9240 continuous monitoring system in the microbiology laboratory of the Oxford Radcliffe Hospitals NHS Trust, according to the current version of M-SOP-017 Blood Culture. Identification of organisms cultured will be by biochemical (API, Analytical Profile Index; bioMérieux, Basingstoke, UK) and serological methods, latterly by agglutination with *Salmonella* Typhi anti-sera. Isolates will be tested for antibiotic sensitivity to ciprofloxacin, nalidixic acid, trimethoprim and ampicillin. Blood cultures obtained out-of–hours will be transported to the microbiology lab as soon as possible.

Quantitative culture of whole blood will be performed to determine the number of organisms in the blood, using the Wampole™ Isostat® Isolator system (Oxoid Ltd, Basingstoke). Enumeration of *Salmonella* Typhi organisms in the blood will be performed by lysis centrifugation followed by direct plating onto non-selective media.

Stool culture

Stool samples (and rectal swabs) supplied by participants should be delivered to CCVTM within 1 hour of being taken or as soon as possible thereafter. If possible, the samples should be kept cool until delivered to the CCVTM/OVG and then stored at 2-8°C. Samples not meeting these criteria should be cultured qualitatively only. The time of sampling will be noted by the participant on the sample form. The time of refrigeration should be recorded by a study investigator on the sample form.

Routine stool (and rectal swab) cultures and screening for enteric pathogens will be performed by the microbiology laboratory, ORH, according to the current version of M-SOP-111 Stool Culture. Stool will be inoculated directly onto XLD agar for semi-quantitative culture and into Selenite F enrichment broth for qualitative culture. After overnight incubation (at 37ºC), each sample will be sub-cultured onto Salmonella-selective chromogenic agar (SALM

agar, E&O Laboratories Ltd, Bonnybridge, Scotland). Suspicious colonies will be identified as per the blood culture method described above.

Isolates of *S.* Typhi may be retained for phage typing by the reference laboratory if challenge strain confirmation is required by the HPU.

Additionally, quantitative cultures will be performed on all stool specimens by qualified members of the OVG study team. Stool samples from participants who shed the challenge organism may be frozen for possible future phage typing.

In a sub-group of participants, stool will be used for analysis of the microbiome. Stool will be stabilised with RNA-later and stored at -80°C. Samples will be analysed using mass-spectrometry to determine the microbiome.

Blood PCR detection

The Oxford Vaccine Group has developed a fast and highly sensitive novel TSB-bile blood culture-PCR assay and will use this to detect low levels of *Salmonella* Typhi in the blood of participants after challenge.

Briefly, 5mls whole blood are pre-treated with micrococcal nuclease prior to inoculation into 15ml tryptone soya broth (TSB) containing 3.0% ox bile; giving a final concentration of 2.4% ox bile and 20% blood. The blood/broth culture is shaken at 200rpm in a 37°C incubator for up to 5 hr, and then centrifuged at 5,000rpm for 20 minutes. The supernatant is then discarded and the pellet used for DNA isolation. DNA will be isolated using the UltraClean® BloodSpin® kit (MO BIO Laboratories Inc., CA, USA) according to the manufacturer's instructions. Isolated DNA will be used as a template for organism detection by PCR amplification, utilising the *S.* Typhi fliC-d gene employing primers H-for (ACTCAGGCTTCCCGTAACGC) and Hd-rev (GGCTAGTATTGTCCTTATCGG).

Immunology

Inflammatory responses

7.1.1.1 **Plasma cytokines**

The kinetics of the inflammatory response will be measured by assay of stored plasma samples for levels of cytokines including IL-1β, IL-2, IL-4, IL-5, IL-6, IL-8, IL-10, IL-12, TNF-α and IFN-γ. Plasma samples will be isolated from blood and stored at -70°C for assay later. A commercial multiplex bead-array kit (Human Th1/Th2 11plex FlowCytomix Multiplex, BMS810FF, eBioscience, Ltd, Hatfield, UK) will be used for the cytokine assay. The assay will be carried out by qualified members of the OVG study team in the OVG Laboratory.

7.1.1.2 **Faecal lactoferrin**

Stool samples will be assayed for lactoferrin, which is a marker of gastrointestinal tract inflammation. Samples provided by participants will be stored as described above prior to refrigeration (2-8°C) for up to 7 days or immediate freezing for long-term storage. The time of sampling will be noted by the participant/investigator on the sample form. Faecal lactoferrin will be measured using a commercially available quantitative ELISA kit by qualified members of the OVG study team in the OVG Laboratory.

Antibody responses

7.1.1.3 **Serum**

Serum samples will be tested for IgG, IgM, and IgA antibodies to *Salmonella* Typhi O, H, and Vi antigens measured by ELISA. Serum will be isolated from blood and stored at -70°C prior to assays being performed. Analysis of antibodies to *Salmonella* Typhi O, H, and Vi antigens will be carried out by qualified members of the OVG study team in the OVG Laboratory.

H antibody will also be measured by Widal tube agglutination using *S.* Virginia as antigen (*S.* Virginia has the same flagellar antigen as *S.* Typhi). The antibody response will also be tested in a whole cell ELISA and functional activity of antibodies will be tested using a serum bactericidal assay.

7.1.1.4 **Supernatant**

Antibody-in-lymphocyte supernatant assays will be performed at baseline, typhoid diagnosis, 24 and 96 hours after typhoid diagnosis and days 14 and 28 after challenge. Briefly, isolated PBMCs will be cultured overnight with antigen preparations (including LPS and whole cells) before the supernatants are removed and measured for concentrations of *S.* Typhi specific IgM, IgA, IgG antibodies by ELISA.

Mucosal immune responses

Gut-derived, trafficking, antibody-secreting cells (ASC) that secrete IgA or IgG antibody against *Salmonella* Typhi O, H, or Vi antigen will be measured by using both ELISA and ELISPOT [66]. Briefly, peripheral blood mononuclear cells (PBMC) will be separated by Lymphoprep gradient centrifugation and added to antigen-coated ELISPOT plates. In the ELISPOT, specific IgA or IgG secreted by individual ASCs will be detected by counting coloured spots produced by reaction of the substrate with bound anti-human IgA conjugate. Samples collected after day 30 will be polyclonally stimulated *in vitro* to detect memory B-cells.

Total salivary IgA will be measured using ELISA. Saliva will be collected using a sponge device inside the mouth, from the mucosa inside the cheek (buccal). The saliva will be transferred from the sponge device into a tube by centrifugation.

These assays, which have been well developed and widely used in our laboratory, will all be carried out by qualified members of the OVG study team in the OVG Laboratory.

7.1.1.5 ***S.* Typhi-specific coproantibodies**

IgG and IgA and specific IgG and IgA antibodies against *Salmonella* Typhi LPS O antigen, H antigen, and Vi antigen will be measured in stool samples provided by participants and stored as per the faecal lactoferrin assay, described above. If specific IgA is detected, IgA1 and IgA2 subclass determination will be performed.

Briefly, the stool specimen will be suspended in a 10% solution of supplemented PBS, centrifuged, and the supernatant assayed for antibody by ELISA. *Salmonella* Typhi-specific coproantibodies will be measured by qualified members of the OVG study team in the OVG Laboratory. These assays have been developed and are widely used in our laboratory.

Cellular immune responses

Cellular immune responses will be analyzed in collaboration with the University of Maryland. Peripheral blood mononuclear cells (PBMC) will be separated by Lymphoprep gradient centrifugation and frozen-stored in liquid N_2 until shipped to the University of Maryland using dry liquid N_2 shippers. PBMCs will be used for assays including:

- **Specific cytokine production in response to soluble *Salmonella* Typhi antigens (e.g., flagella, OmpC, GroEL) and *Salmonella* Typhi-infected autologous or 721.221.AEH targets:** a flow cytometric-based BD Cytometry Bead Array and/or ELISA will be used for measuring levels in supernatants, and multi-chromatic flow cytometry and/or ELIPSOT used for intracellular cytokine measurement. Cytokines measured will include IFN-γ, TNF-α, IL-2, IL-4, IL-5, IL-10 and IL-12. Multifunctional cytokine production by T cell subsets will be examined by flow-cytometry.[66,89]
- **Induction, persistence and homing of multifunctional specific memory T cell subsets** (e.g., T effector/memory, T_{EM}; T central/memory, T_{CM}; CD45RA$^+$ T_{EM}, T_{EMRA}) able to secrete IFN-γ, TNF-α and/or IL-2 will be measured by flow-cytometry.[66]
- **Cytotoxic T cell activity (CTL) against *Salmonella* Typhi-infected targets**, including assays examining **(1)** autologous targets for classical class-Ia-restricted CTL and **(2)** the 721.221.AEH cell line for class-Ib HLA-E-restricted CTL.[66,89]
- **Induction, persistence and homing of specific memory B cells to S. Typhi antigens** (e.g., LPS, flagella, others); these studies will include an in-depth

characterization of the B_M cells by flow cytometry and functional assays using modified IgG and IgA ELISPOT assays.[64]

Functional genomics

Functional genomics will be performed by qualified members of the OVG study team in the OVG Laboratory and/or The Sanger Centre, Cambridge or elsewhere. Briefly, Tempus™ Blood RNA Tubes (Applied Biosystems, Warrington, UK) will be inoculated with blood and processed immediately or stored at room temperature for up to 5 days or at 4°C for up to 7 days, or at -20°C indefinitely. RNA will be isolated using Tempus™ Spin RNA Isolation Kit (Applied Biosystems) and used for study of gene expression profiles. Gene expression profiles will be determined using commercially available gene expression microarrays and/or mRNA-seq technologies.

Mass spectrometry

Urine mass spectrometry will be carried out in collaboration with Imperial College London Medical School. Mid-stream urine will be analysed by SELDI-TOF mass spectrometry according to established protocols.[90] Urine samples will be collected and stored at -80°C until shipped to Imperial College London.

Briefly, mid-stream urine samples will be collected and stored on ice prior to transfer to the OVG Laboratory. Samples will be centrifuged for 10 minutes at 13000g (to remove cellular debris) and subsequently stored at -20°C overnight, prior to transfer to -80°C.

Before analysis, urine will be pre-treated with ProteoMiner beads (BioRad) or placed directly on the surface of ProteinChips with different surface chemistries including CM10 (weak cation exchange), Q10 (strong anionic exchange), H50 (hydrophobic), NP20 (general protein binding) or IMAC (immobilised metal affinity chromatography). After washing with buffers, appropriate to the ProteinChip being used, energy absorbent matrix will be added and mass spectrometric profiles determined in the SELDI Personal Edition ProteinChip® Reader (BioRad, CA, USA). Peaks of interest will be identified at the molecular level using the BioRad Lucid system comprising a SELDI-tandem mass spectrometer combination instrument

Other laboratory investigations

All other laboratory tests including WBC, differential counts, C-reactive protein, urea, creatinine, electrolytes, AST, ALT, alkaline phosphatase, bilirubin, amylase will be performed using the ORH, NHS laboratories. Briefly, blood samples will be collected in assay sample tubes and delivered to ORH clinical laboratories for analysis according to national SOPs.

Samples collected as part of this trial may also be used for other exploratory studies of scientific relevance by the OVG Laboratory or any of the collaborating laboratories. These samples will be material not deemed 'relevant' under the HTA (see IRAS section B). Studies may include further investigation of the inflammatory and immunological response to vaccination and/or challenge.

Participant questionnaire

Following completion of the 60 day visit, participants will be emailed details of an on-line questionnaire regarding their experience of the study. Participants who do not have an email address, access to the internet, or express a preference for a paper copy will be sent a letter together with an accompanying printed questionnaire.

8. DEFINITION OF END-OF-STUDY

The end-of-study is completion of the last laboratory assay on the last participant sample.

Additional rules for early termination or suspension of the study are provided in the DMSC charter.

9. SOURCE DATA

Source documents are original documents, data and records from which participants' CRF data are obtained. These include, but are not limited to, hospital records (from which medical history, both previous and subsequently generated, may be summarised into the CRF), laboratory result forms, radiology result forms, clinical charts, laboratory and pharmacy records, diaries, and correspondence.

CRF entries will be considered source data if the CRF is the site of the original recording (i.e., there is no other written or electronic record of data).

All documents will be stored safely in confidence. On all study-specific documents, other than the signed consent, contact information and screening sheets, the participant will be referred to by the study participant number only, not by name or other identifying feature.

10. TREATMENT OF PARTICIPANTS

Description of study vaccines

M01ZH09 vaccine

The active ingredient is live attenuated *Salmonella* Typhi, strain *S.* Typhi (Ty2 *aroC⁻ ssaV⁻*) ZH9, which is given with a sodium bicarbonate solution (described below) in a single oral dose.[60]

S. Typhi (Ty2 *aroC⁻ ssaV⁻*) ZH9 contains two independently attenuating gene mutations; one, a 600 base pair deletion in the aroC gene and the second, a 1893 base pair deletion in the ssaV gene. The strain is produced by batch fermentation using broth supplemented with glucose and aromatic compounds. At the end of the fermentation process a concentrated suspension of the strain is formulated (in a basal medium, M9S plus 10% sucrose) and freeze-dried. The dose to be given is $1x10^{10}$ cfu which will be prepared from supplied vials containing $0.2\text{-}1.7x10^{10}$ cfu/vial. The contents of up to 5 vials will be reconstituted in sodium bicarbonate solution and pooled; the volume calculated to contain $1x10^{10}$ cfu (based on the most recent vaccine stability data provided by Emergent BioSolutions) will then be removed and added to the bicarbonate solution to be given to the participant. The constituents of the M9S basal medium plus 10 (w/v) sucrose are:

- Soya peptone,
- $Na_2HPO_4 \cdot 12H_2O$ (disodium hydrogen phosphate),
- KH_2PO_4 (potassium dihydrogen phosphate),
- NaCl (sodium chloride),
- NH_4Cl (ammonium chloride),
- $MgSO_4 \cdot 7H_2O$ (magnesium sulphate heptahydrate),
- $CaCl_2 \cdot 2H_2O$ (calcium chloride),
- Sucrose.

Vaccine placebo (for M01ZH09)

The placebo vaccine consists of M9S basal medium plus 10% sucrose, as described above. It is supplied as a freeze-dried cake in glass vials and is identical in appearance to the M01ZH09 vaccine. It is administered with bicarbonate in the same manner as the active vaccine.

Ty21a vaccine

This vaccine is a licensed, live attenuated, oral vaccine. Ty21a will be given as three doses two days apart as per the summary of product characteristics.[91] It is supplied in the form of enteric-coated gelatin capsules packaged in foil blister packs. Each capsule contains not less than 2 x 10^9 viable cells of *Salmonella enterica* serovar Typhi strain Ty21a.

Administration of vaccines and placebo

The investigator will be responsible for the administration of the vaccine to subjects enrolled into the study according to the procedures stipulated in this study protocol. Study vaccines should not be administered to individuals with known hypersensitivity to any component of the vaccine.

M01ZH09 and vaccine placebo

M01ZH09 is supplied as a freeze-dried cake in 6ml glass vials with rubber stoppers. The vaccine is administered with sodium bicarbonate, provided as a tablet, to neutralise stomach acid. The constituents of the bicarbonate tablet are:

- Sodium bicarbonate 2600mg
- Ascorbic acid 1650mg
- Aspartame 30mg.

The contents of the vial are prepared for administration by reconstitution and dilution immediately prior to oral administration.

To reconstitute M01ZH09 vaccine or placebo:

- Measure 150ml of drinking water in a graduated measuring cylinder.
- Pour the drinking water into beaker, with lid (minimum capacity 200ml).
- Add one bicarbonate tablet to the beaker, replace the lid and allow to dissolve.
- Swirl the beaker for 20-30 seconds to ensure mixing.
- Record the time at which the bicarbonate solution preparation is completed.
- Remove the plastic cap, foil seal and rubber stopper from the required number of vial(s) containing vaccine or placebo.
- Remove 1mls from the prepared bicarbonate solution in the beaker using a syringe.
- Add the 1mls of bicarbonate to a vial containing either the vaccine or placebo.
- Swirl gently to mix – try to ensure vaccine not dispersed up sides of vial.

- Repeat for each vial of vaccine or placebo.
- As per the dosing instructions, add 1 mL from each of the vaccine vials to a single container and remove the volume required to deliver a $1x10^{10}$ cfu dose.

  [The number of vials required and final volume containing $1x10^{10}$ cfu will be determined by the viable cell count of the clinical batch prior to dosing as reported by Emergent BioSolutions CMO.]

  Repeat for the placebo vials.
- Add the specified volume to the beaker containing the bicarbonate, replace lid and swirl to mix.
- Record time that preparation of the vaccine is completed.

Participants will be instructed to fast for one hour prior to administration of the vaccine/placebo. Bicarbonate solution and vaccine solution must be administered within 30 minutes of preparation. Following administration of the vaccine, participants will be instructed to fast for a further one hour, however, clear fluids will be permitted. Further details are provided in the OVG Clinical Study Plan.

Ty21a vaccine

Ty21a is supplied as capsules in a foil seal. Each capsule should be taken by the participant approximately one hour before a meal. Capsules should be swallowed with a cold or lukewarm drink (temperature not exceeding 37°C) on alternate days, i.e. taken on days 1, 3 and 5. Capsules should be swallowed whole and not chewed and as soon as possible after placing in the mouth.

Storage of study vaccines

M01ZH09/placebo will be supplied by Emergent BioSolutions after packaging and re-labelling by Aptuit Ltd (Deeside, UK), in participant kits containing 2 vials of MH01ZH09 or placebo and one blister pack containing 2 bicarbonate tablets.

The Ty21a vaccine will be purchased directly from Crucell UK Ltd (Bradford, UK) by the Oxford Vaccine Group, and will have additional minimal labelling attached as required by EU GMP guidelines.[92]

All vaccines, placebo, bicarbonate tablets and cartons will be labelled with no less than the study name/code, the chief investigator name, vial number and ‘for clinical trial use only’ and other local relevant regulatory requirements. The investigator (or delegate) will make an inventory and acknowledge receipt of all shipments of study vaccine.

Vaccine supplies will be transported to the OVG in containers refrigerated at 2°C to 8°C. All vaccine supplies must be stored between +2 and +8°C within the OVG vaccine fridge at the CCVTM in accordance with the manufacturers' instructions and OVG SOP 002 version 9; *Vaccine Storage*.

Compliance with vaccine dosing regimens

All doses of vaccines given in this study will be administered by study investigators on site at the CCVTM, recorded in the CRF and verified by a second team member. The study medication will be at no time in the possession of the subject and compliance will not therefore be at issue.

Accountability for the study vaccines

The M01ZH09 vaccine and placebo will be supplied by Emergent BioSolutions; Ty21a, manufactured by Crucell UK Ltd, will be sourced by the Oxford Vaccine Group from the manufacturer. All vaccines will be received in accordance with OVG SOP 001 version 3; *Vaccine Receipt, Cold Chain Maintenance and Return/Disposal*, and all vaccine doses will be accounted for within an accountability log. Unused vaccine at the end of the trial will be disposed of or returned to Emergent BioSolutions with written documentation describing this process. Any recall of study vaccines required for use in the study or reporting of defective vaccines will be performed according to the OVG SOPs 054 version 1; *Vaccine Product Recall Procedures*, and 055 version 1; *Vaccine Defect Reporting Procedures*, respectively.

***S.* Typhi challenge strain**

GMP manufacture

Three dose levels of the *Salmonella* Typhi (Quailes strain) were originally supplied by the Health Protection Agency (Porton Down, Salisbury, UK) after manufacture to GMP standards (see **Appendix 1**). Using the results of the previously performed dose-finding study [75], the required dose will be administered (after necessary adjustment) to provide an inoculum of 1-5x10^{4} CFU.

Storage

The *Salmonella* Typhi (Quailes strain) for inoculation of participants will be stored as a frozen suspension in soya tryptone medium containing 10% sucrose. Suspensions will be labelled with no less than the contents, vial number and manufacturing date. Following GMP manufacture, the *S.* Typhi (Quailes strain) challenge agent will be shipped by the Health Protection Agency using an accredited courier to the Oxford Vaccine Group Laboratory for storage.

Vials of the required concentration of *Salmonella* Typhi (Quailes stain) will be thawed and diluted immediately prior to use.

Accountability for the challenge strain

The investigator will be responsible for adequate and accurate accounting of *Salmonella* Typhi vials prepared for administration to participants. The investigator or designee will administer the study *Salmonella* Typhi vials only to individuals included in this study following the procedures set out in this study protocol and the associated OVG Clinical and Laboratory Study Plans. The date, dosage and time of administration will be recorded.

The investigator will track all vials of *Salmonella* Typhi received, used, administered to participants and wasted within an accountability log. Unused vials will be stored for further use until expiry. Any vials which have been thawed will be destroyed.

Concomitant Medication

Any medication, including 'over-the-counter' and herbal products taken within 4 weeks prior to screening and during the study (to day 28), will be recorded on the CRF. Medication outlined in sections 0 and 0 will be prescribed by study investigators. If a participant requires antibiotic therapy before the planned date of vaccination or is expected to require antibiotics within 14 days following vaccination, vaccination should be delayed until 14 days has elapsed. If a participant requires antimicrobial therapy within 14 days following vaccination then assessment will be made by a study investigator as to whether a participant should be withdrawn or excluded from a per protocol analysis; this will depend on the timing in relation to vaccination, dose, indication and type of antimicrobial used. Participants should not receive any vaccine other than the study vaccine in the 4 weeks prior to dosing or four weeks after challenge.

Female participants using oral hormonal contraception should be advised to use additional barrier contraception if diarrhoea occurs as an adverse event post-vaccination.

Discontinuation/withdrawal from study at any stage

Each participant has the right to withdraw from the study at any time. In addition, the Chief Investigator and/or Data Safety Monitoring Committee may discontinue a participant from the study at any time if they consider it necessary for any reason. These may include:

- Vomiting within 1 hour of administration of either vaccines or placebo.
- Treatment with antibiotics in the 7 days after completion of the vaccine course.
- Vomiting within 90 minutes of ingestion of *S.* Typhi challenge.
- Pregnancy.

- Ineligibility (either arising during the study or retrospective having been overlooked at screening).
- Significant protocol deviation.
- Significant non-compliance with treatment regimen or study requirements.
- An adverse event that results in inability to continue to comply with study procedures.
- Consent withdrawn.
- Loss to follow up.

Withdrawal from the study will not result in exclusion of data already gathered. Participants will be replaced if the diagnosis of typhoid has not been reached by the time of withdrawal. If the participant is withdrawn due to an adverse event, the investigator will arrange for follow-up visits or telephone calls until the adverse event has resolved or stabilised. The reason for all withdrawals/discontinuations will be recorded in the CRF.

Additional safety measures for discontinuation/withdrawal after challenge

Any participant withdrawing from the study after receiving the challenge agent will be given a 14-day course of antibiotics and additional safety visits may be made as required. This will be made clear during the consent-taking process. In addition, a requirement will be made for 2 stool samples taken 7 days apart to be collected 3 weeks after stopping antibiotics to ensure that the participant has not developed chronic carriage of the *S*. Typhi challenge strain. The details of any participant that defaults from treatment or visits or who fails to provide the required stool samples will be given to the proper officer/Health Protection Unit.

11. SAFETY REPORTING

Definitions

Following advice from the MHRA, we have been assured that the challenge agent does not constitute an IMP. The M01ZH09 vaccine, associated placebo and the Ty21a positive control will constitute an IMP, placebo and comparator, respectively. The following definitions are commensurate with the OVG SOP024 version 4; *Reporting of SAEs, SUSARS and Periodic Safety Reports*.

Adverse Event (AE)

An AE or adverse experience is:

> Any untoward medical occurrence in participants after administration of a medicinal product, which does not necessarily have to have a causal.

An AE can therefore be any unfavourable and unintended sign (including an abnormal laboratory finding), symptom or disease temporally associated with the ingestion of a study vaccine or study medication, whether or not considered related to this.

Adverse Reaction (AR)

All untoward and unintended responses to a medicinal product related to any vaccine dose.

The phrases "responses to a medicinal product" means that a causal relationship between a study medication and an AE is at least a reasonable possibility, i.e. the relationship cannot be ruled out.

All cases judged by either the reporting medically qualified professional or the sponsor as having a reasonable suspected causal relationship to study medication qualify as adverse reactions.

Serious Adverse Events

To ensure no confusion or misunderstanding of the difference between the terms "serious" and "severe", which are not synonymous, the following note of clarification is provided:

The term "severe" is often used to describe the intensity (severity) of a specific event (as in mild, moderate, or severe myocardial infarction); the event itself, however, may be of relatively minor medical significance (such as severe headache). This is not the same as "serious," which is based on patient/event outcome or action criteria usually associated with events that pose a threat to a participant's life or functioning. Seriousness (not severity) serves as a guide for defining regulatory reporting obligations.

A serious adverse event is an AE that results in any of the following outcomes, whether or not considered related to study medication.

- Death
- Life-threatening event (NB: The term "life-threatening" in the definition of "serious" refers to an event in which the participant was at risk of death at the time of the event; it does not refer to an event which hypothetically might have caused death if it were more severe)
- Hospitalisation, regardless of length of stay, even if it is a precautionary measure for continued observation. Hospitalisation (including inpatient or outpatient hospitalisation for an elective procedure) for a pre-existing condition that has not worsened unexpectedly does not constitute a serious AE
- Results in persistent or significant disability/incapacity
- Consists of a congenital anomaly/birth defect.
- Other important medical events (that may not cause death, be life-threatening, or require hospitalisation that may, based upon appropriate medical judgement, jeopardise the participant and/or require medical or surgical intervention to present one of the outcomes listed above).

Serious Adverse Reaction (SAR)

An adverse event (expected or unexpected) that is both serious and, in the opinion of the reporting investigator, believed with reasonable probability to be due to study treatment, based on the information provided.

Suspected Unexpected Serious Adverse Reaction (SUSAR)

A SUSAR is defined as a serious adverse reaction, the nature or severity of which is not consistent with the applicable medicinal product information.

Medically significant event

The following events will be considered medically significant events:

- Severe typhoid fever (as defined in section 0),
- Failure to clinically or microbiologically cure a participant of typhoid fever after 14 days of antibiotic therapy,
- Transmission of *S.* Typhi to a contact of a participant,

- AEs requiring an additional physician visit or Emergency Department visit (with the exclusion of pre-planned visits and GP or Emergency Department visits for routine medical care),
- AEs leading to a participant's withdrawal.

Reporting procedure for all Adverse Events

In general, AEs will be reported using the following guidance. Specific procedures for reporting AEs after challenge are given in the relevant section below (0).

- All AEs occurring from the point of vaccination and for 7 days after vaccination and for 21 days post-challenge, either observed by the investigator or reported by the participant, whether or not attributed to study medication, will be recorded in the CRF (either in the Diary Card or other AE pages). Events starting within these time periods but persisting will be similarly recorded in the CRF.
- From Day 8 post-vaccination to challenge and between Days 22 and 90, medically significant adverse events will be recorded in the CRF, whether or not these are attributed to vaccine or *S.* Typhi ingestion or study medication.
- Pre-existing medical conditions (present before start of the AE collection period) are considered "concurrent medical conditions" and should not be recorded as AEs. However, if the participant experiences a worsening or complication of such a condition, the worsening or complication should be recorded as an AE. Investigators should ensure that the AE term recorded captures the change in the condition (e.g., "worsening of").
- Each AE should be recorded to represent a **single diagnosis**. Accompanying signs or symptoms (including abnormal laboratory values) should NOT be recorded as additional AEs. All AEs (vaccine, study medication and challenge related) should be recorded in the participants CRF.
- Changes in laboratory values are only considered to be AEs if they are judged to be clinically significant, e.g., if some action or intervention is required. If abnormal laboratory values are the result of pathology for which there is an overall diagnosis (e.g., decrease haemoglobin in gastrointestinal bleeding), the diagnosis only should be reported as one AE.
- All AEs resulting in participant withdrawal from the study or that are present at the end of the 90 day post-challenge period, will be followed up until a satisfactory resolution occurs or until a non-study related causality is assigned. It will be left to the investigator's clinical judgment whether or not an AE is of sufficient severity to require

the participant's removal from treatment. A participant may also voluntarily withdraw from treatment due to what he or she perceives as an intolerable AE. If either of these occurs, the participant must undergo an end-of-study assessment and be given appropriate care under medical supervision until symptoms cease or the condition becomes stable.

- Any pregnancy occurring during the clinical study and the outcome of the pregnancy should be recorded and followed up for congenital abnormality or birth defect.

The following AE-related information will be recorded: description, date of onset and end date, severity, assessment of relatedness to study medication or challenge with *S.* Typhi (as judged by a medically qualified investigator), other suspect drug or device and action taken. Follow-up information should be provided as necessary.

Vaccination-related AEs

In the period from the first dose of vaccine being given and for 7 days, the following solicited symptoms will be recorded once daily by the participants in a study diary:

- Malaise
- Headache
- Myalgia/ Arthralgia
- Anorexia/ Loss of appetite
- Nausea/ Vomiting
- Flatulence
- Constipation/ Diarrhoea
- Abdominal pain
- Cough
- Rash
- Fever (oral temperature)

In addition participants will be requested to record any other symptoms also in the diary card.

Diary cards will be reviewed by a study investigator at the vaccination follow up visit, Va. If clarification of any adverse event is required then the study investigator will seek this from the participant.

All vaccine-related AEs will be notified to Emergent BioSolutions within 7 days (see section 0).

Challenge-related AEs

Unfavourable signs and symptoms consistent with typhoid infection are to be expected following challenge, the most common of which are listed below. In the 21 days post challenge these solicited symptoms will be recorded once daily by the participant in a study diary:

- Malaise
- Headache
- Constipation/ Diarrhoea
- Abdominal pain

- Myalgia/ Arthralgia
- Anorexia/ Loss of appetite
- Nausea/ Vomiting
- Flatulence
- Cough
- Rash
- Fever (oral temperature)

In addition participants will be asked to record any other symptoms occurring within the 21 days post-challenge in their diary cards.

Challenge-related AEs that fulfil the definition for serious (as above) will be reported to the DSMC. Serious or severe complications arising as a **direct** result of challenge may be notified to the MHRA at the discretion of the Chief Investigator or Study Sponsor. Any unexpected events will be considered as possible adverse reactions to the IMP and reported as such (see vaccine-related AEs above).

Abnormalities in laboratory investigations will be recorded in the CRF by the study investigator.

Causality assessment

Causality assessment will follow OVG guidelines provided by SOP 024 version 4; *Reporting of SAEs, SUSARs and periodic safety reports*, with the exception of causality attribution and classification which will be based on the following criteria:

- **No relationship**
  - No temporal relationship to vaccine administration or *S*. Typhi ingestion, ***and***
  - Alternative aetiology (clinical, environmental or other intervention), ***and***
  - Does not follow pattern of recognised response to vaccine administration or typhoid fever.
- **Possible**
  - Reasonable temporal relationship to vaccine administration or *S*. Typhi ingestion, ***or***
  - Event not readily explained by alternative aetiology (clinical, environmental or other interventions), ***or***
  - Similar pattern of response to that seen to vaccine administration or typhoid fever.
- **Probable**
  - Reasonable temporal relationship to vaccine administration or *S*. Typhi ingestion, ***and***

  - Event not readily produced by alternative aetiology (clinical, environment, or other interventions), ***or***
  - Known pattern of response with vaccine administration or typhoid fever

- **Definite**
  - Reasonable temporal relationship to vaccine administration or *S.* Typhi ingestion; ***and***
  - Event not readily produced by alternative aetiology (clinical, environment, or other interventions), ***and***
  - Known pattern of response to vaccine administration or typhoid fever.

Severity grading criteria for adverse events

Severity grading of adverse events will be assisted using recommendations from the U.S. Department of Health and Human Services, Food and Drug Administration. [93]

Severity for solicited AEs will be graded as per **Table 3**.

Severity grading for laboratory abnormalities will be as per **Table 4**.

Severity grading for vital signs will be as per **Table 5**.

Unsolicited AEs will be graded as per **Table 6**.

Table 3: Grading of solicited AE severity

		Event severity (grade)			
		**Mild (1)**	**Moderate (2)**	**Severe (3)**	**Potentially life-threatening (4)**
**Adverse event**	Nausea/ vomiting	No interference with activity or 1–2 episodes/24 hours	Some interference with activity or >2 episodes/24 hours	Prevents daily activity, requires outpatient IV hydration	Emergency department visit or hospitalization for hypotensive shock
	Anorexia	Eats less than normal for 1-2 meals	Misses 1-2 meals completely	Does not eat all meals	Emergency department visit or Hospitalisation
	Abdominal pain	No interference with activity	Some interference with activity	Significant; prevents daily activity	Emergency department visit or Hospitalisation
	Headache	No interference with activity	Some interference with activity	Significant; prevents daily activity	Emergency department visit or Hospitalisation
	Malaise	No interference with activity	Some interference with activity	Significant; prevents daily activity	Emergency department visit or Hospitalisation
	Myalgia	No interference with activity	Some interference with activity	Significant; prevents daily activity	Emergency department visit or Hospitalisation
	Arthralgia	No interference with activity	Some interference with activity	Significant; prevents daily activity	Emergency department visit or Hospitalisation
	Cough	No interference with activity	Some interference with activity	Significant; prevents daily activity	Emergency department visit or Hospitalisation
	Diarrhoea	2–3 loose stools/24 hours	4–5 stools/24 hours	6 or more watery stools/24 hours or requires outpatient IV hydration	Emergency department visit or Hospitalisation
	Constipation	No interference with activity	Some interference with activity	Significant; prevents daily activity	Emergency department visit or Hospitalisation
	Flatulence	No increase from normal	Some increase from normal	Significant increase; interferes with daily activity	N/A

Rash will classified as present or absent and further described in the case report form by a study investigator.

Table 4: Severity grading for laboratory solicited AEs (* Potentially life-threatening; RRT: requires Renal Replacement Therapy)

		Event severity			
		**Grade 1**	**Grade 2**	**Grade 3**	**Grade 4***
**Laboratory test (units)**	Haemoglobin (female): decrease from baseline value (gm/dl)	any decrease-1.5	1.6-2.0	2.1-5.0	>5
	Haemoglobin (male): decrease baseline value (gm/dl)	any decrease-1.5	1.6-2.0	2.1-5.0	>5
	White cell count: increase cell/mm^3	10,800–15,000	15,001–20,000	20,001–25,000	>25,000
	White cell count: decrease (cells/mm^3)	2500-3500	1500-2499	1000-1499	<1000
	Neutrophil count (cells/mm^3)	1500-2000	1000-1499	500-999	<500
	Platelets (cells/mm^3)	125,000-140,000	100,000-124,000	25,000-99,000	<25,000
	Sodium: hyponatraemia (mEq/L)	132–134	130–131	125–129	<125
	Sodium: hypernatraemia (mEq/L)	144–145	146–147	148–150	>150
	Potassium: hyperkalaemia (mEq/L)	5.1–5.2	5.3–5.4	5.5–5.6	>5.6
	Potassium: hypokalaemia mEq/L	3.5–3.6	3.3–3.4	3.1–3.2	<3.1
	Urea (mmol/L)	8.2–8.9	9.0–11	>11	RRT
	Creatinine (µmol/L)	132-150	151-176	177-221	>221 or RRT
	ALT, AST	1.1–2.5 x ULN	>2.6–5.0 x ULN	5.1-10 x ULN	>10 x ULN
	Bilirubin (with increase in LFTs)	1.1–1.25 x ULN	1.26–1.5 x ULN	1.51–1.75 x ULN	>1.75 x ULN
	Bilirubin (with normal LFTs)	1.1–1.5 x ULN	1.6–2.0 x ULN	2.0–3.0 x ULN	>3.0 x ULN
	Alkaline	1.1–2.0 x ULN	2.1–3.0 x ULN	3.1–10 x ULN	>10 x ULN
	Amylase	1.1–1.5 x ULN	1.6–2.0 x ULN	2.1–5.0 x ULN	>5.0 x ULN
	Albumin: hypoalbuminaemia (g/L)	28–31	25–27	<25	N/A
	C-reactive protein	>10-30	31-100	100-200	>200

Table 5: Severity grading of clinical examination findings

		Finding severity			
		**Grade 1**	**Grade 2**	**Grade 3**	**Grade 4***
**Finding**[†]	Fever (^{o}C)[‡]	38.0-38.4	38.5-38.9	39.0-40.0	>40
	Tachycardia (beats per minute)	101-115	116-130	>130	Emergency department visit or hospitalisation for arrhythmia
	Bradycardia (beats per minute)[¥]	50-54	45-49	<45	Emergency department visit or hospitalisation for arrhythmia
	Hypertension: systolic blood pressure (mmHg), with repeat testing at the same visit	141-150	151-155	>155	Emergency department visit or hospitalisation for malignant hypertension
	Hypertension: diastolic blood pressure (mmHg)	91-95	96-100	>100	Emergency department visit or hospitalisation for malignant hypertension
	Hypotension: systolic blood pressure (mmHg), with repeat testing at the same visit	85-89	80-84	<80	Emergency department visit or hospitalisation for hypotensive shock
	Respiratory rate (breaths per minute)	17-20	21-25	>25	Intubation

* Potentially life-threatening; [†] Participants should be at rest for measurement of vital signs; [‡] Oral temperature; no recent hot or cold beverages or smoking; [¥] When resting heart rate is between 60-100 beats per minute - use clinical judgement when characterising bradycardia among some healthy participant populations, for example, conditioned athletes.

Table 6: Grading of unsolicited AEs

Scale	Description	Definition
**1**	Mild	Transient or mild discomfort (<48 hours); no medical intervention/ therapy required
**2**	Moderate	Some interference with activity not requiring medical intervention
**3**	Severe	Prevents daily activity and requires medical intervention
**4**	Serious	Life threatening

Reporting procedures for Serious Adverse Events

A Data and Safety Monitoring Committee (DSMC) will be convened to review both vaccine and challenge-related SAEs for the study. They will meet at regular intervals and consider issues including:

- Occurrence and nature of adverse events,
- Whether additional information on adverse events is required,
- Consider taking appropriate action where necessary to halt the study,
- Act/advise on incidents occurring between meetings that require rapid assessment (e.g., SUSARs).

A summary of all SAEs will be reported to the DSMC at 3-monthly intervals from the date of first vaccine administration. In addition any Adverse Events that the Chief Investigator (CI) considers to be of safety concern to the study (including complications of typhoid fever, relapse or development of chronic carrier state and secondary transmission of typhoid fever) will be reported to the DSMC within 24 hours of discovery. All SAE information must be recorded on the University of Oxford SAE forms and faxed or e-mailed to the DSMC. Additional information received for a case (follow-up or corrections to the original case) need to be detailed on a new SAE form and submitted to the DSMC. In addition, the host NHS Trust will be informed of any admission of a participant to hospital for the management of severe typhoid fever and will receive a copy of all SAE reports sent to the DSMC.

All SARs (including SUSARs) will be reported within 24 hours. The CI will report all SUSARs to the DSMC, sponsor, the relevant Research Ethics Committee and to the MHRA. Fatal or life-threatening SUSARs must be reported within 7 days and all other SUSARs within 15 days.

In addition to the expedited reporting above, the CI shall submit once a year throughout the clinical trial or on request, an Annual Progress Report to the Ethics Committee, host NHS Trust and sponsor. Additionally a Development Safety Update Report (DSUR) will be submitted to the MHRA, relevant Ethics Committee, host NHS Trust (R&D department) and to the sponsor.

All SAEs and SUSARs will be notified to Emergent BioSolutions within 24 hours of the CI becoming aware of their occurrence. Similarly, all vaccine-related AEs will be notified within 7 days.[viii]

[viii] e-mail: TyphellaProductSafety@ebsi.com, fax: +44(0)118-944-3301

Procedure to be followed in the event of abnormal findings

Abnormal clinical findings from medical history, examination or blood tests, will be assessed as to their clinical significance using the above tables. If a test result is deemed clinically significant, it may be repeated, to ensure it is not a single occurrence. If a test remains clinically significant, the participant will be informed and appropriate medical care arranged with the permission of the participant. Decisions to exclude potential participants from enrolling in the trial or to withdraw a participant from the trial will be at the discretion of the investigators.

Study Management Committee

The study investigators will form the study management committee and will provide day-to-day management of the study.

Trial Steering Committee

A Trial Steering Committee (TSC) will be appointed and will consist of an independent Chairman, not less than two other independent members and the Chief Investigator. The TSC will meet regularly and will provide overall supervision of the trial on behalf of the Trial Sponsor and ensure that the trial is conducted to the rigorous standards set out in the Medical Research Council's (MRC), Guidelines for Good Clinical Practise. In particular the TSC will provide guidance on the progress of the trial, adherence to the protocol, participant safety and the consideration of new information of relevance to the trial. The TSC will provide guidance thorough its chair to the Chief Investigator and the trial sponsor.

Data and Safety Monitoring Committee

A Data and Safety Monitoring Committee (DSMC) will be appointed to provide real-time safety oversight. The DSMC will be notified of all SAEs at 3-monthly intervals, and of all SARs (including SUSARs) within 24 hours of their occurrence and will undertake to perform a timely review of these as per a pre-agreed DSMC charter. The DSMC will inform the Chairperson of the TSC immediately to recommend termination of the study if deemed necessary following an SAR. The DSMC will have access to un-blinded data and will monitor these data and make recommendations to the TSC on whether there are any ethical or safety reasons why the trial should not continue. In particular, they will have access to data relating to the Ty21a positive control arm, so that the validity of the challenge model can be determined in order to prevent inadvertent continuation and enrolment of unnecessary participants.

The DSMC will be a minimum of two independent advisors and a statistician. The chair of the DSMC will be contacted for advice and independent review in the following situations:

- Following any SAR
- Any other situation where the Investigator feels independent advice or review is important

12. STAFF AND INVESTIGATOR SAFETY

All staff working on the project will be advised to attend occupational health to ensure their vaccination status against typhoid fever is up-to-date. Information will be made available to all potential staff either working on the study or exposed to study specimens by means of a staff information booklet. This will provide details relating to, for example, the GMO and challenge strain and provide further contact information and action points to be used as required.

13. STATISTICAL PLAN

Statistical hypothesis

The primary objective of this study is to determine the relative protective effect of M01ZH09 vaccine compared to vaccine placebo using a healthy adult typhoid challenge model. The null and alternate hypotheses are:

$H_0 = TD_{Placebo} = TD_{M01ZH09}$

$H_1 = TD_{Placebo} \neq TD_{M01ZH09}$

Where $TD_{Placebo/M01ZH09}$ is the proportion of participants given a diagnosis of typhoid infection (see section 0 for definition) who have been vaccinated with vaccine placebo or M01ZH09 vaccine, respectively.

Sample size and power considerations

Based on findings from the previous dose finding study performed at OVG,[75] an infective dose (inoculum) will be used to achieve an attack rate of 60-75%. Assuming a similar attack rate (which is likely to be around 60%) in the group vaccinated with placebo here, then to demonstrate a protective effect of 83%, resulting in a reduction in attack rate to 10%, 21 participants would be needed per group (see **Table 7**). If the attack rate in the placebo vaccinated group falls to 50% in this study, then 30 participants would be needed per group to demonstrate a protective effect of vaccination of 80% with 90% power (1-β) at the 5% significance level (α). A broadly similar degree of protection and resultant reduction in attack rate was demonstrated during studies evaluating the Ty21a vaccine by the University of Maryland in challenge studies performed in the 1960s/70s.

Although a correlate of protection has not yet been found for typhoid fever, a 4-fold or greater increase in anti-typhoid LPS or flagellin antibody has previously been used as a surrogate marker.[63] Using this endpoint, a response rate of 75% to vaccination with M01ZH09 was seen. Therefore, if the attack rate in the vaccinated arm is as high as 30% and 70% in the placebo arm, 36 or more participants would be needed per group (see table below).

Therefore, at least 33 individuals will be enrolled per group (including a 10% dropout rate),

Table 7. Power calculation

Salmonella Typhi attack rate		Sample size requirement per group				
**M01ZH09 vaccine**	**Placebo vaccine**	**1-β:**	**90%**	**90%**	**80%**	**80%**
		**α:**	**5%**	**1%**	**5%**	**1%**
10%	30%		92	126	71	102
10%	40%		48	66	38	54
10%	50%		30	41	24	34
10%	60%		21	28	17	24
10%	70%		15	20	12	17
10%	80%		11	15	9	13
10%	90%		8	11	7	10
10%	100%		6	8	5	7
20%	40%		118	164	91	131
20%	50%		57	79	45	64
20%	60%		34	47	27	38
20%	70%		22	31	18	25
20%	80%		16	21	13	18
20%	90%		11	15	9	13
20%	100%		8	11	7	9
30%	60%		62	86	48	69
30%	70%		36	49	28	40
30%	80%		22	31	18	25
30%	90%		15	20	12	17
30%	100%		10	14	9	12

Populations for analysis

As the participants to be recruited are healthy adult controls and the primary objective is to establish the absolute protection afforded by the IMP vaccine, M01ZH09, the Per Protocol (PP) population will be used for evaluation of the primary endpoint. For the current study, the PP population is defined as,

All participants who:

- Have signed the requisite consent forms and successfully the completed screening assessments, and
- Have had the requisite paperwork completed and agreed by their general practitioner, and
- Have received the allocated study vaccine (or the actual vaccine received in case of randomisation error), and
- Have been successfully challenged with the challenge organism, and
- Have received no bias or interference that may interfere with potential vaccine effect or infection challenge, either according to the protocol or in the view of the study investigators.

Description of the final population to be analysed for the primary endpoint will be reported in accordance with the CONsolidated Standards of Reporting Trials (CONSORT) Statement.[94].

If a participant later withdraws from the study, data up until that point will be included in the analysis. If participants are withdrawn before it is determined whether or not they develop typhoid within the specified 14 day period after being challenged, then sensitivity analyses will be undertaken to explore different assumptions for the missing data.

Secondary endpoints will be analysed in the following populations:

- **Post-vaccination symptoms** – exposed population (those having actually received a vaccine or placebo) providing post-vaccination symptom data, without a major violation (a protocol violation considered by the investigators to have an impact (quantitative or qualitative)) which may have an effect on symptom reporting.
- **Post-vaccination immune responses** – per protocol population (PP), vaccine response; exposed population supplying at least one evaluable post-vaccination clinical specimen, without a major violation (a protocol violation considered by the investigators to have an impact (quantitative or qualitative)) which may have an effect on the immunological response.
- **Post-challenge symptoms** – exposed and challenged population (those having taken the *S.* Typhi suspension in the required manner) providing post-challenge symptom data according to protocol, without a major violation (a protocol violation considered by the investigators to have an impact (quantitative or qualitative)) which may have an effect on symptom reporting.
- **Post-challenge immune responses** – per protocol population (PP), challenge response; exposed and challenged population supplying at least one evaluable clinical specimen, without a major violation (a protocol violation considered by the investigators to have an impact (quantitative or qualitative)) which may have an effect on the immunological response.

The laboratory analysis of some parameters may be terminated before samples from all time points have been analysed if it is felt that further analysis was not of scientific value. This is likely to be if it has already been demonstrated that parameters for any endpoint have returned to baseline. The decision to not process latter time points will be at the discretion of the Principle Investigator. Further exploratory analysis may be conducted if findings of scientific interest become apparent during the study or processing of the data. Any deviations from the statistical plan detailed here will be described and justified in the final report, however, all data obtained will be listed in the final study report.

Analysis of demographic and baseline characteristics

Descriptive statistics relating to participant characteristics at screening will be calculated overall and by group:

- Continuous variables, including age, height, weight, number of cigarettes smoked (by pack-year history) and number of units of alcohol consumed per week; will be analysed by mean, standard deviation, median, interquartile range and range.
- Continuous variables measured at each visit including clinical data (blood pressure, temperature, etc.) will be calculated and analysed by visit number using the same parameters as above.
- Categorical and binary data will be analysed by participant distribution; these will include gender, age-group, ethnic origin, smoking and drinking status.

Analysis of study endpoints

Statistical method for the primary endpoint

The proportion of participants with a diagnosis of typhoid fever (i.e. the attack rate) and the associated 95% confidence intervals will be presented by group and at each time point (each visit between days 3-14). The primary endpoint will use the cumulative numbers diagnosed in each group by day 14. The difference in proportions between the M01ZH09 and vaccine placebo groups will be analysed using Pearson's chi-squared test with continuity correction (or Fisher's Exact test if appropriate). To fulfill the primary objective, the protective effect of M01ZH09 over placebo will be calculated by:

$$PE = 100 \times (AR_{placebo} – AR_{M01ZH09})/AR_{Placebo} = 100 \times (1 – AR_{M01ZH09}/AR_{Placebo}),$$

where PE is the protective effect and AR is attack rate.

The 95%CI of the $AR_{M01ZH09}/AR_{Placebo}$ can be calculated using standard methods for calculation of 95%CI on a rate ratio. The corresponding 95%CI for PE will also be calculated.

The attack rate in the Ty21a vaccinated arm will be compared to that in the placebo arm to confirm integrity of the challenge model in comparison to previously performed studies.[67-69] There will be no formal comparison between the protective efficacies of the IMP and positive control vaccines.

Statistical methods for the secondary endpoints

To fulfil the secondary objectives, further analysis of secondary endpoints will be performed by comparison of infected and un-infected participants in vaccine or vaccine-placebo exposed group. Parametric continuous variables will be summarised using mean, standard

deviation and range values (after log transformation, if required, for assumption of normality, in which case the original geometric means and confidence intervals will also be presented); non-parametric variables (or variables far from normal distribution) using median, interquartile range and range values. Categorical values will be presented as counts, proportions and/or centiles. 95% confidence intervals will be calculated where relevant.

The specific statistical calculations to be performed and the relevant methodologies will be described separately in a detailed statistical analysis plan formulated in collaboration with the Centre for Statistics in Medicine. A summary of the key assessments is given in brief below.

Clinical

The clinical course of illness in participants will be described and presented by vaccine group, in particular:

- The time interval between challenge and onset of disease. The following parameters will be used:
    - Mean time from dosing to the development of a temperature ≥38^{0}C for ≥12 hours in participants in whom this occurs.
    - Mean time to *Salmonella* Typhi bacteraemia (detected by positive blood culture) in participants in whom this occurs.
- The symptom profile of participants meeting the definition of typhoid fever, with symptoms including:
    - Malaise
    - Headache
    - Myalgia/ Arthralgia
    - Anorexia/ Loss of appetite
    - Nausea/ Vomiting
    - Flatulence
    - Constipation/ Diarrhoea
    - Abdominal pain
    - Cough
    - Rash
    - Fever (oral temperature)
    - The time of onset, duration and severity of the above symptoms in those who meet the definition of typhoid fever.
    - The proportion of participants developing severe typhoid infection (defined as defined in section 0).

Inflammatory

A comparison of the inflammatory changes after challenge between vaccine groups will be made. Parameters assessed as differences (or fold-changes where specified) will include:

- A comparison of the mean rise in CRP from day 0 to days 5, 8, 10, 12 and 14 in those who meet the definition of typhoid fever and those who do not.

- A comparison of the mean rise in pro-inflammatory cytokines from the pre-challenge serum to hour 12, days 1, 3, 5, 6, 7, 10, 14, 28, 60, 90, 180, 365 and years 2 and year 3.
- A comparison of the mean CRP 24, 48 and 96 hours after typhoid diagnosis.
- A comparison of the maximum fold rise from baseline in CRP in those who meet the definition of typhoid fever.
- A comparison of the mean rise in faecal lactoferrin levels on days 0, 4, 7, 10, 14, 18, 21, and 28 in those who meet the definition of typhoid fever and those who do not.

Microbiology

A comparison of the microbiological parameters between infected and uninfected participants in each vaccine group, including:

- Comparison of the mean concentration of *Salmonella* Typhi CFU in stool and blood on days 1-15.
- A comparison of the proportion of participants who develop *Salmonella* Typhi bacteraemia.
- A comparison of the number of participants that have *Salmonella* Typhi detectable in stool on days 2-15.

Functional genomics

Changes in gene expression profiles will be assessed using commercial array-analysis software, incorporating:

1. Image analysis – with removal or marking of poor-quality and low-intensity features.
2. Data processing – with background subtraction, determination of spot intensities and intensity ratios, and log-transformation of ratios, global or local normalization of intensity ratios.

Alteration in gene expression will be assessed in infected and uninfected participants by allocated vaccine group, including calculation of the relative abundance of gene expression (measured by gene expression microarrays and/or mRNA-seq technologies), after vaccination (at baseline, 7, 14 and 28 days)[ix] and after challenge (at 12 and 24 hours and on days 3 to 28, 60, 90, 180 and years 1, 2 and 3).

[ix] Additional time points to be assessed for the functional genomics sub-group at days -32, -30, -28, -26 and -24 for the Ty21a arm and -28, -26 and -24 for the M01ZH09/placebo arm.

Diagnostics

Comparison will be made between the numbers and characteristics of participants diagnosed with typhoid infection using the standard microbiological and clinical definitions and the new, novel diagnostic tests including PCR based assays and urine mass spectrometry. Comparison will be made to blood culture results provided by the ORH microbiology laboratories, which will be considered the 'gold-standard'. The sensitivity, specificity, positive- and negative-predictive value, and the corresponding 95% CI for each test will be calculated.

Immunology

In order to assess the immunological responses after vaccination and during and after challenge with *S.* Typhi, measurements will be compared in infected and uninfected participants in each vaccine group to further identify correlates of protection from typhoid infection and to improve future typhoid diagnostic tests. These will be further described in the statistical analysis plan.

Safety and tolerability

Accounting for multiple comparisons following vaccination, the following vaccine safety data will be reported for each group for all individuals actually receiving a vaccination or the vaccine placebo:

- The total number and proportion of participants experiencing each type of adverse event
- The total number and proportion of participants experiencing at least one adverse event of any type

Adverse events by severity and relationship to vaccine group will be tabulated and summarised and the number of participants with reported serious adverse events up to 10 days after each vaccination will also be detailed, with 95% confidence intervals.

Level of statistical significance

The main study outcome is a proportion which will be quoted along with 95% confidence intervals. Selective reporting of outcomes (on a descriptive basis) will be avoided if practical; otherwise there will be honesty in which ones have been omitted, so that any selective reporting is apparent. Outcome variables relating to the primary and secondary endpoints will be reported by group, as defined above (section 0; post-vaccination symptoms, post-vaccination immune responses, post-challenge symptoms and post-challenge immune responses). Those found not to be statistically significant will not be reported unless of clinical interest/utility, however all data will be listed in the final study report.

Criteria for termination of study

The Chief Investigator and Data Safety Monitoring Committee will have the right to terminate the study at any time. If the study is prematurely terminated the investigator will promptly inform the participants and will ensure appropriate therapy and follow-up. If the study is halted, the MHRA and relevant Ethics Committee will be notified within 15 days of this occurring.

Accounting for missing, unused or spurious data.

Reasons for missing data (including withdrawal of consent, loss to follow-up, removal from study due to serious side effects, death, or inability to obtain any laboratory results) will be indicated but missing data will not be imputed. The quantity of missing data for each vaccine group and the appertaining demographic characteristics will be compared. There will be an intention to publish all collected data or at least open clarification about which additional variables have been measured if reporting is ultimately selective, so that readers can self-determine the possible impact of “data dredging”, i.e. selective reporting of seemingly interesting results.

Participants who are withdrawn before the challenge visit will be replaced. Participants withdrawn after challenge may be replaced at the discretion of the Chief Investigator.

Procedure for reporting deviation from the original statistical plan

Any additional analysis or deviations from the analysis plan will be documented and updated according to the statistical standard operating procedure.

14. ACCESS TO SOURCE DATA AND STUDY DOCUMENTS

Direct access will be granted to authorised representatives from the sponsor and host institution and the regulatory authorities to permit trial-related monitoring, audits and inspections.

15. QUALITY CONTROL AND QUALITY ASSURANCE PROCEDURES

The study will be conducted in accordance with the current approved protocol, ICH GCP, relevant regulation and the OVG standard operating procedures.

Regular monitoring will be performed according to ICH GCP, as per section 0. Data will be evaluated for compliance with the protocol and accuracy in relation to source documents. Following written standard operating procedures, the monitors will verify that the study is conducted and data are generated, documented and reported in compliance with the protocol, GCP and applicable regulatory requirements.

Protocol deviations

Any deviations from the protocol will be documented using a protocol deviation form and filed in the trial master file.

16. ETHICS

Declaration of Helsinki

The Investigator will ensure that this study is conducted in accordance with the principles in the Declaration of Helsinki of the World Medical Association.

ICH Guidelines for Good Clinical Practice

The Investigator will ensure that this study is conducted in full conformity with relevant regulations and with the ICH Guidelines for Good Clinical Practice (CPMP/ICH/135/95) July 1996.

Approvals

The protocol, informed consent form, participant information sheet and any proposed advertising material will be submitted to an appropriate Research Ethics Committee (REC) and the Oxford Radcliffe Hospitals NHS Trust Research and Development Committee. The protocol and further information as required will be provided to the medicines regulatory authorities (MHRA in the UK) and the Department for the Environment, Food and Rural Affairs (DEFRA).

The Investigator will submit and, where necessary, obtain approval from the above parties for all substantial amendments to the original approved documents.

Participant Confidentiality

The trial staff will ensure that the participants' anonymity is maintained. The participants will be identified by initials and a participants ID number on the CRF and any electronic database. All documents will be stored securely and only accessible by trial staff and authorised personnel. The study will comply with the Data Protection Act which requires data to be anonymised as soon as it is practical to do so. If admitted to the John Warin Ward, participants will have NHS notes generated that will contain their names, date of birth and address. Samples sent to the ORH Laboratories during their admission will contain relevant details required by the NHS for processing of clinical specimens. Ultrasound requests to the radiology department sent before or at initial screening visit will contain limited personal demographic details, as required by the department. Permission will be sought from the participant for this limited disclosure of their details. Any notes generated during an in-patient stay or NHS attendance, and the radiology report will be subject to the confidentiality policy of the ORH NHS Trust.

Compensation for harm

Negligent Harm: Indemnity and/or compensation for negligent harm arising specifically from an accidental injury for which the University is legally liable as the Research Sponsor will be covered by the University of Oxford. The NHS will owe a duty of care to those undergoing clinical treatment, with Trust Indemnity available through the NHS Litigation Authority Scheme.

Non-Negligent Harm: Indemnity and/or compensation for harm arising specifically from an accidental injury, and occurring as a consequence of the Research Subjects' participation in the trial for which the University is the Research Sponsor will be covered by the University of Oxford.

17. DATA HANDLING AND RECORD KEEPING

The investigators will enter the data into the participants' CRFs, which will be in a paper and/or electronic format (using an OpenClinica™ database stored on a secure University of Oxford server). This includes safety data, laboratory data (both clinical and immunological) and outcome data. Data is entered in a web browser on PCs in the CCVTM building and then transferred to the OpenClinica Database by encrypted (Https) transfer.

CRFs will include, but not limited to, the following:

- Participants contact details (to be retained at the Oxford Vaccine Group)
- Inclusion and exclusion criteria
- Medical history
- Concomitant medication
- Details (including results) of baseline investigations
- Details of challenge with *Salmonella* Typhi
- Details of each investigation (for example, blood test)
- Details of each follow up visit
- Study termination record for subjects completing per protocol and for earlier withdrawals
- Diary cards completed by participants

Each study participant will have a unique screening number which will be allocated following the taking of informed consent. At the point of allocation of vaccine or placebo a separate study number will be allocated. For each participant, sufficient labels with the same study number will be generated to label all CRFs, diary cards and blood sample tubes.

Participants requiring admission to the John Warin Ward will be have NHS notes generated that will record details of their admission. Relevant details will be transcribed into the CRF. Samples sent to ORH laboratories for inpatients will be labelled with the participant's hospital number.

In order to identify study staff completing each CRF, a signature sheet, including full name and initials of all trial staff will be used.

A study database will be constructed to record the information collected in the CRFs and diary cards. If clarification of any adverse events is required or completion errors or omissions are noted, then a member of study staff will make the correction on the CRFs, crossing out any incorrect information with a single line, and will sign and date the change on

the original CRF. Corrections to the diary cards will be made by the participant by means of crossing out any incorrect information with a single line, and signing and dating the correction.

The participants will be identified by a study specific participants number and/or code in any database. The name and any other identifying detail will NOT be included in any study data electronic file.

Blinding of laboratory samples

Samples sent to ORH laboratories for processing will be identified by trial number and participant number but will not be blinded. Samples received by the OVG laboratory will be similarly but will not be marked with any participant identifying data.

Samples sent to collaborating laboratories will be identified by a laboratory number only. A random list of laboratory numbers will be generated by an independent statistician that will be linked to participant number on a password encoded file. The blinding code will be broken when required for data analysis.

Data integrity

Data collection and storage will be inspected throughout the study by internal (performed by the Oxford Vaccine Group) and external (by the study sponsor, University of Oxford, CTRG) monitoring.

Data archiving and storage

Following completion of the study, all personal data will be kept for a period of 15 years by Ardington Archives storage (Faringdon, Oxford) according to the OVG SOP 028 version 3; *Archiving*, Storage of this data will be reviewed every 5 years and files will be confidentially destroyed if storage is no longer required after 7 years.[x] Due to the nature of the study it is necessary for the data to be stored for this period of time so that participants can be contacted should new information be made available. Prof Andrew J Pollard, or his successor, as Head of the Oxford Vaccine Group will have the responsibility for custody of the data.

[x] As per the terms of the Emergent Supply Agreement (section 7.1d).

18. FINANCE AND INSURANCE

Insurance

See section 0.

Funding

Emergent BioSolutions has provided financial assistance for the original GMP manufacture of the *Salmonella* Typhi challenge strain by the HPA, and for the provision of the vaccines used in this study. Funding for the study has been provided by a Wellcome Trust Translation Award ("Acceleration of the development of vaccines and diagnostics for typhoid fever using a human challenge model"). For details, see funding agreements with The University of Oxford.

Compensation

All participants will be compensated for their time and for the inconvenience based on the following figures:

- Travel expenses: £15 per visit;
- Inconvenience of blood tests: £10;
- Time required for visit: £20 per visit;
- Time off work compensation: £150 per day for 14 days (total £2100).

Participants who agree to attend additional visits for samples to be taken for functional genomics will be compensated an additional £25/ visit (£15 travel expenses, £10 compensation for inconvenience of blood test).

Thus, a participant allocated to the positive control (Ty21a) arm not developing typhoid fever and completing all study visits, would receive a total of:

Visits	Costs	Value (£)
Screening visit plus ultrasound scan	15+20+10+20	65
3 vaccination visits	15x3+20x3+10	115
24 follow-up visits	15x24+20x24+10x24	1080
Time off work compensation	150x14	2100
TOTAL		3360

A participant allocated to the vaccine placebo group developing a diagnosis of typhoid fever at day 10 and completing all further study visits would receive:

Visits	Costs	Value (£)
Screening visit plus ultrasound scan	15+20+10+20	65
1 vaccination visits	15+20+10	45
26 follow-up visits (including 7 post-typhoid diagnosis)	15x26+20x26+10x26	1170
Time off work compensation	150x14	2100
TOTAL		3380

Payments will be provided to the participant at visit 14, three months, 6 months and then at each subsequent visit until the study is complete (or following the scheduled date for this visit if this were not to be completed).

19. PUBLICATION POLICY

The Chief Investigator will co-ordinate dissemination of data from this study. All publications (including manuscripts, abstracts, oral/slide presentations and book chapters) based on the vaccine efficacy study will be submitted to each sub-investigator and Emergent BioSolutions for review a minimum of 30 days prior to submission.[xi]

[xi] As per the terms of the Emergent Supply Agreement (section 10.1).

20. REFERENCES

1. Crump, J.A., Luby, S.P. & Mintz, E.D. The global burden of typhoid fever. *Bull World Health Organ* **82**, 346-353 (2004).
2. Bhan, M.K., Bahl, R. & Bhatnagar, S. Typhoid and paratyphoid fever. *Lancet* **366**, 749-762 (2005).
3. Simanjuntak, C.H.*, et al.* Oral immunisation against typhoid fever in Indonesia with Ty21a vaccine. *Lancet* **338**, 1055-1059 (1991).
4. Sinha, A.*, et al.* Typhoid fever in children aged less than 5 years. *Lancet* **354**, 734-737 (1999).
5. Lin, F.Y.*, et al.* The epidemiology of typhoid fever in the Dong Thap Province, Mekong Delta region of Vietnam. *Am J Trop Med Hyg* **62**, 644-648 (2000).
6. Ackers, M.L., Puhr, N.D., Tauxe, R.V. & Mintz, E.D. Laboratory-based surveillance of Salmonella serotype Typhi infections in the United States: antimicrobial resistance on the rise. *JAMA* **283**, 2668-2673 (2000).
7. Steinberg, E.B.*, et al.* Typhoid fever in travelers: who should be targeted for prevention? *Clin Infect Dis* **39**, 186-191 (2004).
8. Reller, M.E.*, et al.* Sexual transmission of typhoid fever: a multistate outbreak among men who have sex with men. *Clin Infect Dis* **37**, 141-144 (2003).
9. Parry, C.M. & Threlfall, E.J. Antimicrobial resistance in typhoidal and nontyphoidal salmonellae. *Curr Opin Infect Dis* **21**, 531-538 (2008).
10. Crump, J.A., Ram, P.K., Gupta, S.K., Miller, M.A. & Mintz, E.D. Part I. Analysis of data gaps pertaining to Salmonella enterica serotype Typhi infections in low and medium human development index countries, 1984-2005. *Epidemiol Infect* **136**, 436-448 (2008).
11. Mirza, S.H., Beeching, N.J. & Hart, C.A. Multi-drug resistant typhoid: a global problem. *J Med Microbiol* **44**, 317-319 (1996).
12. Rowe, B., Ward, L.R. & Threlfall, E.J. Multidrug-resistant Salmonella typhi: a worldwide epidemic. *Clin Infect Dis* **24 Suppl 1**, S106-109 (1997).
13. Parry, C., Wain, J., Chinh, N.T., Vinh, H. & Farrar, J.J. Quinolone-resistant Salmonella typhi in Vietnam. *Lancet* **351**, 1289 (1998).
14. Parry, C.M. The treatment of multidrug-resistant and nalidixic acid-resistant typhoid fever in Viet Nam. *Trans R Soc Trop Med Hyg* **98**, 413-422 (2004).
15. Murdoch, D.A.*, et al.* Epidemic ciprofloxacin-resistant Salmonella typhi in Tajikistan. *Lancet* **351**, 339 (1998).
16. Gupta, A. Multidrug-resistant typhoid fever in children: epidemiology and therapeutic approach. *Pediatr Infect Dis J* **13**, 134-140 (1994).
17. Whitaker, J.A., Franco-Paredes, C., del Rio, C. & Edupuganti, S. Rethinking typhoid fever vaccines: implications for travelers and people living in highly endemic areas. *J Travel Med* **16**, 46-52 (2009).
18. Parry, C.M., Hien, T.T., Dougan, G., White, N.J. & Farrar, J.J. Typhoid fever. *N Engl J Med* **347**, 1770-1782 (2002).
19. Ochiai, R.L.*, et al.* Salmonella paratyphi A rates, Asia. *Emerg Infect Dis* **11**, 1764-1766 (2005).
20. Palit, A.*, et al.* Increasing prevalence of Salmonella enterica serotype Paratyphi-A in patients with enteric fever in a periurban slum setting of Kolkata, India. *Int J Environ Health Res* **16**, 455-459 (2006).
21. Sood, S.*, et al.* Paratyphoid fever in India: An emerging problem. *Emerg Infect Dis* **5**, 483-484 (1999).
22. Thaver, D.*, et al.* Fluoroquinolones for treating typhoid and paratyphoid fever (enteric fever). *Cochrane Database Syst Rev*, CD004530 (2008).
23. Threlfall, E.J., de Pinna, E., Day, M., Lawrence, J. & Jones, J. Alternatives to ciprofloxacin use for enteric Fever, United kingdom. *Emerg Infect Dis* **14**, 860-861 (2008).
24. Effa, E.E. & Bukirwa, H. Azithromycin for treating uncomplicated typhoid and paratyphoid fever (enteric fever). *Cochrane Database Syst Rev*, CD006083 (2008).

25. Maskey, A.P., *et al.* Emerging trends in enteric fever in Nepal: 9124 cases confirmed by blood culture 1993-2003. *Trans R Soc Trop Med Hyg* **102**, 91-95 (2008).
26. Engels, E.A., Falagas, M.E., Lau, J. & Bennish, M.L. Typhoid fever vaccines: a meta-analysis of studies on efficacy and toxicity. *Bmj* **316**, 110-116 (1998).
27. Sur, D., *et al.* A cluster-randomized effectiveness trial of Vi typhoid vaccine in India. *N Engl J Med* **361**, 335-344 (2009).
28. Acharya, I.L., *et al.* Prevention of typhoid fever in Nepal with the Vi capsular polysaccharide of Salmonella typhi. A preliminary report. *N Engl J Med* **317**, 1101-1104 (1987).
29. Cook, J., Sur, D., Clemens, J. & Whittington, D. Evaluating investments in typhoid vaccines in two slums in Kolkata, India. *J Health Popul Nutr* **27**, 711-724 (2009).
30. Yang, H.H., *et al.* An outbreak of typhoid fever, Xing-An County, People's Republic of China, 1999: estimation of the field effectiveness of Vi polysaccharide typhoid vaccine. *J Infect Dis* **183**, 1775-1780 (2001).
31. Weintraub, A. Immunology of bacterial polysaccharide antigens. *Carbohydr Res* **338**, 2539-2547 (2003).
32. Landy, M. Studies on Vi antigen. VI. Immunization of human beings with purified Vi antigen. *Am J Hyg* **60**, 52-62 (1954).
33. Mond, J.J., Lees, A. & Snapper, C.M. T cell-independent antigens type 2. *Annu Rev Immunol* **13**, 655-692 (1995).
34. Klugman, K.P., *et al.* Protective activity of Vi capsular polysaccharide vaccine against typhoid fever. *Lancet* **2**, 1165-1169 (1987).
35. Yang, H.H., *et al.* Efficacy trial of Vi polysaccharide vaccine against typhoid fever in south-western China. *Bull World Health Organ* **79**, 625-631 (2001).
36. Michel, R., *et al.* Outbreak of typhoid fever in vaccinated members of the French Armed Forces in the Ivory Coast. *Eur J Epidemiol* **20**, 635-642 (2005).
37. Froeschle, J.E. & Decker, M.D. Duration of Vi antibodies in participants vaccinated with Typhim Vi (Typhoid Vi polysaccharide vaccine) in an area not endemic for typhoid fever. *Vaccine* (2009).
38. Pasetti, M.F., Simon, J.K., Sztein, M.B. & Levine, M.M. Immunology of gut mucosal vaccines. *Immunol Rev* **239**, 125-148 (2011).
39. Cryz, S.J., Jr., *et al.* Safety and immunogenicity of Salmonella typhi Ty21a vaccine in young Thai children. *Infect Immun* **61**, 1149-1151 (1993).
40. Centers for Disease Control and Prevention; Division of Foodborne, B.a.M.D. Typhoid Fever: General Information. Vol. 2011 (ed. CDC) (Atlanta, 2010).
41. Olanratmanee, T., Levine, M., Losonsky, G., Thisyakorn, V. & Cryz, S.J., Jr. Safety and immunogenicity of Salmonella typhi Ty21a liquid formulation vaccine in 4- to 6-year-old Thai children. *J Infect Dis* **166**, 451-452 (1992).
42. Levine, M.M., Ferreccio, C., Black, R.E. & Germanier, R. Large-scale field trial of Ty21a live oral typhoid vaccine in enteric-coated capsule formulation. *Lancet* **1**, 1049-1052 (1987).
43. Levine, M.M., Ferreccio, C., Cryz, S. & Ortiz, E. Comparison of enteric-coated capsules and liquid formulation of Ty21a typhoid vaccine in randomised controlled field trial. *Lancet* **336**, 891-894 (1990).
44. Levine, M.M., Ferreccio, C., Black, R.E., Tacket, C.O. & Germanier, R. Progress in vaccines against typhoid fever. *Rev Infect Dis* **11 Suppl 3**, S552-567 (1989).
45. Szu, S.C., *et al.* Laboratory and preliminary clinical characterization of Vi capsular polysaccharide-protein conjugate vaccines. *Infect Immun* **62**, 4440-4444 (1994).
46. Lin, F.Y., *et al.* The efficacy of a Salmonella typhi Vi conjugate vaccine in two-to-five-year-old children. *N Engl J Med* **344**, 1263-1269 (2001).
47. Way, J.S., *et al.* Specific detection of Salmonella spp. by multiplex polymerase chain reaction. *Appl Environ Microbiol* **59**, 1473-1479 (1993).
48. Robbins, J.B. & Schneerson, R. Polysaccharide-protein conjugates: a new generation of vaccines. *J Infect Dis* **161**, 821-832 (1990).

49. Pulickal, A.S. & Pollard, A.J. Vi polysaccharide-protein conjugate vaccine for the prevention of typhoid fever in children: hope or hype? *Expert Rev Vaccines* **6**, 293-295 (2007).
50. Cui, C., *et al.* Physical and chemical characterization and immunologic properties of Salmonella enterica serovar typhi capsular polysaccharide-diphtheria toxoid conjugates. *Clin Vaccine Immunol* **17**, 73-79 (2010).
51. Micoli, F., *et al.* Vi-CRM 197 as a new conjugate vaccine against Salmonella Typhi. *Vaccine* **29**, 712-720 (2011).
52. Rondini, S., *et al.* Evaluation of the immunogenicity and biological activity of Citrobacter freundii Vi CRM197 conjugates as a vaccine for S. Typhi. *Clin Vaccine Immunol* (2011).
53. Spreng, S., Dietrich, G. & Weidinger, G. Rational design of Salmonella-based vaccination strategies. *Methods* **38**, 133-143 (2006).
54. Mekmullica, J. & Pancharoen, C. Acceptability of oral typhoid vaccine in Thai children. *Southeast Asian J Trop Med Public Health* **34**, 334-336 (2003).
55. Harrington, P.M., Woodman, C. & Shannon, W.F. Vaccine, yes; injection, no: maternal responses to the introduction of Haemophilus influenzae type b (Hib) vaccine. *Br J Gen Pract* **49**, 901-902 (1999).
56. Tacket, C.O., *et al.* Safety of live oral Salmonella typhi vaccine strains with deletions in htrA and aroC aroD and immune response in humans. *Infect Immun* **65**, 452-456 (1997).
57. Lowe, D.C., *et al.* Characterization of candidate live oral Salmonella typhi vaccine strains harboring defined mutations in aroA, aroC, and htrA. *Infect Immun* **67**, 700-707 (1999).
58. Wahid, R., Salerno-Goncalves, R., Tacket, C.O., Levine, M.M. & Sztein, M.B. Cell-mediated immune responses in humans after immunization with one or two doses of oral live attenuated typhoid vaccine CVD 909. *Vaccine* **25**, 1416-1425 (2007).
59. Levine, M.M. Typhoid fever vaccines. in *New generation vaccines* (eds. Levine, M.M., Kaper, J.B., Rappuoli, R., Liu, M.A. & Good, M.F.) 1057-1093 (Informa Healthcare, London, 2004).
60. Ltd, E.P.D.U. M01ZH09 Investigators Brochure. (Wokingham, 2010).
61. Ochman, H., Soncini, F.C., Solomon, F. & Groisman, E.A. Identification of a pathogenicity island required for Salmonella survival in host cells. *Proc Natl Acad Sci U S A* **93**, 7800-7804 (1996).
62. Coombes, B.K., Wickham, M.E., Lowden, M.J., Brown, N.F. & Finlay, B.B. Negative regulation of Salmonella pathogenicity island 2 is required for contextual control of virulence during typhoid. *Proc Natl Acad Sci U S A* **102**, 17460-17465 (2005).
63. Kirkpatrick, B.D., *et al.* Evaluation of Salmonella enterica serovar Typhi (Ty2 aroC-ssaV-) M01ZH09, with a defined mutation in the Salmonella pathogenicity island 2, as a live, oral typhoid vaccine in human volunteers. *Vaccine* **24**, 116-123 (2006).
64. Lyon, C.E., *et al.* In a randomized, double-blinded, placebo-controlled trial, the single oral dose typhoid vaccine, M01ZH09, is safe and immunogenic at doses up to 1.7 x 10(10) colony-forming units. *Vaccine* **28**, 3602-3608 (2010).
65. Tran, T.H., *et al.* A randomised trial evaluating the safety and immunogenicity of the novel single oral dose typhoid vaccine M01ZH09 in healthy Vietnamese children. *PLoS One* **5**, e11778 (2010).
66. Sztein, M.B. Cell-mediated immunity and antibody responses elicited by attenuated Salmonella enterica Serovar Typhi strains used as live oral vaccines in humans. *Clin Infect Dis* **45 Suppl 1**, S15-19 (2007).
67. Song, J.H., *et al.* Detection of Salmonella typhi in the blood of patients with typhoid fever by polymerase chain reaction. *J Clin Microbiol* **31**, 1439-1443 (1993).
68. Hornick, R.B., *et al.* Typhoid fever: pathogenesis and immunologic control. 2. *N Engl J Med* **283**, 739-746 (1970).
69. Hornick, R.B., *et al.* Typhoid fever: pathogenesis and immunologic control. *N Engl J Med* **283**, 686-691 (1970).

70. Gilman, R.H., *et al.* Evaluation of a UDP-glucose-4-epimeraseless mutant of Salmonella typhi as a liver oral vaccine. *J Infect Dis* **136**, 717-723 (1977).
71. Levine, M.M., *et al.* Attenuated, streptomycin-dependent Salmonella typhi oral vaccine: potential deleterious effects of lyophilization. *J Infect Dis* **133**, 424-429 (1976).
72. Gotuzzo, E., *et al.* Use of norfloxacin to treat chronic typhoid carriers. *J Infect Dis* **157**, 1221-1225 (1988).
73. Gotuzzo, E., *et al.* Ciprofloxacin for the treatment of cholera: a randomized, double-blind, controlled clinical trial of a single daily dose in Peruvian adults. *Clin Infect Dis* **20**, 1485-1490 (1995).
74. Ferreccio, C., *et al.* Efficacy of ciprofloxacin in the treatment of chronic typhoid carriers. *J Infect Dis* **157**, 1235-1239 (1988).
75. Waddington, C. Understanding Typhoid Disease: developing a human challenge model of Salmonella Typhi in healthy humans (OVG 2009/10). Study protocol. (University of Oxford, 2010).
76. Levine, M.M., Taylor, D.N. & Ferreccio, C. Typhoid vaccines come of age. *Pediatr Infect Dis J* **8**, 374-381 (1989).
77. Wahdan, M.H., *et al.* A controlled field trial of liver oral typhoid vaccine Ty21a. *Bull World Health Organ* **58**, 469-474 (1980).
78. Tacket, C.O., *et al.* Safety and immune responses to attenuated Salmonella enterica serovar typhi oral live vector vaccines expressing tetanus toxin fragment C. *Clin Immunol* **97**, 146-153 (2000).
79. Ivanoff, B., Levine, M.M. & Lambert, P.H. Vaccination against typhoid fever: present status. *Bull World Health Organ* **72**, 957-971 (1994).
80. Committee., J.F. British National Formulary. (ed. Society, B.M.A.a.R.P.) (London, 2011).
81. Service, T.N.B. Can I give blood? , Vol. 2011 (NHS Blood and Transplant, 2011).
82. Josephson, K.L., Gerba, C.P. & Pepper, I.L. Polymerase chain reaction detection of nonviable bacterial pathogens. *Appl Environ Microbiol* **59**, 3513-3515 (1993).
83. Zhuang, R.Y. & Beuchat, L.R. Effectiveness of trisodium phosphate for killing Salmonella montevideo on tomatoes. *Lett Appl Microbiol* **22**, 97-100 (1996).
84. Black, R.E., *et al.* Case-control study to identify risk factors for paediatric endemic typhoid fever in Santiago, Chile. *Bull World Health Organ* **63**, 899-904 (1985).
85. Infection, W.G.o.t.f.P.A.C.o.G. Preventing person-to-person spread following gastrointestinal infections: guidelines for public health pysicians and environmental health officers. *Commun Dis Public Health* **7**, 362-384 (2004).
86. Thomas, H.L., Addiman, S. & Mellanby, A. Evaluation of the effectiveness and efficiency of the public health management of cases of infection due to Salmonella typhi/paratyphi in North East London. *Public Health* **120**, 1188-1193 (2006).
87. Braddick, M.R. & Sharp, J.C. Enteric fever in Scotland 1975-1990. *Public Health* **107**, 193-198 (1993).
88. Hodgetts, A., Levin, M., Kroll, J.S. & Langford, P.R. Biomarker discovery in infectious diseases using SELDI. *Future Microbiol* **2**, 35-49 (2007).
89. Wada, H., Ono, T., Uenaka, A., Monden, M. & Nakayama, E. Requirement of CD4+ T cells and antigen-presenting cells for primary in vitro generation of CD8+ cytotoxic T cells against Ld-binding self-peptide p2Ca. *Immunology* **84**, 633-637 (1995).
90. Papale, M., *et al.* Urine profiling by SELDI-TOF/MS: monitoring of the critical steps in sample collection, handling and analysis. *J Chromatogr B Analyt Technol Biomed Life Sci* **856**, 205-213 (2007).
91. Crucell UK. Summary of Product Characteristics, Vivotif (Ty21a live oral attenuated vaccine). (2010).
92. Commission, E. EU Guidelines to Good Manufacturing Practice Medicinal Products for Huamn and Veterinary Use. Vol. 4 (ed. Pharmaceuticals) (Brussels, 2010).
93. U.S. Department of Health and Human Services, F.a.D.A. Guidance for Industry. Toxicity grading scale for healthy adult and adolescent volunteers enrolled in

preventive vaccine clinical trials. (ed. Office of Communication, T.a.M.A.) (FDA, Rockville, 2007).
94. Moher, D., *et al.* CONSORT 2010 explanation and elaboration: updated guidelines for reporting parallel group randomised trials. *BMJ* **340**, c869 (2010).

APPENDIX 1: GMP DEVELOPMENT OF A *SALMONELLA* TYPHI CHALLENGE AGENT FOR CLINICAL USE

(i) Aims

- To generate a working cell bank (WCB) to GMP.
- To develop a manufacturing process to produce a frozen vial of challenge strain (*Salmonella Typhi* Quailes strain) between 10^3 and 10^7 CFU/ml.
- To develop the necessary testing to ensure identity, purity and content.
- To determine the stability characteristics of the challenge strain and assign a clinical expiry.
- To document the development and manufacture to ensure quality and traceability.

(ii) Development strategy

The development of a typhoid challenge agent will take place under several discrete steps as detailed in **Figure 1**.

a). Cell banking, assay and manufacturing process development at Oxford

As previous media used to propagate the cell line do not meet the current regulatory guidelines for TSE-free media, it will be necessary to undertake a regime diluting existing media components through a process of repeated sub-cultures. During this procedure all sub-culturing will occur in liquid culture to reduce the risk of clonal selection.

The number and timings of these sub-cultures will be determined after the growth characteristics have been established. Three rounds of shake flask grow up (inoculating 100ml TSA with 100μl inoculum from previous flask) will be undertaken.

At the end of the final round of sub-culturing, a 200 vial master cell bank is generated. The final shake flask will be used to inoculate a shake flask containing 200ml TSA. This flask will be grown to an OD600nm of 1.0 at which point 50ml of 80% (w/v) glycerol is added (to give a final concentration of 16% (w/v), mixed and 1ml aliquots added to 2ml cryo-vials. This cell bank is labelled as OX-SQ-MCB1 (Oxford-*Salmonella Typhi* Quailes Strain-Master Cell Bank 1). The OX-SQ-MCB1 will be tested for identity, purity and content

Figure 1. Development of *S.* Typhi challenge agent

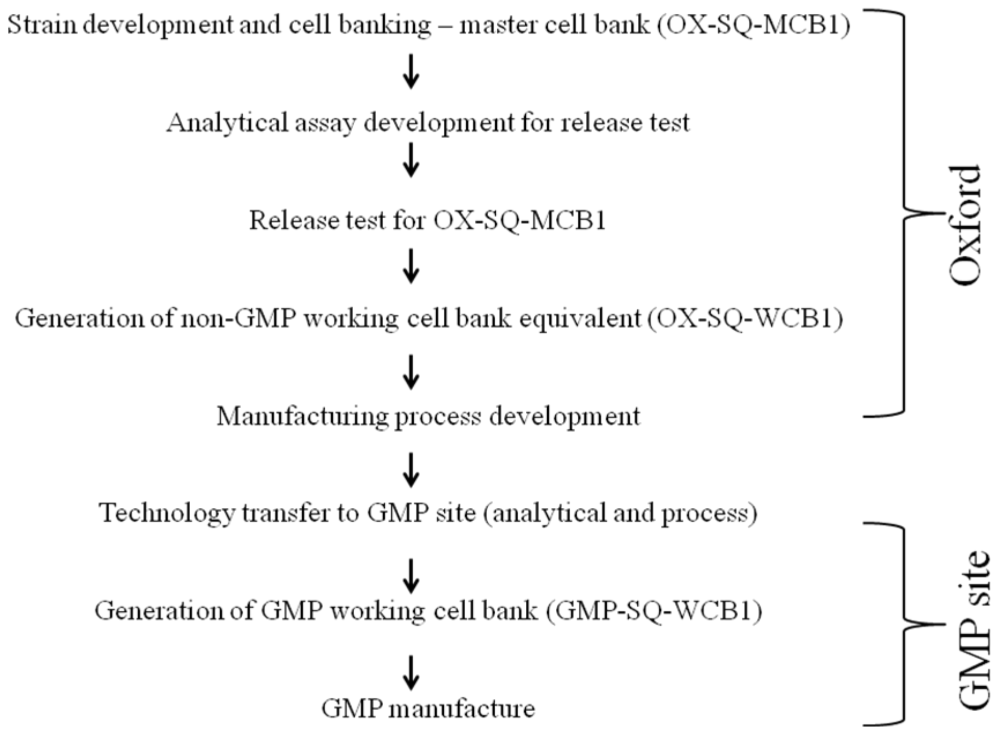

Analytical assays required as detailed in **Figure 2** will be developed at Oxford, and used for the cell bank preparation and process development activities. These include identity assays (API20E, antibiotic sensitivity, slide agglutination), purity assays (microbial limits and gross contamination) and a content assay (viable cell counts).

In order to conduct process development work at Oxford, it will be necessary to make an equivalent cell bank to the GMP bank (GMP-SQ-WCB1). This bank will be made at Oxford to the same protocol as the GMP-SQ-WCB1 and will be known as OX-SQ-WCB1. The release tests of this bank are outlined in **Figure 2**, but will mirror those stated for the GMP-SQ-WCB1. Process development will be performed at Oxford using OX-SQ-WCB1 prior to transfer to the GMP manufacturing site.

The initial phase will consider the growth characteristics of the strain in liquid medium in shake flask. Once the growth characteristics have been determined and the final density of bacteria achievable from the shake flask is known, the next phase of development is the dilution and formulation phase. The culture is diluted by mixing with sterile media plus sucrose solution to a final concentration of 10 %(w/w) sucrose in the product. The formulated solution is then stored at 2-8°C for 19 hours, while a VCC is performed. The resulting VCC will indicate if a further dilution is required with media+10% (w/w) sucrose to give the appropriate dose, prior to freezing at -80°C for storage and then when required delivery to the clinical site.

Once the process development has been concluded a process description will be generated to outline to the GMP manufacturing site, how to perform the manufacture and the process will undergo formal technology transfer to a GMP manufacturing facility. A complete run of the manufacturing run at Oxford will be used to generate stability data for the frozen manufactured vials. Samples will be taken at defined time points to evaluate the stability of the frozen product. The process will be documented in a process description document.

b). Technology transfer from Oxford to GMP manufacturing site

Once the manufacturing development has been documented into a process description document and verification of analytical assays has been completed, a formal Technology Transfer will take place for Analytical assays, working cell bank preparation and the manufacturing process.

Following analytical transfer, 10 vials of OX-SQ-MCB1 will be shipped to a GMP site for the production of a GMP working cell bank (GMP-SQ-WCB1) using the same procedure used to generate OX-SQ-WCB1 at Oxford. The release testing of this GMP bank is the same as for OX-SQ-WCB1, including identity, purity and content tests.

It would be expected that 3 manufacturing runs would be required for the tech transfer to be demonstrated, with the final batch acting as a qualification batch and placed on stability.

(iii) GMP manufacture

Following successful Technology Transfer to the GMP manufacturing site, a single manufacturing run will be performed to GMP. The release testing of the challenge strain product will be conducted. The vials will be labelled with a regulatory acceptable label. A number of frozen vials of final product will be retained by the GMP manufacturing site and placed on stability. Following review of the batch manufacturing data for quality issues and successful release testing, the challenge strain product vials will be shipped from the GMP site back to Oxford under suitably controlled conditions. On receipt of the challenge strain product at Oxford, the vials will be stored under suitably controlled conditions to ensure quality and comparability to the material stored at the GMP site for stability.

a). Analytical assays and specifications

During the development of manufacture of the challenge strain, a number of assays will be used to ensure the quality of the final product. **Figure 2** identifies which assays are required for testing intermediate and final cell banks, the final product and process development activities. Assays performed on non-GMP cell banks will be established but are not required to be verified. All assays performed on GMP materials will be verified assays.

Figure 2. Analysis required during the development of manufacture of the challenge strain

	Identity		Purity		Content	
	API-20E (+ Gram stain)	Antibiotic sensitivity (MIC)	Slide Agglutinations	Microbial limits	Gross contamination	VCC (viable cell count)
**Cell banks**						
UM-SQ-EXP1	✓	✓	✓	✓	✓	✓
OX-SQ-MCB1	✓	✓	✓	✓	✓	✓
OX-SQ-WCB1	✓	✓	✓	✓	✓	✓
GMP-SQ-WCB1	✓	✓	✓	✓	✓	✓
**Final Product release**						
Challenge strain product	✓	✓	✓	✓		✓
