# Supplementary material for: Using a Human Challenge Model of Infection to Measure Vaccine Efficacy: A Randomised, Controlled Trial Comparing the Typhoid Vaccines M01ZH09 with Placebo and Ty21a
Source: PLoS Negl Trop Dis. 2016 Aug 17;10(8):e0004926. doi: 10.1371/journal.pntd.0004926 (PMC4988630; doi:10.1371/journal.pntd.0004926)

S1 Figure. Challenge doses dispensed to 92 participants according to batch (co-challenged participants) and typhoid challenge outcome.

TD, typhoid diagnosed, filled circles; nTD Typhoid not diagnosed, clear circles.

Challenge dose administered was measured by direct plating from the challenge suspension onto tryptone soya agar (Oxoid) prior to colony counting after 24 hours incubation (37°C, 5%CO_2). Challenge doses ranged from 1.46-2.66x10^4CFU *S.* Typhi Quailes strain; median dose given was 1.82x10^4 CFU denoted by dashed horizontal line.

Scheduling of participants to receive challenge occurred at their convenience but in batches so far as possible, to enable logistic configuration of laboratory and clinical staffing. Batch size ranged from 1-10 participants (median *n*=4, IQR 2-5); all participants within a batch were challenged on the same day and with the same challenge dose.

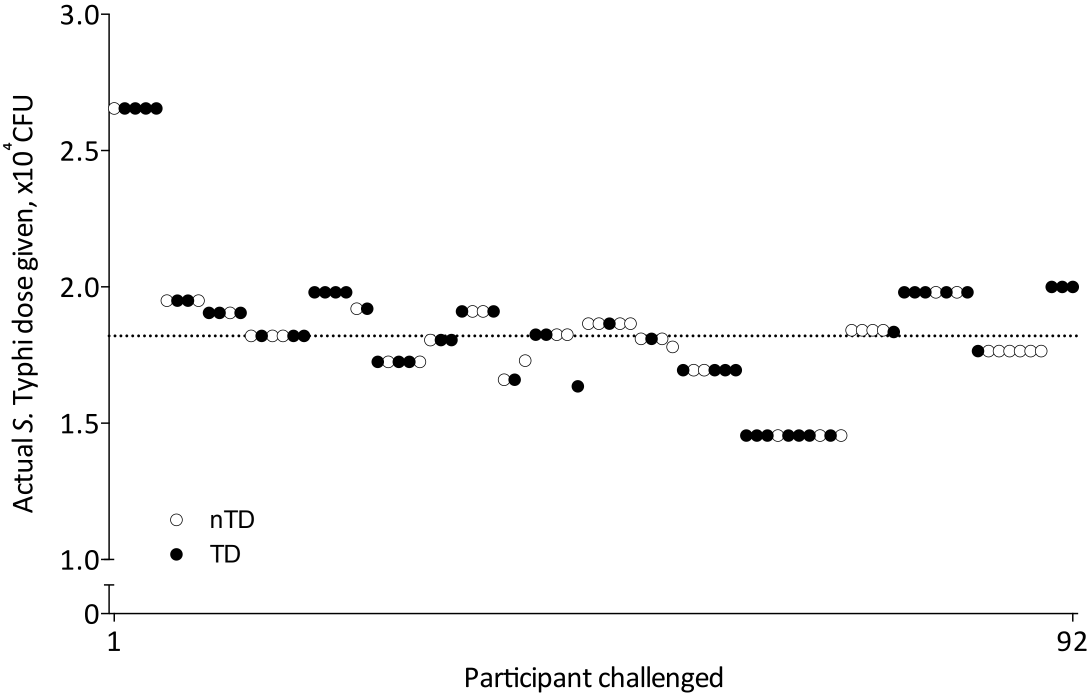

Supplement: S1 Fig — TD, typhoid diagnosed, filled circles; nTD Typhoid not diagnosed, clear circles. Challenge dose administered was measured by direct plating from the challenge suspension onto tryptone soya agar (Oxoid) prior to colony counting after 24 hours incubation (37°C, 5%CO2). Challenge doses ranged from 1.46–2.66x10^4^CFU S. Typhi Quailes strain; median dose given was 1.82x10^4^ CFU denoted by dashed horizontal line. Scheduling of participants to receive challenge occurred at their convenience but in batches so far as possible, to enable logistic configuration of laboratory and clinical staffing. Batch size ranged from 1–10 participants (median n = 4, IQR 2–5); all participants within a batch were challenged on the same day and with the same challenge dose. (PDF) [file pntd.0004926.s004.pdf]
