# Supplementary material for: Using a Human Challenge Model of Infection to Measure Vaccine Efficacy: A Randomised, Controlled Trial Comparing the Typhoid Vaccines M01ZH09 with Placebo and Ty21a
Source: PLoS Negl Trop Dis. 2016 Aug 17;10(8):e0004926. doi: 10.1371/journal.pntd.0004926 (PMC4988630; doi:10.1371/journal.pntd.0004926)
Supplement: S1 Table — Severity is mean score. Symptoms in bold text represent the ‘classical triad’ of typhoid fever presentation: fever, headache and abdominal pain. (PDF) [file pntd.0004926.s005.pdf]

	VACCINATION						CHALLENGE					
	**M01ZH09** (*n*=32) (Blind)		**Placebo** (*n*=29) (Blind)		**Ty21a** (*n*=30) (Open-label)		**M01ZH09** (*n*=32) (Blind)		**Placebo** (*n*=30) (Blind)		**Ty21a** (*n*=30) (Open-label)	
	% (95% CI)	Severity	% (95% CI)	Severity	% (95% CI)	Severity	% (95% CI)	Severity	% (95% CI)	Severity	% (95% CI)	Severity
**Fever (>38°)**	0	NA	0	NA	0	NA	**44 (26-62)**	**1.28**	**53 (34-72)**	**1.53**	**30 (15-49)**	**0.70**
**Headache**	31 (16-50)	0.44	41 (24-61)	0.57	30 (15-49)	0.48	**72 (53-86)**	**1.61**	**90 (73-98)**	**2.10**	**63 (44-80)**	**1.23**
Generally unwell	28 (14-47)	0.31	31 (15-51)	0.39	30 (15-49)	0.34	69 (50-84)	1.52	87 (69-96)	2.06	67 (47-83)	1.27
Loss of appetite	13 (4-29)	0.25	24 (10-44)	0.29	17 (6-35)	0.17	56 (38-74)	1.21	80 (61-92)	1.58	43 (25-63)	0.77
**Abdominal pain**	16 (5-33)	0.19	17 (6-36)	0.28	20 (8-39)	0.21	**56 (38-74)**	**1.00**	**60 (41-77)**	**0.97**	**47 (28-66)**	**0.80**
Nausea/vomiting	19 (7-36)	0.25	29 (13-47)	0.32	10 (2-27)	0.10	44 (26-62)	0.82	60 (41-77)	1.03	33 (17-53)	0.60
Myalgia	0	NA	14 (4-32)	0.21	17 (6-35)	0.28	44 (26-62)	0.94	67 (47-83)	1.35	53 (34-72)	0.87
Arthralgia	9 (2-25)	0.13	10 (2-27)	0.14	10 (2-27)	0.14	38 (21-56)	0.70	57 (37-75)	0.90	37 (20-56)	0.60
Cough	25 (11-43)	0.31	14 (4-32)	0.18	7 (1-22)	0.07	41 (24-59)	0.52	43 (25-63)	0.45	47 (28-66)	0.57
Diarrhoea	19 (7-36)	0.28	10 (2-27)	0.11	13 (4-31)	0.14	13 (4-29)	0.24	40 (23-59)	0.58	20 (8-39)	0.23
Constipation	13 (4-29)	0.16	21 (8-40)	0.21	13 (4-31)	0.21	44 (26-62)	0.85	57 (37-75)	0.94	33 (17-53)	0.67

S3 Table. Frequency of solicited reports of adverse events during the first 7 days after vaccine administration and during the first 21 days after challenge, according to vaccine group allocation.

Severity is mean score. Symptoms in bold text represent the 'classical triad' of typhoid fever presentation: fever, headache and abdominal pain.
