# Supplementary material for: Using a Human Challenge Model of Infection to Measure Vaccine Efficacy: A Randomised, Controlled Trial Comparing the Typhoid Vaccines M01ZH09 with Placebo and Ty21a
Source: PLoS Negl Trop Dis. 2016 Aug 17;10(8):e0004926. doi: 10.1371/journal.pntd.0004926 (PMC4988630; doi:10.1371/journal.pntd.0004926)
Supplement: S2 Table — Geometric mean concentration, GMC (95%CI). Lower limit-of-detection, 0.25 cells/106PBMC. PBMC, peripheral blood mononuclear cells. GMC, measured in ASC/106 peripheral blood mononuclear cells (PBMC). (PDF) [file pntd.0004926.s006.pdf]

Sample time point	M01ZH09 (*n*=33) Day -28	Day -21	Placebo (*n*=30) Day -28	Day -21	Ty21a (*n*=30) Day -28	Day -21
**Anti-LPS isotype ASC/10^6PBMC**						
**IgG**	0.7 (0.5-1.2)	12.7 (5.9-27.4)	1.1 (0.7-1.8)	1.3 (0.7-2.5)	0.9 (0.5-1.6)	2.2 (1.4-3.6)
**IgA**	1.6 (0.9-2.7)	84.1 (48.9-144.7)	2.1 (1.2-3.5)	2.0 (1.2-3.4)	1.9 (1.1-3.5)	11.1 (6.5-19.1)
**IgM**	0.9 (0.5-1.4)	95.9 (59.4-154.7)	1.4 (0.8-2.3)	1.4 (0.8-2.3)	1.2 (0.7-2.1)	8.3 (4.9-14.0)
**Anti-H isotype ASC/10^6PBMC**						
**IgG**	0.6 (0.4-0.9)	20.7 (10.7-40.3)	0.5 (0.3-0.7)	0.6 (0.4-0.9)	0.6 (0.4-0.8)	2.1 (1.3-3.5)
**IgA**	0.9 (0.6-1.5)	90.0 (57.9-166.0)	0.8 (0.5-1.2)	1.5 (0.9-2.4)	1.2 (0.7-2.0)	12.6 (7.6-21.1)
**IgM**	0.9 (0.6-1.3)	131.9 (89-195.6)	0.5 (0.3-0.8)	1.1 (0.7-1.6)	0.9 (0.6-1.4)	8.4 (4.8-14.6)
**Anti-Vi isotype ASC/10^6PBMC**						
**IgG**	0.6 (0.4-1.0)	0.8 (0.5-1.3)	0.9 (0.5-2.5)	1.4 (0.8-2.5)	1.1 (0.6-2.1)	1.1 (0.6-1.8)
**IgA**	0.8 (0.5-1.2)	2.2 (1.3-3.9)	0.8 (0.5-1.2)	1.0 (0.6-1.7)	0.9 (0.5-1.6)	1.0 (0.6-1.8)
**IgM**	0.9 (0.5-1.4)	1.4 (0.8-2.3)	1.3 (0.8-2.2)	1.4 (0.8-2.3)	1.3 (0.7-2.2)	1.6 (1.0-2.7)

S4 Table. Plasma antibody-secreting cell responses to vaccination with M01ZH09, placebo or Ty21a.

Geometric mean concentration, GMC (95%CI). Lower limit-of-detection, 0.25 cells/10^6PBMC. PBMC, peripheral blood mononuclear cells. GMC, measured in ASC/10^6 peripheral blood mononuclear cells (PBMC).
