# Supplementary material for: Using a Human Challenge Model of Infection to Measure Vaccine Efficacy: A Randomised, Controlled Trial Comparing the Typhoid Vaccines M01ZH09 with Placebo and Ty21a
Source: PLoS Negl Trop Dis. 2016 Aug 17;10(8):e0004926. doi: 10.1371/journal.pntd.0004926 (PMC4988630; doi:10.1371/journal.pntd.0004926)
Supplement: S3 Table — Geometric mean titre (95%CI). Lower limit-of-antibody detection, 7.4EU/mL. (PDF) [file pntd.0004926.s007.pdf]

	M01ZH09 (*n*=32)		Placebo (*n*=30)		Ty21a (*n*=29)	
**Sample time point**	**Day -28**	**Day 0**	**Day -28**	**Day 0**	**Day -28**	**Day 0**
**Anti-LPS isotypes, EU/mL**						
**IgG**	83<br>(62 – 111)	222<br>(146 – 339)	86<br>(63 – 118)	84<br>(62 – 113)	118<br>(86 – 161)	166<br>(107 – 258)
**IgA**	48<br>(36 – 63)	86<br>(62 – 118)	51<br>(38 – 69)	51<br>(39 – 68)	65<br>(46 – 92)	78<br>(53 – 116)
**IgM**	94<br>(76 – 116)	156<br>(126 – 192)	116<br>(90 – 149)	112<br>(87 – 145)	105<br>(85 – 130)	115<br>(95 – 140)
**Anti-H isotypes, EU/mL**						
**IgG**	41<br>(31 – 53)	113<br>(73 – 174)	42<br>(31 – 57)	44<br>(33 – 59)	48<br>(33 – 69)	50<br>(35 – 72)
**IgA**	12<br>(9 – 15)	31<br>(23 – 43)	14<br>(11 – 18)	13<br>(11 – 17)	25<br>(18 – 33)	24<br>(17 – 32)
**IgM**	12<br>(9 – 16)	348<br>(181 – 668)	22<br>(16 – 32)	18<br>(13 – 26)	17<br>(12 – 24)	23<br>(16 – 32)
**Anti-Vi, EU/mL**						
**IgG**	6<br>(4 – 8)	6<br>(4 – 8)	7<br>(5 – 10)	8<br>(5 – 11)	8<br>(5 – 13)	8<br>(5 – 13)

S5 Table. Anti-LPS, anti-H, anti-Vi antibody responses to vaccination with M01ZH09, placebo or Ty21a.

Geometric mean titre (95%CI). Lower limit-of-antibody detection, 7.4EU/mL.
